# Functional Analysis of *NtZIP4B* and Zn Status-Dependent Expression Pattern of Tobacco *ZIP* Genes

**DOI:** 10.3389/fpls.2018.01984

**Published:** 2019-01-10

**Authors:** Anna Barabasz, Małgorzata Palusińska, Anna Papierniak, Maria Kendziorek, Katarzyna Kozak, Lorraine Elizabeth Williams, Danuta Maria Antosiewicz

**Affiliations:** ^1^Faculty of Biology, Institute of Experimental Plant Biology and Biotechnology, University of Warsaw, Warsaw, Poland; ^2^Biological Sciences, University of Southampton, Southampton, United Kingdom

**Keywords:** cadmium, expression analysis, GUS assay, NtZIP4B, tobacco, zinc, ZIP genes

## Abstract

Tobacco is frequently considered as a plant useful for phytoremediation of metal-contaminated soil, despite the mechanisms for regulation of uptake and accumulation being largely unknown. Here we cloned and characterized a new tobacco Zn and Cd transporter *NtZIP4B* from the ZIP family (ZRT-IRT-Like proteins). It complemented the Zn-uptake defective yeast mutant *zrt1zrt2*, and rendered the wild type DY1457 yeast more sensitive to Cd. Bioinformatic analysis and transient expression of the NtZIP4B-GFP fusion protein in tobacco leaves indicated its localization to the plasma membrane. Real-time q-PCR based analysis showed that it is expressed in all vegetative organs with the highest level in leaves. The Zn status determined transcript abundance; *NtZIP4B* was upregulated by Zn-deficiency and downregulated by Zn excess. At the tissue level, in roots *NtZIP4B* is expressed in the vasculature of the middle part of the roots and in surrounding tissues including the root epidermis; in leaves primarily in the vasculature. Bioinformatic analysis identified two copies of *ZIP4* in tobacco, *NtZIP4A* and *NtZIP4B* with 97.57% homology at the amino acid level, with the same expression pattern for both, indicating a high degree of functional redundancy. Moreover, the present study provides new insights into the coordinated function of *NtZIP1, NtZIP2, NtZIP4, NtZIP5, NtZIP8, NtIRT1*, *and NtIRT1-like* in response to low-to-high Zn status. Leaves were the major site of *NtZIP4, NtZIP5*, and *NtZIP8* expression, and roots for *NtZIP1, NtZIP2, NtIRT1*, and *NtIRT1*-like. Contrasting expression level in the apical and basal root parts indicates distinct roles in root-specific processes likely contributing to the regulation of Zn root-to-shoot translocation. In summary, new insight into the role of *ZIP* genes in Zn homeostasis pointing to their overlapping and complementary functions, offers opportunities for strategies to modify Zn and Cd root/shoot partition in tobacco.

## Introduction

Heavy metal homeostasis relies on the operation of a wide variety of heavy metal transporters. These vary in their substrate specificity with some showing affinity for a broad range of metals while others display a greater level of specificity. Their metal regulation also varies. It has become evident, that the regulation of Zn homeostasis is not strictly specific for one metal only. By common pathways it is closely related to the homeostasis of other metals, both micronutrients including Fe, Mn, Ni, and also toxic Cd. This phenomenon has been termed “metal cross-homeostasis.” It is based on the regulation of a metal transport gene by more than one metal, and on diversified substrate specificity for metal transport proteins or chelating compounds (several metals with different affinity) (Sinclair and Krämer, 2012;Barabasz et al., 2013; Antosiewicz et al., 2014; Kendziorek et al., 2014).

The ZIP family (ZRT-IRT-like Proteins) includes broad substrate specificity membrane transporters shown to play a role in the transport of Zn, Fe, Mn, Cu, and also toxic Cd. Most of those ZIP proteins characterized already in plants are targeted to the plasma membrane, however, some of them are also found at the tonoplast or the endomembrane system. Thus they are involved in the regulation of both uptake, accumulation and translocation throughout the plant body of both micronutrients and toxic heavy metals (Palmer and Guerinot, 2009; Ricachenevsky et al., 2015).

There are 15 members of the ZIP family in *Arabidopsis thaliana* (Grotz and Guerinot, 2006), 23 in bean (Astudillo et al., 2013), 12 in *Poncirus trifoliata* (Fu et al., 2017), 15 in rice (Narayanan et al., 2007; Chen et al., 2008), 14 in wheat (Evens et al., 2017), or 12 in maize (Li et al., 2013; Mondal et al., 2014). However, only a limited number of the ZIP family members have been characterized in more detail with regard to their role *in planta*.

Our recent study on tobacco used suppression subtractive hybridization (SSH) to identify transporters involved in the regulation of Zn/Cd root/shoot distribution and led to cloning of a partial sequence of *NtZIP4* (Barabasz et al., 2016). *ZIP4* genes have been identified in *Arabidopsis thaliana, Medicago truncatula, Oryza sativa, Zea mays, Vitis vinifera, Poncirus trifoliata, Phaseolus vulgaris*, and *Vitis vinifera;* however, only fragmentary information is available on their localization, substrate specific or expression profiles (Wintz et al., 2003; López-Millán et al., 2004; Ishimaru et al., 2005; Gainza-Cortés et al., 2012; Astudillo et al., 2013; Li et al., 2013; Evens et al., 2017 Fu et al., 2017). The most extensively studied ZIP4 was from rice; which is localized to the plasma membrane and mediates Zn uptake (Ishimaru et al., 2005). The expression of *OsZIP4* was upregulated by low Zn, and the transcript was detected in the root and shoot meristem and in the vasculature, primarily in the phloem. It was considered to be involved in the control of Zn root-to-shoot translocation, and particularly important for Zn transport to seeds. Zn as a substrate was also shown for AtZIP4 and ZmZIP4, and in addition Cu for AtZIP4 and Fe for ZmZIP4, whereas for MtZIP4 Mn only. The ZIP4 genes were shown to be expressed both in roots and shoots in *Arabidopsis*, rice, *M. truncatula* and *V. vinifera* and upregulated by Zn-limiting conditions (Wintz et al., 2003; López-Millán et al., 2004; Gainza-Cortés et al., 2012; Li et al., 2013). Downregulation by Zn excess was shown for AtZIP4 (Jain et al., 2013). Furthermore, it was shown that AtZIP4 responds to the presence of Cd, and in the presence of 10 μM its expression in roots goes up (Jain et al., 2013). Interestingly, it was shown that expression of AtZIP4 (and also AtZIP1 and AtZIP3) from *A. thaliana* depends on the expression of Zn-responding WAKL4 (WAK-like kinase, WAK – cell wall associated receptor kinase), which clearly indicate the regulation of these ZIPs in response to cell-wall born signal generated by the presence of Zn (Hou et al., 2005).

Hence, in this study, to fill the gap in understanding the regulation of tobacco Zn homeostasis, the aim of this research was to clone and characterize NtZIP4 from tobacco. Tobacco is a plant important for phytoremediation of metal contaminated soil (Vangronsveld et al., 2009; Herzig et al., 2014), thus to improve its applicability for that purpose a comprehensive understanding of its metal transporters is necessary. Therefore, expression analysis of other known tobacco genes was performed to determine the possibly overlapping or complementary functions between them.

## Materials and Methods

### Plant Material and General Growth Conditions

Tobacco (*Nicotiana tabacum* var Xanthi) plants were grown in a controlled environment chamber at temperature 23/16°C day/night, 40–50% humidity, 16 h photoperiod, and quantum flux density [photosynthetically active radiation (PAR)] 250 mmol m-2 s-1, fluorescent Flora tubes. General conditions of plant cultivation were as described previously (Barabasz et al., 2010).

Seeds were surface sterilized in 8% sodium hypochlorite w/v for 2 min, then germinated and grown for 3 weeks on vertically positioned Petri dishes containing: quarter-strength Knop’s medium, 2% sucrose w/v and 1% agar w/v. Subsequently, plants were cultivated in hydroponic conditions using aeriated quarter-strength Knop’s medium as a standard, control solution. Three-week old seedlings were transferred from agar plates to 2 L pots (5 plant per pot) containing control liquid medium, and grown for a period indicated in each experiment. Solutions were changed every 3–4 days unless indicated otherwise in the description of the hydroponic experiment.

### Identification, Cloning and Characterization of ORF *NtZIP4B*

Previously, we identified a 480 bp sequence of *NtZIP4* transporter (accession no JZ875395.1, in the paper no T-XIII-K12) by SSH analysis (Barabasz et al., 2016). This sequence was used as a query in searching through the NCBI data base to find full-length *NtZIP4* gene/s from *N. tabacum*. Two sequences were identified: XM_016647965.1 with 99% homology to JZ875395 and named here as *NtZIP4A*; XM_016586154.1 with 97% homology and referred to as *NtZIP4B* (Supplementary File S1). Full-length *NtZIP4B* was amplified by PCR with Phusion HF polymerase (Thermo Scientific) using *cDNA* transcribed from total RNA isolated from roots of Zn-deficient plants [previously we found putative *NtZIP4* to be upregulated of under Zn-limiting conditions (Barabasz et al., 2016)]. NtZIP4B was amplified using the forward primers ZIP4B-ORF-START contained the CACC sequence at 5′ end (for directional cloning) and either of two reverse primers, one lacking the STOP codon (designated *ZIP4B*-END) and the second containing the STOP codon (designated *ZIP4B*-STOP). Primer sequences are given in the Supplementary File S1. The cDNA of *NtZIP4B*-STOP was cloned into Gateway entry vector. pENTR^TM^/D-TOPO^®^, and transformed into *E. coli* One Shot^TM^ TOP10 (Invitrogen). The insert was sequenced to confirm the correct sequence. These constructs were used for LR recombination into appropriate vectors for yeast complementation and transient expression assays (details in sections below).

### Bioinformatics Analysis

Translation of the nucleotide cDNA sequence of *NtZIP4A* and *NtZIP4B* to a protein sequence was performed by the use of ExPASy translate tool^1^. Alignment of NtZIP4A and NtZIP4B amino acid sequences and comparison with other ZIPs (sequences were identified in NCBI database with the use of BLAST algorithm) were performed using ClustalW. The phylogenetic tree was constructed with MEGA7.0 software (Tamura et al., 2013) using the maximum likelihood method with 1000 bootstrap replicates. The membrane-spanning regions and orientation was predicted based on Phobius software (Käll et al., 2004) and TMpred program^2^.

The NCBI database was used for BLASTn searches for putative, annotated *Nicotiana tabacum* *ZIP* sequences based on homology to already known *A. thaliana* sequences.

### Hydroponic Experiments

For experiments to determine the expression profiles of selected *ZIP* genes in tobacco, collected plant material was immediately frozen in the liquid nitrogen and stored in -80°C prior to mRNA isolation. All experiments were performed independently three times with three biological replicates per treatment. For each experiment plant material was collected and pooled from a total of 10 plants.

#### Growth of Plants for Determination of Expression Profile of ZIP Genes During Development

For expression analysis during development, roots and leaves were collected from four-week-old and six-week-old plants, grown for 3 weeks on agar plates then for 1 and 3 weeks, respectively, on hydroponics. More detailed studies were performed on 9-week-old plants (3 weeks on plates and 6 weeks on hydroponics). For expression analysis, apical and basal segments of roots (3–4 cm of the apical region and 3–4 cm of the basal region), stems (3 cm of the middle part), older leaves (two leaves counting from the base) and younger leaves (two leaves counting from the top) were collected.

#### Growth of Plants for Analysis of Zn- and Cd-Dependent Expression of Tobacco ZIP Genes

Plants of 5.5-weeks (3 weeks on plates followed by 2.5 weeks on hydroponics on control medium) were exposed to chosen concentrations of Zn and Cd for different times. To determine whether newly identified *NtZIP4A* and *NtZIP4B* and other chosen tobacco *ZIPs* are regulated by Zn-deficiency and Zn-excess, five-week-old tobacco plants were grown for 3 days in control liquid medium before transferring to: (a) Zn-deficiency for 4 days; (b) Zn-replete conditions (4 days on Zn-deficiency followed by 2 days on control medium); (c) Zn excess (50 μM) for 1 day. The following plant parts were included into analysis: (i) leaves (blades of the two lower leaves counting from the base without petioles and the major midribs); (ii) apical and basal parts of the roots (3–4 cm of the apical part and 3–4 cm of the basal one).

To investigate whether *NtZIP4A* and *NtZIP4B* contribute to Zn homeostasis specifically in leaves under Zn excess, 5.5-week old plants were transferred for 3 weeks to 50 μM Zn (the nutrient solution was renewed every second day). At the end of the experiment leaf blades without the major midribs from two lower leaves (counting from the base) and two upper leaves (counting from the top) were collected separately for expression analysis.

To determine if expression of *NtZIP4A* and *NtZIP4B* is modulated by Cd, 5.5-week old plants were grown in the presence of 4 μM Cd for 3 days. For expression analysis apical and basal part of the roots and leaves (blades of the two lower leaves counting from the base without petioles and the major midribs) were collected.

#### Growth of Plants for Determination of Zn Concentration

To determine the Zn distribution between the roots and shoots in tobacco plants grown at Zn deficit and Zn excess, 5.5-weeks (3 weeks on plates followed by 2.5 weeks on hydroponics on control medium) were exposed to Zn deficit (no Zn added to the control medium) for 4 days, and to Zn excess (50 μM Zn) for 1 day. At the end of the experiment, leaves and roots were separated. Roots were washed with Milli-Q water, then with 5 mM CaCl_2_ at 4°C for 15 min, and again with water (Barabasz et al., 2012). Collected plant parts were dried for 4 days at 55°C in an oven until constant biomass then used for determination of Zn concentration.

#### Growth of Plants for GUS Analysis

Three-week-old transgenic plants expressing *NtZIP4B_p_*::*GUS* and wild-type plants were transferred to liquid control medium for 6 days. Subsequently they were grown in medium lacking Zn, and in parallel in control medium for 4 days, and then used to determine GUS activity. The medium was renewed every 3 days.

### Expression Analysis

Total RNA was extracted from frozen tissue samples with an Plant RNA Mini Kit (Syngen, #SY341010) according to the manufacturer’s recommendations. The yield and quality of DNAse (Invitrogen, #18068015)-treated RNA were determined by NanoDrop spectrophotometer ND 100 (NanoDrop, Wilmington, DE, United States), and 1% agarose gel containing EtBr electrophoresis.

The expression level of *NtZIP* transcripts was determined by quantitative Real-Time PCR (qRT-PCR). Primer sequences are given in the Supplementary File S1. Analysis was performed according to Kendziorek et al. (2016) with minor modifications. The cDNA was synthesized using 0.1–1 μg of RNA and oligo d(T)18 primers with the use of RevertAid^TM^ First Strand cDNA Synthesis Kits (Thermo Scientific) in a 20 μl reaction volume following the manufacturer’s protocol. qRT-PCR was conducted in a Roche Mastercycler (LightCycler^®^ 480 System, Roche) using LightCycler 480 SYBR Green (Master 0488735001) according to the manufacturer’s recommendations. The primer sequences were designed with IDT OligoAnalyzer 3.1^3^ and OligoCalc: Oligonucleotide Properties Calculator^4^, and synthesized by Genomed (Poland). The comparative ΔCt (threshold cycle) method was used to calculate the relative quantities of each transcript in the samples (Livak and Schmittgen, 2001). Validation experiments were performed to test the efficiency of the target amplification and the efficiency of the reference amplification. The general quality assessment of the qRT-PCR results was based on the amplification and melting curve profile of the samples in relation to the assay controls (non-template controls). The expression of each *ZIP* gene in various samples was normalized with tobacco *NtPP2A* (*protein phosphatase 2A;* AJ007496) gene as an internal control. Its stability in the plant samples collected for expression analysis was measured and shown in Supplementary File S2. Expression analysis was performed with at least three independent biological replicates. For each sample, reactions were set up in triplicate and means were calculated.

### Determination of Zn Concentration

Dried plant samples were acid digested in 65% HNO_3_ and 39% H_2_O_2_ (9:1, v:v) in a closed system microwave mineralizer (Milestone Ethos 900, Milestone, Bergamo, Italy). Zinc concentrations were determined by flame atomic absorption spectrophotometry (FAAS) (TJA Solution Solar M, Thermo Electron Manufacturer Ltd., Cambridge, Great Britain). Certified reference material (Virginia tobacco leaves CTA-VTL-2; Commission for Trace Analysis of the Committee for Analytical Chemistry PAS and Institute of Nuclear Chemistry and Technology, Warsaw) was included in each analysis run (Wojas et al., 2009).

### Determination of Tissue-Specific Expression of *NtZIP4B_p_::GUS* in Tobacco

#### Promoter Isolation, Generation of Construct and Plant Transformation

The promoter sequence of *NtZIP4B* (2160 bp upstream of the start codon) was amplified from genomic DNA using appropriate primers (Supplementary File S3) and cloned into pENTR/D-TOPO using pENTR Directional TOPO Cloning Kit (Invitrogen) according to manufacturer’s instructions. One Shot TOP10 *E. coli* were used and the resulting pENTR/D::*ZIP4B*_p_ confirmed by sequencing (Promega, Poland). This was recombined using LR clonase with pMDC163 which links the promoter to the *uidA* gene creating the pMDC163::*NtZIP4B*_p_::*GUS* construct; this was sequenced to confirm correct insertion.

pMDC163::*NtZIP4B*_p_::*GUS* was incorporated into the tobacco genome using the standard procedure of *Agrobacterium tumefaciens-*mediated transformation of tobacco leaf disks as described in Barabasz et al. (2010). Transgenic plants were selected for hygromycin resistance. The T1 generation of selected plant lines with a segregation ratio of 3:1 (tolerant:sensitive) were used to obtain homozygous T2 lines for GUS analysis. Eight independent homozygous lines were chosen for GUS staining.

#### GUS Assay

*NtZIP4B*::*GUS* plants were fixed in 90% ice cold acetone for 25 min with low rotation, then washed four times in the reaction buffer (50 mM Na_2_HPO_4_, pH 7.0, and 0.2% Triton X-100) (the third washing with infiltration). Afterward, samples were transferred to the reaction buffer containing 2 mM X-Gluc (5-bromo-4-choloro-3-indolyl β-d-glucuronic acid), infiltrated for 15 min, then at 37°C in dark for 2.5 h with gentle shaking. At the end samples were cleared with the increasing concentrations of ethanol (50, 70, and 95%) before microscopic analysis (OPTA-TECH microscope).

### Construction of Yeast Expression Vector and Yeast Complementation Assay

pENTR/D-TOPO-*NtZIP4B-*STOP construct was recombined with pAG426GAL-cccB-EGFP (REF) using LR clonase. This tags *NtZIP4B* with GFP at the C-terminus and uses the galactose inducible promoter for expression in yeast. Resulting pAG426-NtZIP4B-STOP and the empty vector pAG426GAL were transformed into yeast using the lithium acetate method (Gietz and Schiestl, 2007). The following *Saccharomyces cerevisiae* strains were used: wild-type DY1457 (MATa, ade1 can1 his3 leu2 trp1 ura3); and *zhy3* mutant; –*zrt1/zrt2* (DY1457 + zrt1::LEU2, zrt2::HIS3) defective in high and low affinity zinc uptake.

Yeast was grown on liquid synthetic complete medium SC-URA containing yeast nitrogen base (with 0.2 mM Zn) supplemented with amino acids (without uracil) and 2% (w/v) glucose; the pH of the medium was adjusted to 5.3 with 1 M potassium hydroxide, and yeast was incubated overnight at 30°C with shaking. The next day, yeast were centrifuged, washed twice and suspended in SC-URA medium containing galactose. The OD_600_ was adjusted to 0.2 and yeast were grown for another 4 h. The OD_600_ was adjusted again to 0.2, and a series of dilutions were prepared (1.0, 0.1, 0.01, and 0.001). Then 5 μl aliquots of each yeast culture were spotted onto plates containing SC-URA medium with galactose (GAL) solidified with 2% (w/v) agar, and supplemented with the required components (details in the Figure legend). Yeast growth was monitored for the next 3–7 days.

To determine whether Zn could be a substrate for NtZIP4, the *zrt1zrt2Δ* yeast strain with the expression of pAG426-*NtZIP4B*-STOP, as well as WT (DY1457) with the expression of empty pAG426GAL were grown on a SC-URA medium with galactose (containing 0.2 mM Zn). The suspension of yeast cultures were spotted onto the plates containing agar-solidified SC-URA with GAL supplemented with 0.5 and 1.5 mM EGTA [ethylene glycol-bis(β-aminoethyl ether)-N,N,N′,N′-tetraacetic acid].

To determine whether Cd is a substrate for NtZIP4, sensitivity to Cd was compared between wild-type yeast (DY1457) expressing the empty vector pAG426GAL and expressing the construct pAG426-*NtZIP4B* STOP. Yeasts were grown on an agar-solidified SC-URA with GAL medium containing 10 and 50 μM Cd (CdCl_2_), and their growth was monitored.

### Construction of GFP-*NtZIP4B* Vector and Subcellular Localization of NtZIP4B

Bioinformatics analysis was performed to predict the subcellular localizations of NtZIP4B protein with the use of the ProtComp v. 9.0 online; http://www.softberry.com/berry.phtml?topic=protcomppl&group=programs&subgroup=proloc.

The subcellular localization was also examined by transient expression of the GFP-NtZIP4B fusion protein in the lower epidermis of tobacco leaves. The entry vector pENTR/D-TOPO-*NtZIP4B-*STOP was recombined using LR clonase with pMDC43 (N-terminal GFP) (Curtis and Grossniklaus, 2003) to generate NtZIP4B linked at the N-terminus to GFP. The resulting pMDC43*-GFP-NtZIP4B-*STOP was sequenced (Genomed, Poland), transformed into *Agrobacterium tumefaciens* GV3101 (C58C1, Rif^R^; pMP90, Gm^R^) and used to inoculate tobacco epidermal cells (Sparkes et al., 2006) to determine the subcellular localization of NtZIP4B.

Transient expression in tobacco was performed as described previously (Wojas et al., 2010; Papierniak et al., 2018). Leaves were monitored under an inverted Leica TCS SP2 confocal microscope (Leica Microsystems Inc., Wetzlar, Germany). GFP signals (argon laser excitation 488 nm) were recorded between 500 and 560 nm. Propidium iodide (50 μM water solution was used to visualize cell walls, excitation 543 nm/emission 617 nM) (Suh et al., 2007; McFarlane et al., 2010). In parallel, chlorophyll autofluorescence was monitored using a HeNe (543 nm) laser for excitation.

### Statistical Analysis

All presented data are from one experiment that is representative of three to four independent replicate experiments. Statistical significance was evaluated at the 0.05 probability level using Student’s *t*-test.

## Results

### Cloning and Sequence Analysis of *NtZIP4A* and *NtZIP4B* Genes

The partial sequence of *NtZIP4* (JZ875395.1) that we identified previously (Barabasz et al., 2016), initially named T-XIII-K12, was used for identification of the full sequence of *NtZIP4* from tobacco. Two cDNA sequences XM_016647965.1 and XM_016586154.1 were retrieved from the NCBI data base with the highest homology to the query sequence (99 and 97%, respectively), and were named *NtZIP4A* and *NtZIP4B* (Supplementary File S1). Specific primers for amplification and cloning of *NtZIP4B* were designed from the consensus sequence of XM_016647965.1. Comparison of the nucleotide sequences of the query sequence, JZ875395.1 and ORF of *NtZIP4A* and *NtZIP4B* is given in the Supplementary File S1.

There is a high homology (97.57%) between the cDNA of identified *NtZIP4A* and *NtZIP4B* genes. Analysis showed that the genomic sequence of *NtZIP4A* and *NtZIP4B* consists of 3436 and 3171 bp, respectively, with 94.62% identity. They have four exons of the same length whereas introns are substantially reduced in *NtZIP4B* compared to *NtZIP4A* (Supplementary File S4). Both *NtZIP4A/B* contain an ORF of 1236 bp, encoding a predicted protein of 412 aa. The sequence of *AtZIP4A* has 96.52% identity at the nucleotide level and 97.57% amino acids (aa) with *NtZIP4B*. Comparison of the aa sequence shows that NtZIP4A/B share the highest homology with ZIP4 transporters from other tobacco species [*N. tomentosiformis, N. sylvestris*, and *N. attenuata* (97–100%), and much lower with *A. thaliana* (67%) and ZIP4 proteins from other plant species (Supplementary File S5)].

**FIGURE 1:**
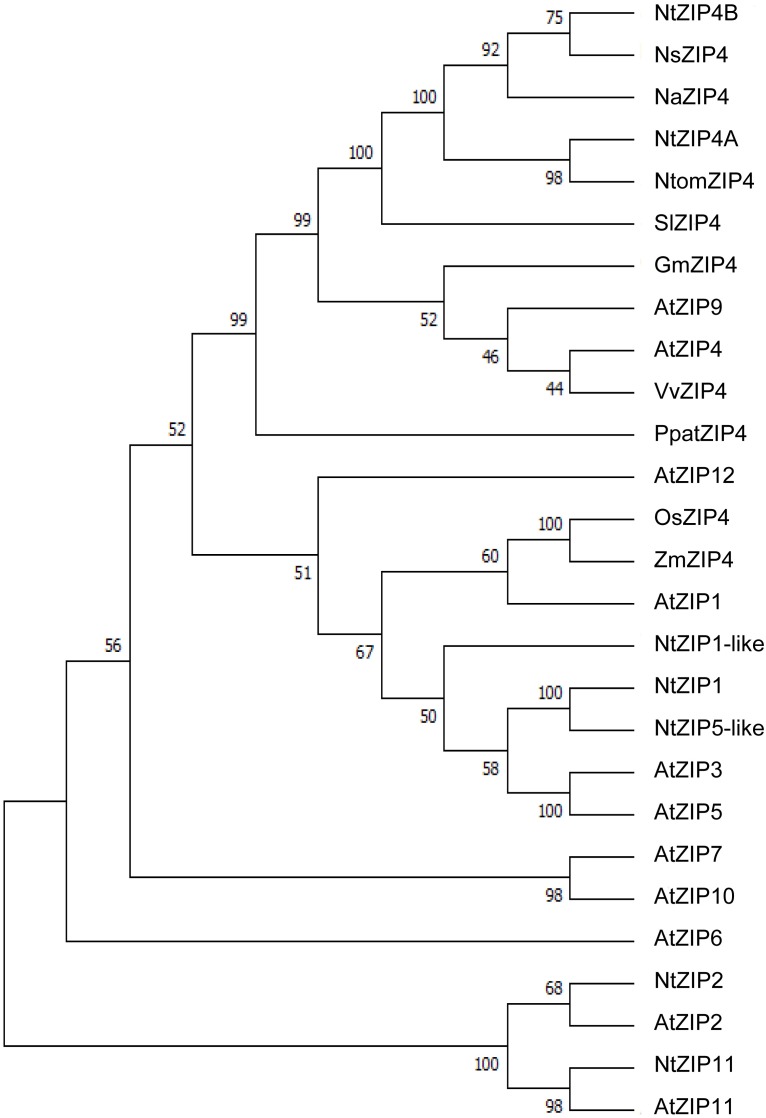
Phylogenetic analysis of ZIP4 transporters from selected species. Unrooted phylogenetic tree for the ZIP proteins from eleven species: At, *Arabidopsis thaliana*; Gm, *Glycine max*; Na, *Nicotiana attenuata*; Ns, *Nicotiana sylvestris*; Nt, *Nicotiana* *tabacum*; Ntom, *Nicotiana tomentosiformis*; Os, *Oryza sativa*; Ppat, *Physcomitrella patens*; Sl, *Solanum lycopersicum*; Vv, *Vitis vinifera*; Zm, *Zea mays*. It was constructed based on amino acid sequences identified in NCBI database, using MEGA 7.0 software. The length of branches are proportional to the degree of divergence. Numbers in the figure represent bootstrap values (1000 replicates). The accession numbers are as follows: AtZIP1 – NP_187881.1, AtZIP2 – NP_200760.1, AtZIP3 – NP_180786.1, AtZIP4 – NP_001320672.1, AtZIP5 – NP_172022.1, AtZIP6 – NP_180569.1, AtZIP7 – NP_178488.1, AtZIP9 – NP_001329099.1, AtZIP10 – NP_174411.2, AtZIP11 – NP_564703.1, AtZIP12 – NP_201022.1, GmZIP4 – XP_014624832.1, NaZIP4 – XP_019247882.1, NsZIP4 – XP_009762749.1, NtZIP1 – NP_001312674.1/BAH66920.1, NtZIP1-like – XP_016507999.1, NtZIP5-like – XP_016449488.1, NtZIP11 – XP_016500060.1, NtomZIP4 – XP_009615465.1, OsZIP4 – XP_015650399.1/BAD18967.1, PpatZIP4 – XP_001754592.1, SlZIP4 – XP_004245100.1, VvZIP4 – XP_010648355.1, ZmZIP4 – NP_001307473.1.

**FIGURE 2:**
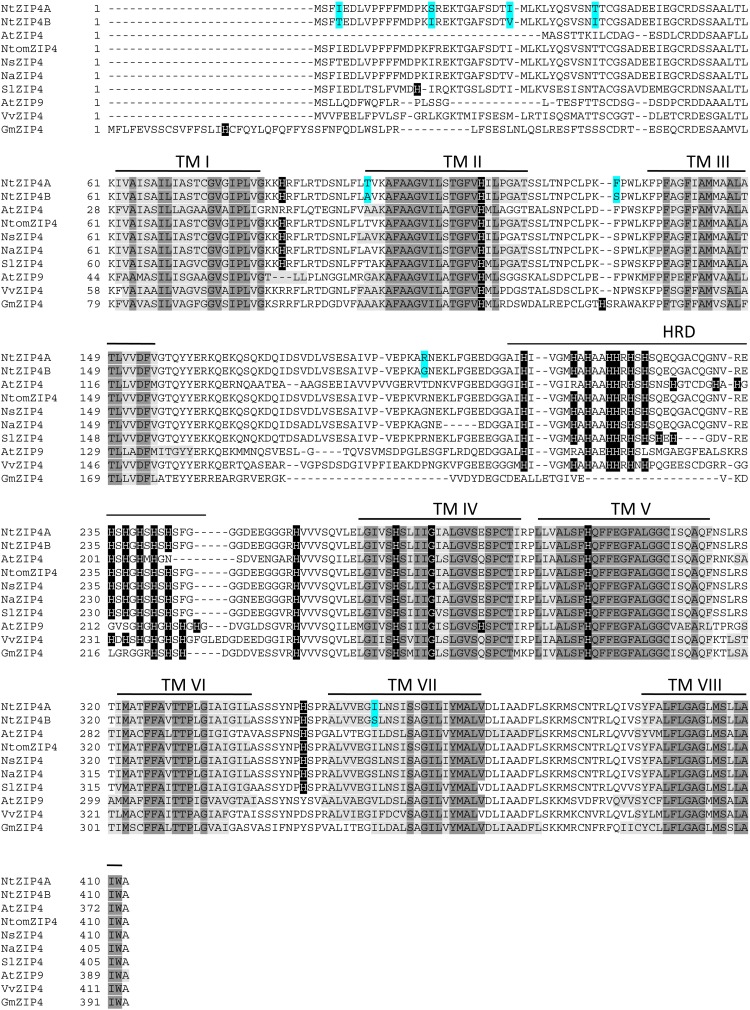
Amino acid alignment of predicted ZIP4 proteins from different species. Sequences were aligned using ClustalW. The prediction of membrane-spanning regions was performed using TMpred program (https://embnet.vital-it.ch/software/TMPRED_form.html), and indicated as lines above the sequences, and numbered I–VIII, respectively. Sequences of the transmembrane domains are marked with light gray; identical amino acids with dark gray; histidines in black. HRD, Histidine Rich Domain within the variable cytosolic region. Dashes indicate gaps.

The phylogenetic tree was constructed by comparing amino acid sequences of both newly identified NtZIP4A and NtZIP4B with 10 known ZIP4 from different plants (including three tobacco species), and 15 other ZIP family members from *A. thaliana* and tobacco (Figure 1). It was shown that tobacco NtZIP4A and NtZIP4B belong to the same clade formed by ZIP4 proteins from other plants such as *A. thaliana*, *G. max*, *V. vinifera*, *S. lycopersicum, Physcomitrella patens* and three tobacco species. Only two ZIP4 from Monocotyledonous plants (OsZIP4 and ZmZIP4) comprised a distinct sub-clade with AtZIP1. The alignment of protein sequences of NtZIP4A and NtZIP4B with ZIP4 proteins from organisms belonging to the same clade (Figure 2) showed high sequence conservation between them. NtZIP4A and NtZIP4B have typical characteristics of the ZIP family members, containing eight transmembrane (TM) domains, very long N-terminal tails and short C-terminal region both located on the external surface of the plasma membrane. Between TM domains III and IV there is a long variable cytosolic region containing multiple histidine residues. considered as a potential metal binding motif (HRD – Histidine Rich Domain). The ZIP4A/B protein contain also in the IV TM the ZIP signature region with the most conserved consensus sequence being [L I V F A] [G A S] [L I V M D] [L I V S C G] [L I V F A S] H [S A N] [L I V F A] [L I V F M A T] [L I V D E] G [L I V F] [S A N] [L I V F] [G S] (Eng et al., 1998).

### NtZIP4B Transports Zn and Cd

**FIGURE 3:**
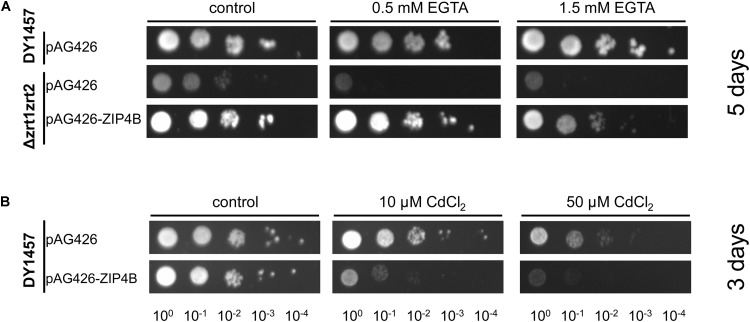
Complementation by *NtZIP4B* cDNA of yeast mutants defective in metal uptake on selective media. Yeast cells: DY1457, *Δzrt1zrt2* (defective in Zn uptake) were transformed with empty vectors pAG426 as a control, or with vectors carrying tobacco gene *NtZIP4B* with the stop codon pAG426-ZIP4B-STOP (pAG426-ZIP4B). Yeast cultures were adjusted to an OD600 of 0.2 and 5 μl of serial dilutions (from left to right in each panel) was spotted on SC-URA medium containing 2% (w/v) galactose solidified with 2% agar, supplemented with EGTA **(A)**, CdCl_2_ **(B)** or 0.2 mM Zn (control). The plates were incubated for 3–6 days at 30°C. The images are representative of three independent experiments taken after 5 days of growth **(A)** and 3 days of growth **(B)**.

Yeast complementation assays were conducted to determine whether NtZIP4B has the capacity to transport Zn and Cd. To investigate the role of NtZIP4B as a Zn uptake transporter, the yeast double mutant Δ*zrt1zrt2* (ZHY3) defective in high and low affinity of Zn uptake (Eide, 2003) was used to test for complementation. The growth defect of this mutant was markedly reversed by expression of pAG426-*NtZIP4B* STOP (Figure 3A).

To test the Cd transport activity, the construct and the empty vector pAG426 were expressed in the wild type DY1457 strain, and yeast were exposed to 10 and 50 μM Cd. Increased sensitivity of yeast transformed with pAG426-*NtZIP4B* STOP suggests that the presence of NtZIP4B protein likely mediates Cd influx which contributes to increased sensitivity of yeast to Cd (Figure 3B).

### Developmental Regulation of Tobacco ZIPs

**FIGURE 4:**
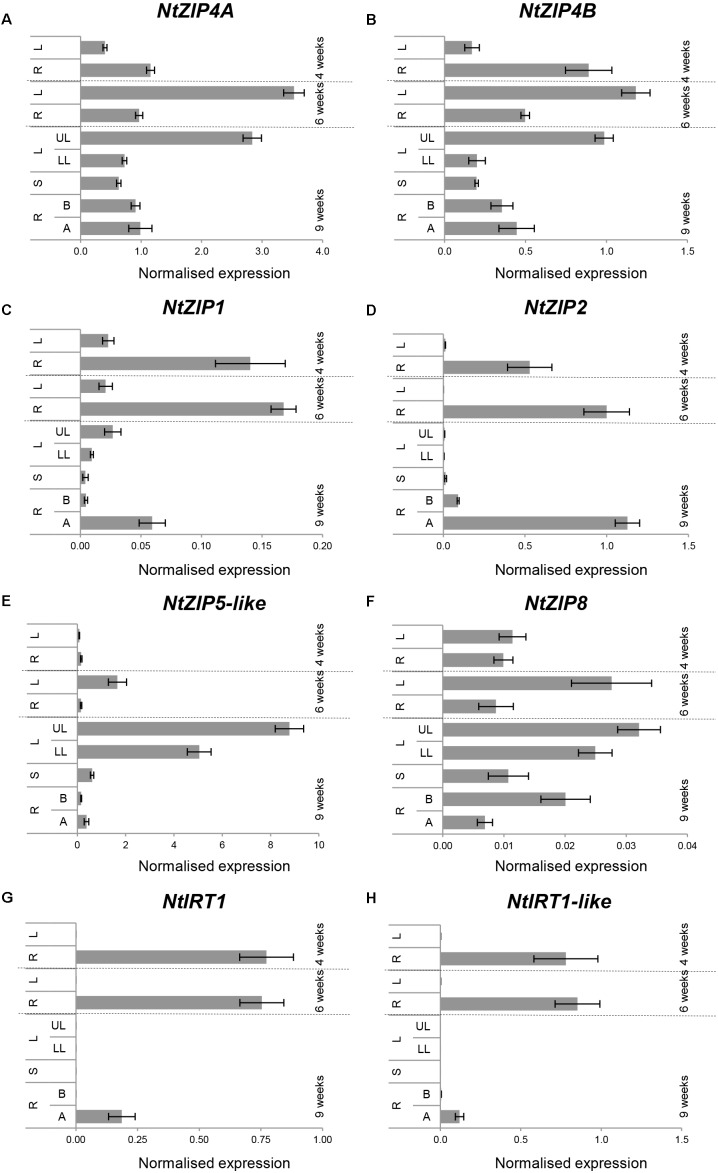
Organ- and developmental-regulation expression of selected *ZIP* genes in *N. tabacum* **(A–H)**. Analysis was performed by RT-qPCR. Plants were grown at control medium. Transcript levels were monitored in the whole roots and leaves of plants grown for 4 and 6 weeks, and after 9 weeks in the apical (A) and basal (B) segments of roots (R), in stems (S), in young upper leaves (UL), and old lower leaves (LL). The level of genes transcript was normalized to *PP2A* expression level. Values correspond to means ± SD (*n* = 3); those with the ratio greater than two are considered significantly different.

Tests were performed on 2-; 6- and 9-week old plants grown under control conditions. The expression pattern of *NtZIP4A* and *NtZIP4B* at all three stages of vegetative development was similar, with moderately higher expression levels of *NtZIP4A* (Figures 4A,B). In young 4-week old seedlings, the transcript abundance was significantly higher in roots. With time this pattern changes and the expression increases in leaves – especially in the upper, older ones. The transcript level in the roots remains at similar moderate level in the apical and basal segment.

Analysis performed for other *ZIPs* from tobacco identified *NtZIP1, NtZIP2*, *NtIRT1*, and *NtIRT1-like* as those with the root as a major site of expression, primarily in the apical segments (Figures 4C,D,G,H). In contrast, only *NtZIP5*-like was specifically expressed in leaves and the expression levels increased with the age of the plants and also with age of leaves (higher in the upper ones) (Figure 4E). Out of seven tested *NtZIP* genes, the transcript abundance of *NtZIP8* was very low at all developmental stages, however, it was detectable in all organs with higher level in leaves (Figure 4F).

### Regulation of Tobacco *ZIPs* by Zn and Cd

#### Zn- and Cd-Dependent Expression of NtZIP4A and NtZIP4B

**FIGURE 5:**
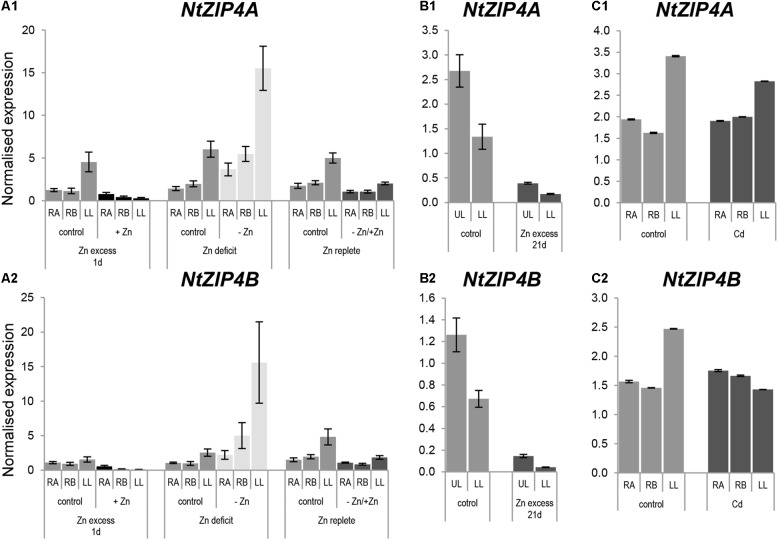
Expression pattern of *NtZIP4A* and *NtZIP4B* in *N. tabacum* under various Zn conditions **(A1,B1,A2,B2)** and in the presence of Cd **(C1,C2)**. Plants were grown in the quarter-strength Knop’s medium (control) and then transferred to the control medium supplemented with the following Zn or Cd concentrations: (i) 50 μM Zn for 1 day (Zn excess-1d); (ii) without Zn for 4 days (Zn deficit); (iii) plants grown at Zn-deficiency for 4 days were transferred to the control medium for 2 days (Zn replete); (iv) 50 μM Zn for 21 days (Zn excess-21d); (v) 4 μM Cd for 3 days (Cd). RT-qPCR analyses was performed on cDNA prepared from the two old lower leaves counting from the base (LL), two young upper leaves (UL), apical part of roots (RA), and basal part of roots (RB) of *N. tabacum*. Gene expression was normalized to the *PP2A* level. Values correspond to means ± SD (*n* = 3); those with the ratio greater than two are considered significantly different.

Knowing that Zn is a substrate for NtZIP4B, expression analysis was performed to determine whether its expression pattern depends on the Zn status. There is a high similarity of the expression pattern of *NtZIP4A* and *NtZIP4B* isoforms (Figure 5) distinguished by the use of primer sets indicated in the Supplementary File S1. In 6.5-week old plants grown at control conditions and Zn-deficiency expression of both *NtZIP4A* and *NtZIP4B* was significantly higher in leaves than in roots (Figures 5A1,A2). The transcript abundance increased at Zn-deficiency in all studied organs (apical and basal segments of the roots and in leaves), and upon resupply of the control medium it was reduced. Also, downregulation was detected upon exposure for 1 day to high 50 μM Zn. Similarly, in older 8.5-week old plants exposed to 50 μM Zn for 21 days, expression level of both *NtZIP4A* and *NtZIP4B* decreased dramatically with a more pronounced fall in the lower (older) leaves (Figures 5B1,B2).

Having ascertained that NtZIP4B mediates transport of Cd, the possibility of Cd regulation of *NtZIP4A and NtZIP4B* expression was examined. Three-day exposure to Cd did not significantly modify the transcript level in the roots and leaves (Figures 5C1,C2).

#### Zn-Dependent Expression of NtZIP1, NtZIP2, NtZIP5-Like, NtZIP8, NtIRT1, and NtIRT1-Like

**FIGURE 6:**
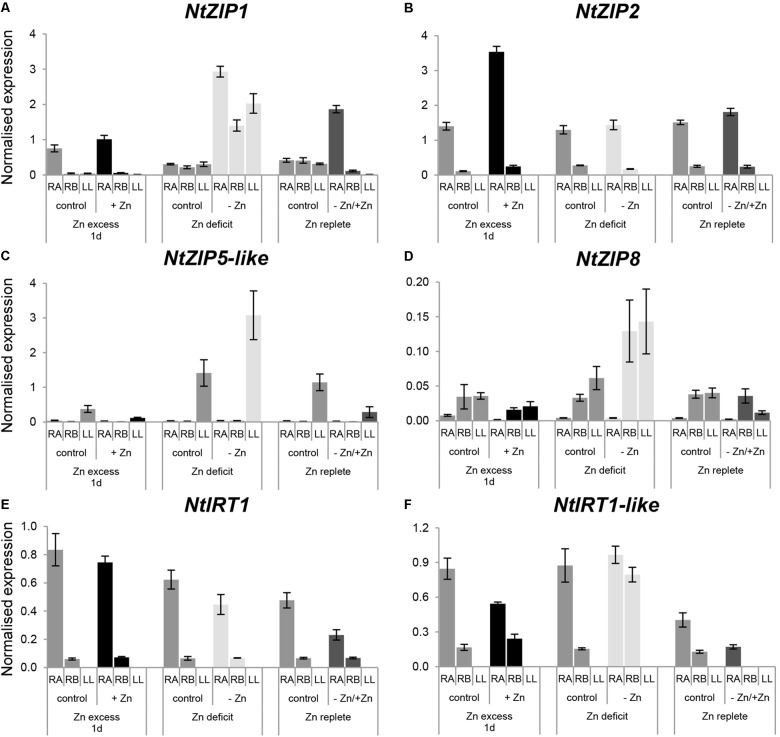
Expression pattern of *NtZIP1* **(A)***, NtZIP2* **(B)**, *NtZIP5* **(C)**, *NtZIP8* **(D)***, NtIRT1* **(E)**, *and NtIRT1-like* **(F)** in *N. tabacum* under various Zn conditions. Plants were grown in the quarter-strength Knop’s medium (control) and then transferred to the control medium supplemented with the following Zn concentrations: (i) 50 μM Zn for 1 day (Zn excess-1d); (ii) without Zn for 4 days (Zn deficit); (iii) plants grown at Zn-deficiency for 4 days were transferred to the control medium for 2 days (Zn replete). RT-qPCR analyses was performed on cDNA prepared from the two old lower leaves counting from the base (LL), two young upper leaves (UL), apical part of roots (RA), and basal part of roots (RB) of *N. tabacum*. Gene expression was normalized to the *PP2A* level. Values correspond to means ± SD (*n* = 3); those with the ratio greater than 2 are considered significantly different.

To learn more about involvement of other *ZIP* genes in Zn homeostasis in tobacco plants, expression levels of *NtZIP1*, *NtZIP2, NtZIP5*-like, *NtZIP8, NtIRT1*, and *NtIRT1*-like at Zn excess, Zn-deficiency and resupply were also determined. In all experiments, to shed more light on the physiological difference between the young apical and old basal segments of the roots, expression of selected *ZIP*s within these two parts was compared (Figure 6).

In roots, under control conditions the expression level in the apical part for *NtZIP1, NtZIP2, NtIRT1*, and *NtIRT1*-like was higher than in the basal region (Figures 6A,B,E,F), whereas for *NtZIP8* (which generally showed low expression levels) it was lower (Figure 6D). The expression level of *NtZIP5*-like did not differ significantly between both root parts (Figure 6C).

When Zn-deficiency was applied, upregulation of *NtZIP8* and *NtIRT1*-like was observed in the basal part of the root only, with no change in the apical region (Figures 6D,F). Upregulation was also noted for *NtZIP1*, however, this was observed in both regions of the root (Figure 6A). Return to control conditions (Zn resupply) resulted in downregulation of those genes which had been up-regulated by Zn-deficiency. In leaves, Zn-limiting conditions enhanced the transcript level of *NtZIP1* (Figure 6A), and to lesser extent *NtZIP5*-like (Figure 6C), and a reduction was observer on resupply conditions.

Exposure for 1 day to 50 μM Zn did not significantly change the transcript level of the *ZIP* genes tested within the basal root segment. However, in the young apical part, transcript abundance of *NtZIP2* increased whereas *NtIRT1*-like decreased. In leaves, expression of the *ZIP*s tested was very low and not modified by Zn excess, except for a reduction of mRNA level of *NtZIP5*-like (Figures 6A–F).

### Accumulation of Zn at Zn Deficit and Zn Excess

**FIGURE 7:**
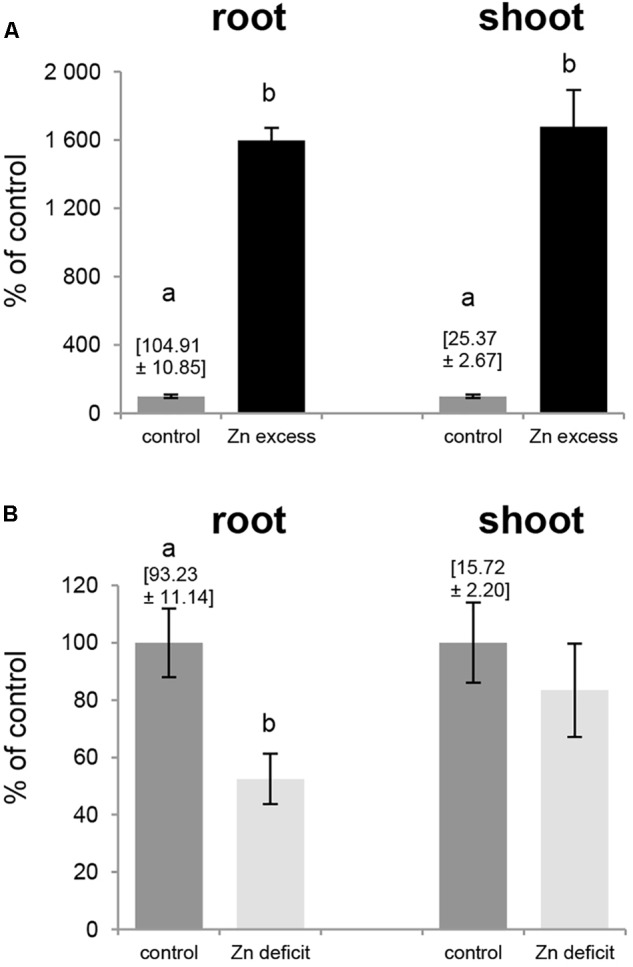
Zinc concentration in tobacco plants. Plants were grown in the quarter-strength Knop’s medium (control) and then transferred to the control medium supplemented with 50 μM Zn for 1 day (Zn excess), or to the medium without Zn for 4 days (Zn deficit). Results are demonstrated as percent of the control values: Zn concentration at control conditions equals 100%, and at Zn excess and Zn deficit is shown as multiple of values for the control ones. Zn concentrations [mg/kg dry weight] at control conditions is given above the control bars. **(A)** Zn excess – 50 μM Zn; **(B)** Zn deficit. Values correspond to means ± SD (*n* = 10). Significant differences between the control plants and plants exposed to a given Zn concentration (Zn excess or Zn deficit) are marked by different letters – evaluated by Student’s *t*-test (*P* ≤ 0.05).

Exposure to Zn deficit (no Zn added to the medium) and Zn excess (50 μM Zn) affected expression pattern of tobacco *ZIP* genes (Figures 5, 6), thus we examined Zn concentration in these plants (Figure 7). The concentration of Zn in the roots and shoots of plants exposed to 50 μM Zn increased by ∼1600% (as compared to the control conditions) although at control conditions it was 4 times higher in roots than in shoots (Figure 7A). In contrast, upon Zn deficit the concentration of Zn in roots decreased by about 50% while in shoots it remained at the level detected at control conditions (Figure 7B) indicating initiation of the mechanism to maintain relatively stable Zn concentration in photosynthesizing organs.

### Localization of *NtZIP4B-GUS* Expression

To analyze the *in planta* function of *NtZIP4B*, the first 3500 bp sequence of genomic DNA upstream of the ATG start codon was identified in the data base and compared to the 3519 bp sequence of *NtZIP4A* (Supplementary File S3). Alignment of both promoter sequences showed that they both contain the *cis* element ZDRE (Zinc Deficiency Response Element) element A**TGTCGACA**T (Assunção et al., 2010) localized at -335 bp for *NtZIP4A* and at -310 bp for *NtZIP4B*. The IDE1 (iron-deficiency-responsive element; ATCAAGCATGCTTCTTGC) and IDE2 (TTGAACGGCAAGTTTCACGCTGTCACT) responsible for Fe-deficiency response (Kobayashi et al., 2003) were not found.

**FIGURE 8:**
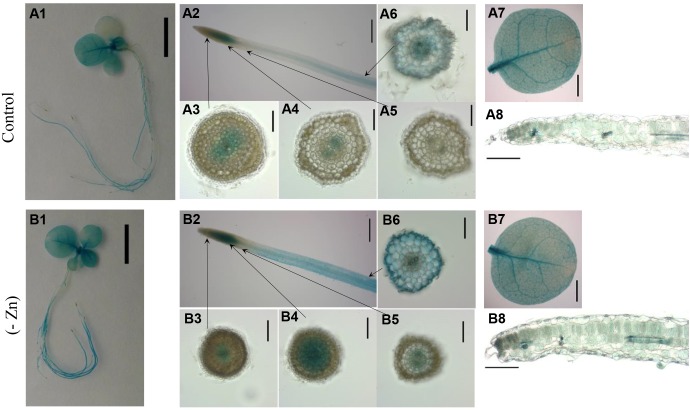
GUS staining pattern of transgenic plants expressing *NtZIPB*p::*GUS* fusion gene. GUS expression in 4-week-old transgenic and wild-type seedlings grown at control conditions **(A1–A8)** and in the Zn-deficient (-Zn) medium **(B1–B8)** for 4 days. Whole seedlings **(A1,B1)**; apical root segments **(A2,B2)**; cross sections through the roots **(A3–A6,B3–B6)**; whole leaves **(A7,B7)** and the cross sections through leaves **(A8,B8)**. Magnification bars: 1 cm – **A1,B1**; 0.5 cm – **A2,B2**; 0.1 cm – **A3–A6,B3–B6**; 0.2 cm – **A7,B7**; 0.2 mm – **A8,B8**.

To analyze the tissue-specific expression of *ZIP4* in tobacco, the 2160 bp upstream of *NtZIP4B* coding sequence was fused with the *uidA* (*GUS*) coding sequence (*NtZIP4B*_prom_::*GUS* reporter construct). The spatial expression pattern in the T2 homozygous generation of transgenic tobacco plants was investigated (Figure 8). In plants grown under Zn-deficiency conditions, blue staining resulting from the activity of the *NtZIP4B* promoter was stronger in the roots and to lesser extent in the leaves, as compared with control conditions (Figures 8A1,A2,A7,B1,B2,B7). In control conditions, in the apical meristematic root part, GUS activity was absent above the quiescent center except the central cylinder (procambium) and adjacent ground meristematic cells which will differentiate into the endodermis (Figures 8A3,A4). Further up in the more mature region where ongoing cellular differentiation is accompanied by significant vacuolization, GUS staining becomes weaker, and ultimately disappears (Figure 8A5). At a distance of approximately 10 mm from the root tip GUS staining was detectable again with gradually increasing intensity, first in the central cylinder then further up across the whole section including the epidermis (Figure 8A6). Such a pattern was characteristic for the middle root part, and again, closer to the root base (root-shoot junction) GUS staining disappeared. In Zn-deficient plants the pattern of *ZIP4B*-driven promoter activity was the same as in control conditions except that in the roots the zone with GUS activity expanded toward the root apex and was more intensive (Figures 8B3–B6).

In the shoot, in plants grown in control medium, GUS staining attributable to *NtZIP4B* promoter activity was present in cotyledons and in younger leaves (Figure 8A1) whereas at Zn-deficiency it was detected in all leaves (Figure 8B1). It was especially active in the veins, but also in areas between them (Figures 8A7,B7), and at the cross sections blue staining was seen in all tissues primarily in the palisade and spongy parenchyma, and to a much lesser extent in the upper and lower epidermis (Figures 8A8,B8), with more intensive blue staining at low Zn (Figure 8B8). GUS activity was not detected in wild-type tobacco (data not shown).

### Plasma Membrane Localization of NtZIP4B

First indication on the subcellular localization of NtZIP4B comes from the bioinformatics analysis. Based on the ProtComp program the protein of NtZIP4B is targeted to the plasma membrane (Supplementary File S6).

**FIGURE 9:**
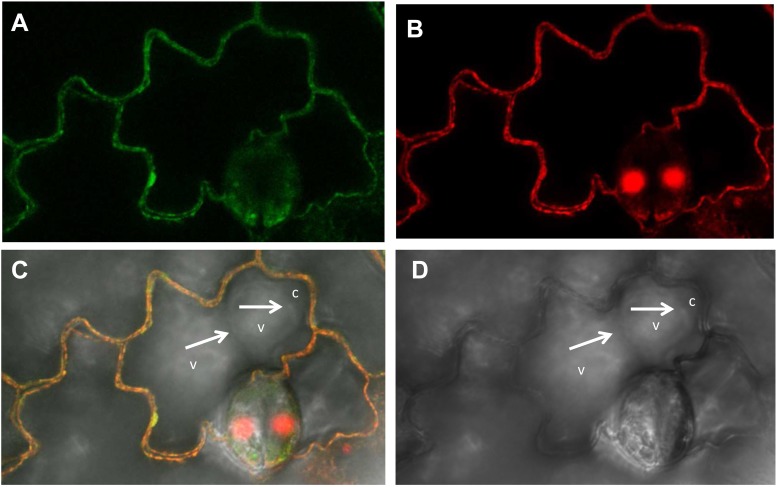
Plasma membrane localization of the NtZIP4B-GFP fusion protein transiently expressed in the lower epidermis of tobacco leaf. Laser scanning confocal micrographs include: **(A)** GFP fluorescence concentrated to the cell’s contours, autofluorescence of the cell wall of non-transformed cells is below the detection limit for settings used for visualization of the GFP signal; **(B)** propidium iodide red fluorescence of the cell’s contours; **(C)** overlapped GFP and propidium iodide signal – both signals with the same localization; **(D)** bright field. **(C,D)** White arrows indicate localization of the tonoplast at the border between two vacuoles (v), and at the border between the vacuole and the cytoplasm (c) pushed into a protruding end of a cell. Green and red fluorescence is not present in the tonoplast (at the border between the vacuoles, and between the vacuole and the cytoplasm within the protruding end of a cell).

Next, to determine the subcellular localization of NtZIP4B, the ORF of *NtZIP4B* cDNA, N-terminally fused with green fluorescent protein (*GFP*) expressed under cauliflower mosaic virus 35S promoter, was transiently expressed in tobacco leaves. Green fluorescence in the epidermal cells resulting from the expression of pMDC43-*GFP-NtZIP4B*-STOP became first detectable after 3 days from the infiltration of tobacco leaves with the suspension of *A. tumefaciens*, and lasted for approximately 20 h. The use of tobacco lower epidermal cells for transient expression is ideal for discrimination of the localization of a target protein between the plasma membrane and the tonoplast due to irregular shapes of those cells. They contain one or two large central vacuole/s which does/do not enter the narrow protruding ends (Figure 9D). In such cells, within each protruding end the cytoplasm is usually pushed into the corner by the vacuole, therefore within these regions the tonoplast and the plasma membrane are clearly spatially separated (Figure 9D). In this study the green signal has not been detected in these sites. It was also not present at the border between two adjacent central vacuoles (Figures 9C,D). Here, it followed the contours of epidermal cells and overlapped with the red signal from the cell walls stained with propidium iodide (Figures 9A–C). Since it is not possible to discriminate between the primary cell wall and the plasma membrane under the confocal light microscope, propidium iodide is used as indication of the localization of the adjacent plasma membrane (Pighin et al., 2004; Lee et al., 2010; Siemianowski et al., 2013; Papierniak et al., 2018). Therefore, co-localization of the GFP-derived signal and the red signal from the cell wall stained with the propidium iodide indicates localization of GFP-fused NtZIP4B protein at the plasma membrane.

## Discussion

In a search for candidate metal transport genes involved in metal homeostasis in tobacco, the putative *NtZIP4* sequence homologous to EST no JZ875395.1 has been isolated (Barabasz et al., 2016). Here, we identified two copies of tobacco *NtZIP4* (*NtZIP4A* and *NtZIP4B*), and *NtZIP4B* has been cloned and characterized. It is likely that both *NtZIP4* transporters derive from two ancestors (*Nicotiana sylvestris* and *Nicotiana tomentosiformis*) of *N. tabacum*. It is an allotetraploid (2n = 4× = 48) species, therefore genes are present generally in two copies (Kenton et al., 1993; Clarkson et al., 2005). At the amino acid level NtZIP4A shares 99.51% homology to NtomZIP4 from *Nicotiana tomentosiformis*, whereas NtZIP4B – 100% homology to NsZIP4 from *N. sylvestris;* thus it seems likely that each of them originated from one of these parents. The NtZIP4A and NtZIP5B share 97.57% homology at the protein level (Supplementary File S5), and they differ in eight amino acids (Figure 2). However, these amino acids are not located within regions known to be crucial for transport and substrate specificity such as the variable cytoplasmic region rich in cysteine residues or transmembrane domains I-VI (Eng et al., 1998). Both NtZIP4A/B formed a distinct clade with ZIP4 proteins from other plant species (Figure 1); which indicate high sequence conservation between them. Moreover, they share all typical characteristics of the ZIP family including the eight transmembrane domains (TM) with a long N-terminal and short C-tail, the variable region containing histidine residues, and the conserved ZIP signature region within the TM IV (Figure 2).

The question is whether *NtZIP4A* and *NtZIP4B* play the same role in *N. tabacum*. Expression pattern of both *NtZIP4A* and *NtZIP4B* in plants grown on medium containing low-to-high Zn concentrations was qualitatively similar (Figure 5). Upregulation at Zn-limiting conditions and downregulation upon resupply of control medium as well as upon Zn excess (50 μM Zn) indicate a function in coping with Zn deficiency. Both *NtZIP4A* and *NtZIP4B* have ZDREs, Zinc Deficiency Response Elements in their promoters (Supplementary File S3), which is consistent with the importance of this element for up-regulation under Zn deficiency (Assunção et al., 2010; Evens et al., 2017; Nazri et al., 2017). Results suggest that these novel tobacco *NtZIP4A* and *NtZIP4B* genes likely have a similar function. Similarly, a high degree of functional redundancy was found between *NtHMA*α and NtHMAβ (the only orthologs for *AtHMA2* and *AtHMA4* in the tobacco genome; Hermand et al., 2014), which in tobacco shared partially overlapping functions in their involvement in Zn and Cd root-to-shoot translocation and distribution throughout the plant body. Two copies of *MTP1* (Metal Tolerant Proteins 1) were also identified and cloned from *N. tabacum* but only one from *N. glauca* (Shingu et al., 2005) with high homology level (95%) between amino acid sequences for all three of them. Similar regulation of *NtMTP1A* and *NtMTP1B* expression as well as their ability to complement the yeast Zn-dependent mutant led to the conclusion that they have the same role in tobacco.

Performed experiments indicate that Zn is a substrate for NtZIP4B, as it suppressed the growth defect of *zrt1zrt2* Zn uptake yeast mutant (Figure 3). Similarly, it was shown that ZIP4 from rice and maize mediate translocation of Zn (Ishimaru et al., 2005; Li et al., 2013). Furthermore, our study indicates that NtZIP4B mediates transport of Cd. Its expression in the wild-type strain rendered yeast more sensitive to this toxic metal (Figure 3). It is known that ZIP proteins are able to transport various divalent cations including Zn, Mn, Fe, Ni, Co and also Cd. For example Cd as one of the substrates was shown for OsZIP1 and CsZIP1 (Ramesh et al., 2003; Mizuno et al., 2008), AtZIP3 (Grotz et al., 1998), MtZIP5 (Stephens et al., 2011), and AtIRT1 (Rogers et al., 2000). NtZIP4B is the first ZIP4 protein for which ability to transport Cd was demonstrated. Despite this, the expression of *NtZIP4A/B* did not respond to 3-day exposure to 4 μM Cd (Figures 5C1,C2). There are, however, Cd-inducible *ZIP* genes such as *AtZIP4*, *AtZIP12*, or *NtIRT1* indicating either transcriptional regulation or secondary effects due to changes in the status of other nutrients, for example Zn or Fe (Hodoshima et al., 2007; Barabasz et al., 2010, 2012; Jain et al., 2013). In conclusion, results indicate that NtZIP4B is a functional Zn and Cd transporter in tobacco. Furthermore, bioinformatic analysis (Supplementary File S6) and transient expression of pMDC43-*GFP-NtZIP4B*-STOP in tobacco leaves (Figure 9) pointed to a plasma membrane as a target membrane within a cell, which suggest a function for NtZIP4B as an uptake protein. Similarly, the plasma membrane localization of ZIP4 protein has been reported for *Oryza sativa* (Ishimaru et al., 2005) and for *Morus notabilis* (Fan et al., 2018).

Taking into account the same expression pattern of both *NtZIP4A* and *NtZIP4B* during development and as a response to low/high Zn in the medium (Figures 4, 5), it seems likely that both genes encode transporters of similar properties important for Zn nutrition/supply in the roots and leaves. Higher expression is detected in leaves (especially in the upper young developing ones) compared to roots, indicating a contribution of *NtZIP4A/B* to maintaining mineral homeostasis throughout the plant body with a specific role in photosynthesizing organs. At the tissue level, its expression in leaves examined by *NtZIP4B* promoter derived GUS activity was localized to the vasculature, and also to the mesophyll cells (Figure 8) suggesting involvement in the regulation of Zn unloading from vessels and in providing Zn to other leaf cells. The ZIP4-dependent uptake of Zn from the apoplast seems to be intensified at low Zn and extended to cover all leaves (Figures 8A1,B1). Moreover, as a Cd uptake protein, NtZIP4 might also participate in accumulation of this toxic metal in leaves.

In roots, examination of the cellular localization of the *NtZIP4B* promoter dependent GUS activity showed blue staining in all tissues across the section through the middle root part, including epidermis (Figures 8A2,B2). This indicates that NtZIP4B, in addition to uptake of Zn from the soil solution, also provides cells of the internal tissues with this microelement. Noteworthy, its presence in the middle root segment points to importance of that region for Zn absorption. Similar territory of expression within roots was shown for Fe and Zn uptake protein AtIRT1 (Vert et al., 2002), but for AtIRT2 detected GUS staining in the epidermis was restricted to the subapical root region only (Vert et al., 2001). Knowledge on the role of the apical, middle or basal root segments in the uptake of metals and in regulation of their root-to-shoot translocation is only rudimentary. As expected, different Zn uptake proteins perform their function in different root regions, which probably complement their activity. Lack of the *NtZIP4B* promoter derived GUS staining in the proximal (basal) segment of the root suggests that metal uptake might not be the key function of that root part. NtZIP4B was shown to be important also in providing certain meristematic cells with Zn under control conditions and at Zn deficit. *NtZIP4B_p_::GUS* expression was noted within the root apex in the procambium, primarily in the cells differentiating into phloem and in adjacent internal layer of the ground meristem differentiating into the endodermis (Figures 8A2–A4,B2–B5). It is not known why the regulation of Zn uptake in these meristematic cells is different from the adjacent ones such as protoderm (dermatogen) and outer layers of the ground meristem. This phenomenon, however, drives our attention to the fine-tuned regulation of Zn homeostasis within the meristematic region. It is expected that other Zn uptake protein is active to provide neighboring cells with this key element for plant growth and development. Expression within the quiescent center and the youngest histogens such as ground meristem (periblem), procambium (plerome) but not in the protoderm (dermatogen) were also shown for Zn transporter from rice *OsZIP4* (encoding the plasma membrane protein) with the use of the *in situ* hybridization method (Ishimaru et al., 2005).

Upregulation of *NtZIP4B* by low Zn in the medium detected by Real-time qPCR (Figure 5) and GUS-activity analysis (Figures 8B1–B8) indicate, that it is likely a part of a plant’s strategy in coping with Zn-deficiency. The mechanism was not only to increase the expression level (enhanced intensity of GUS staining) in the roots and leaves, but also to expand the area of expression to younger root tissues (closer to the root apex) (Figures 8B2–B6).

In plants *ZIP* genes have been shown to regulate and also contribute to the uptake and root/shoot distribution of Zn in a variety of species (Palmer and Guerinot, 2009; Sinclair and Krämer, 2012; Ricachenevsky et al., 2015; Giust et al., 2018). To maintain Zn homeostasis under changing nutrient conditions, the concerted action of numerous transport genes is necessary. Here we showed that in tobacco grown under Zn deficiency conditions, the concentration of Zn in the shoots was maintained at the level of control plants whereas in the roots it was significantly lower (Figure 7B). These indicate the existence of mechanisms underlying the regulation of Zn root-to-shoot translocation which provide shoots with the proper Zn amount at the expense of Zn root concentration. In contrast, at Zn excess the increase in Zn concentration was proportional in the roots and shoots (Figure 7A). To integrate the function of *NtZIP4A/B* with the possible functions of other tobacco *ZIP* genes in regulation of Zn homeostasis under Zn deficit and Zn excess, we performed comparative expression analysis of so far poorly characterized *NtZIP1*, *NtZIP2*, *NtZIP4, NtZIP5*-like, *NtZIP8*, *NtIRT1-like*, and *NtIRT1*.

The expression pattern at low-to-high Zn status was not homogenous for tested genes, however, there were some interesting responses indicating complementarity of encoded transporters in coping with the mineral stress. Examined genes grouped into two categories with respect of their primary site of expression – roots and leaves. These indicate necessity for differential regulation of metal transporters in roots as organs responsible for absorption and transfer of nutrients to shoots, and in photosynthesizing organs receiving nutrients.

The first group includes *NtZIP5*-like and *NtZIP8* (and to a lesser extent *NtZIP4A/B*) with leaves as the major site of transcript accumulation. They likely play a dual function in Zn nutrition providing cells with Zn during development at control conditions (Figure 4), but also at low Zn. Their expression was upregulated by low Zn (Figure 6) which might result in more efficient uptake. To compare, high expression in leaves was also reported for *PtZIP5, ZmZIP5*, and *VvZIP5* (Gainza-Cortés et al., 2012; Mondal et al., 2014; Fu et al., 2017), whereas the opposite pattern with higher transcript in roots for *AtZIP5* and *OsZIP5* was noted (Wintz et al., 2003; Wu et al., 2009; Lee et al., 2010; Milner et al., 2013). The *ZIP8* from *V. vinifera* or from *Monocotyledonous* species such as rice or maize were shown to be expressed at a higher level in the roots compared to shoots and upregulated at low Zn (Lee et al., 2010; Gainza-Cortés et al., 2012; Li et al., 2013).

Roots were the major organs of expression for genes from the second group (*NtZIP1*, *NtZIP2*, *NtIRT1*, and *NtIRT1-like*). At control conditions, for all genes the highest transcript level was detected in the apical segments, however, they differed in the regulation by low/high Zn status in the young apical and the older basal root parts (frequently displaying secondary growth). However, it is not clear to what extent different root parts contribute to Zn uptake, accumulation and to the efficiency of Zn long-distance translocation to shoots.

Upregulation in both root segments at low Zn was shown for *NtZIP1* (Figure 6A). To compare, GUS-based analysis of Zn-deficiency inducible *AtZIP1* revealed its expression in the root and shoot vasculature only, suggesting a general role in the regulation of the metal movement to the shoot (Milner et al., 2013). Noteworthy, ZDRE elements were identified in the promoters of those genes upregulated by low Zn, *AtZIP1*, *AtZIP4, AtZIP5* from *A. thaliana*, but not of *AtZIP2* which is not Zn-deficiency inducible (Assunção et al., 2010). Similarly, lack of activation of *NtZIP2* in tobacco by Zn-limiting conditions was detected. On the contrary, its expression level increased in roots in response to high toxic Zn concentration (50 μM), but primarily in the apical segment (Figure 6B). Consequently, *NtZIP2* could contribute to efficient Zn accumulation in roots at Zn excess. It is known that with increasing Zn concentrations its root/shoot partition changes, and the % of the total Zn kept in roots increases (van de Mortel et al., 2006). Sectorial-specific expression in the roots was also shown for *AtZIP1* although the pattern was different. The GUS-based analysis indicated high expression in the root stele increasing in the more mature regions, especially in the root-shoot junction (Milner et al., 2013). Diversity of expression pattern of tested *ZIP* genes in tobacco in different root sectors was also found for *NtIRT1*-like. At low Zn upregulation was seen in the basal root part only. On the other hand, for *NtIRT1* in the apical/basal root part the high/low expression level, respectively, was maintained irrespective of the Zn status (Figure 6F). It is known that *NtIRT1* is inducible by Fe-deficiency but not by Zn-deficiency (Hodoshima et al., 2007) similar to *AtIRT1* or *OsIRT1* (Korshunovas et al., 1999; Connolly et al., 2002; Ishimaru et al., 2006). Enzymatic GUS activity assay showed *AtIRT1* promoter activity predominantly in the epidermis of the whole roots except the meristematic zone (Vert et al., 2002). However, tissue-specific expression pattern of *OsIRT1* depended on the root sector. At Fe-deficiency conditions, in the apical and basal part of the root GUS staining was detected in the epidermis, exodermis and the stele whereas in the middle part expression was seen predominantly in the cortical cells adjacent to the endodermis, but not in the epidermis (Ishimaru et al., 2006).

In summary, the present study provides essential information on the newly cloned tobacco NtZIP4B transporter. It was shown to be the Zn and Cd transporter. We identified two closely related copies of *NtZIP4A* and *NtZIP4B* having all structural determinants characteristic for *ZIP* proteins. Of importance is the fact, that expression pattern of both *ZIP4* copies is qualitatively the same indicating functional redundancy between two *NtZIP4* copies. Upregulation by low Zn indicates their involvement in a plant’s strategy in coping with the stress related to Zn-starvation. Furthermore, their threefold higher expression in the leaves compared to roots indicate their importance in providing micronutrients to photosynthesizing organs. NtZIP4B promoter activity examined by determination of GUS staining showed that it was expressed in the root epidermis and in the internal tissues, indicating its participation in Zn acquisition from the soil solution and from the apoplast, thus also in the control of a metal radial root transport supplying minerals to shoots.

Moreover, our study outlines complementary functions of examined *ZIP* genes (*NtZIP1*, *NtZIP2*, *NtZIP4, NtZIP5*-like, *NtZIP8*, *NtIRT1-like*, and *NtIRT1)* in a response of tobacco plants to low-to-high Zn status, and points to their distinct roles in different root parts. Interestingly, we showed that tested genes had different expression pattern in the apical and basal root segments under control conditions and on Zn deprivation which clearly points to their distinct functions in these root regions. Root born processes are of key importance in the efficiency of root-to-shoot translocation of Zn and other metals (Ricachenevsky et al., 2015). Our study clearly indicates that tested *ZIPs* play an important role in these root-specific processes. To clarify their distinct and overlapping roles in different root parts more detailed analysis is needed including tissue- and cell-specific expression under a range of Zn statuses in different root parts. Better understanding of underlying processes would help in engineering plants tolerant to low Zn as well as plants more useful for phytoremediation of metal contaminated soil.

## Author Contributions

AB contributed to the study concept and writing manuscript, carried out all experiments, and performed data analysis. MP was involved in yeast study, expression analysis, and GUS assay. AP and KK contributed to cloning and tobacco transformation. MK was involved in expression analysis and hydroponic experiments. LW supervised yeast complementation assays. DMA designed the study concept, coordinated the research and supervised experiments, performed data analysis, and wrote the manuscript.

## Conflict of Interest Statement

The authors declare that the research was conducted in the absence of any commercial or financial relationships that could be construed as a potential conflict of interest.

## Footnotes

1 http://web.expasy.org/translate/

2 https://embnet.vital-it.ch/software/TMPRED_form.html

3 http://eu.idtdna.com/calc/analyzer

4 http://www.basic.northwestern.edu/biotools/oligocalc.html

## Supplementary Material

FILE S1 List of primers for cloning and expression analysis; nucleotide sequences used for cloning of *NtZIP4B*.

FILE S2 Stability of *PP2A*.

FILE S3 Supplementary information for cloning of *NtZIP4B* promoter (primers, promoter sequences).

FILE S4 Genomic sequences of *NtZIP4A* and *NtZIP4B*.

FILE S5 Summary information on mRNA and genomic sequences of *NtZIP4A* and *NtZIP4B*; sequence identity between NtZIP4A, NtZIP4B, and ZIP predicted proteins from selected species.

FILE S6 Bioinformatics analysis of NtZIP4B subcellular localization.
