# Supplementary material for: Functional Analysis of NtZIP4B and Zn Status-Dependent Expression Pattern of Tobacco ZIP Genes
Source: Front Plant Sci. 2019 Jan 10;9:1984. doi: 10.3389/fpls.2018.01984 (PMC6335357; doi:10.3389/fpls.2018.01984)

Supplementary File S1;

Content:

A1. The list of primer sequences used for cloning of ORF of *NtZIP4B* and for expression analysis

A2. The list of primer sequences used for expression analysis of tobacco *ZIP* genes

B. Comparison of four nucleotide sequences of cDNA used for identification of *NtZIP4A* and *NtZIP4B*:

C. Specificity of primers used for expression analysis of *NtZIP4A* and *NtZIP4B*

A1. The list of primer sequences used for cloning of ORF of *NtZIP4B* and for expression analysis

name	sequence
**For ORF cloning**	
ZIP4B-ORF-START	CACCATGTCGTTCACTGAGGATCTCGTGCCC
ZIP4B-END	TGCCCATATTGCAAGAAGGGACATGAGTC
ZIP4B-STOP	TCATGCCCATATTGCAAGAAGGGACATGAGTC
**For expression analysis**	
ZIP4A-F-v1	CTGTTTCCAATACCACCTGT
ZIP4A-R-v1	GCTTCTTGCCAACTAATGGA
ZIP4B-F-v1	TCTGTTTCCAATATTACCTGC
ZIP4B-R-v1	TTCTTGCCAACTAACGGG

CACC – sequence present at 5’ end to clone the OFR in the proper orientation

A2. The list of primer sequences used for expression analysis of tobacco *ZIP* genes

gene	Accession no	primer sequences	lenght of amplicon
NtZIP1	AB505626.1	F: TGGTGGCTCAGTCTGGAGAT	94
		R: CGAAGGAGCTCAGAACTGGAA	
NtZIP2	XM_016617597.1	F: CACCATGTTTAGTGACTGC	136
		R: CTTGAGAAAAGGATTTGCTTCC	
NtZIP5-like	XM_016594002.1	F: TCTGCGAAAAATGGTGTG	118
		R: GAAGGAGCTCGGAATCAG	
NtZIP8	XM_016603305.1<br>XM_016586286.1	F: GGTTGTGCCATTAGAAGAGG	163
		R: AGTTAATGCCGCTACAAGG	
NtIRT1	AB263746.1	F: CGCAATAACAACTCCATTCG	134
		R: AAGCCATATAGATCAGAAGGC	
NtIRT1like	XM_016611068.1	F: CTTCTTCGCAGTAACAACC	139
		R: AGCCATGTAAATAAGAAGACC	
NtPP2A	NM_001325282.1	F: GCACATTCATTCAGTTTGAACC	142
		R: GTAGCATATAAAGCAGTCAGC	

Primers for *NtZIP1*, *NtZIP2*, *NtIRT1*, *NtIRT1like*, *PP2A* – the same as in Barabasz et al. (2016);
Primers for *NtIRT1*-like – based on sequence T-XIII-A9 published by Barabasz et al. (2016) which is 100% homologous with the sequence XM_016611068.1.

B. Comparison of four nucleotide sequences of cDNA used for identification of *NtZIP4A* and *NtZIP4B*:

JZ875395.1 –EST partial sequence of *NtZIP4* (identified by Barabasz et al., 2016, initially named as T-XIII-K12) used as a query sequence to identify in the NCBI data base the full sequence of tobacco *NtZIP4* gene;
XM_016647965.1 (named in the current paper as *NtZIP4A*) – sequence available in the data base with the 99% homology to JZ875395.1;
XM_016586154.1 (named in the current paper as *NtZIP4B*) – sequence available in the data base with the 97 % homology to JZ875395.1;
Cloned *NtZIP4B* – sequence of ORF + STOP codon, cloned in the current paper

With the use of primer pairs ZIP4B-ORF-START / ZIP4B-END (for cloning without the STOP codon), and ZIP4B-ORF-START / ZIP4B-STOP (for cloning with the STOP codon) we cloned the ORF of *NtZIP4B* gene.

In yellow - nucleotides different between given sequences
In blue - nucleotides different between cloned NtZIP4B and the sequence XM_016586154.1.

Comparison between *NtZIP4B* and *ZIP4* sequences of other tobacco varieties (Supplementary File S3) showed that the cloned *NtZIP4B* sequence is identical with the genomic copy of K326 i K326 Nitab4.5_0003621varieties.

Underlined in green – sequence identical with the primer sequence ZIP4B-ORF-START used for cloning of the ORF of *NtZIP4B*.
Underlined in orange – sequence complementary to primer sequence ZIP4B-ORF-STOP used for cloning of *NtZIP4B*.
Underlined in blue double line – sequence identical or complementary to the primer sequences used for *NtZIP4A* expression analysis by RT-qPCR.
Underlined in orange double line – sequence identical or complementary to the primer sequences used for *NtZIP4B* expression analysis by RT-qPCR

```
JZ875395.1               ------------------------------------------------------------     0
XM_016647965.1/ZIP4A     ACAGTGGAGCTCATTCTCTTCTAAAACAAGCGACTGTATCAAAGTCTCCCCATCTCCTCT     60
XM_016586154.1/ZIP4B     -------------------------------GACTGTATCAAAGTCTCCCCATCTCCTCT     29
ClonedZIP4B              ------------------------------------------------------------     0

JZ875395.1               ------------------------------------------------------------     0
XM_016647965.1/ZIP4A     CTCTTCTTTCTTTACTAATAATGTCGACATCATTCCCAGTATCTTCTTCCATTTCTCATC     120
XM_016586154.1/ZIP4B     CTCTTCTTTCTTTACTAATAATGTCGACATCATTCCCTGTATCTTCTTCCTTTTCTCGTC     89
ClonedZIP4B              ------------------------------------------------------------     0

JZ875395.1               ------------------------------------------------------------     0
XM_016647965.1/ZIP4A     CCCATCTATTTTCCCCTTCTTTCATTCTTTCTTTATCCTTTTCTAAACCCTCCCTAGTCC     180
XM_016586154.1/ZIP4B     CCCATCTATTTTCCCTTTCTTTCTTTCTTTCTTTATCCATTTCTAAATCTTCCCTACTCC     149
ClonedZIP4B              ------------------------------------------------------------     0

JZ875395.1               ------------------------------------------------------------     0
XM_016647965.1/ZIP4A     CTCCTTACAACAGCAAGAGGATTTATAAAACAGTGTTTTCGGACAAAAGGGGTTTGTTCT     240
XM_016586154.1/ZIP4B     CTCCT-ACAACAACAACAGGATTTATAAAACAG---------ACAAAAGGTGTTTGTTCT     199
ClonedZIP4B              ------------------------------------------------------------     0
```

```
JZ875395.1               ------------------------------------------------------------    0
XM_016647965.1/ZIP4A     CGTCAAGAATTATTACAAAATTCACCACTTTCTTGTTAGATTAATACTATAATTTTGGTC    300
XM_016586154.1/ZIP4B     CGTCAAGATTTATTACAAAATTCACCACTTTCGT--------------ATAATTTTGGTC    245
ClonedZIP4B              ------------------------------------------------------------    0

JZ875395.1               ------------------------------------------------------------    0
XM_016647965.1/ZIP4A     AAGACCTTTCACCTATATATAGAGATTTGTGATGCTTTCTCTGTTTGTGTAAACTTGTTA    360
XM_016586154.1/ZIP4B     AAGACCCTTCAGCTATATATAGAGATTTGTGTTAGTGTTATGCTTTCT--CTGTTTATTA    303
ClonedZIP4B              ------------------------------------------------------------    0

JZ875395.1               ------------------------------------------------------------    0
XM_016647965.1/ZIP4A     CGTAATTGGAAATAG-CGTTTTGAGGGACAAATTCCCTCTCTTTTCCCCATATCCCATGT    419
XM_016586154.1/ZIP4B     CGTAATTGGAAATAGAGCTTTTGAGGGACAAATTCCCTCTCTTTTCCACATATCCCATGT    363
ClonedZIP4B              --------------------------------------------------------ATGT    4

JZ875395.1               ------------------------------------------------------------    0
XM_016647965.1/ZIP4A     CGTTCATTGAGGATCTCGTGCCCTTCTTTTTTATGGACCCAAAATCTAGAGAAAAGACTG    479
XM_016586154.1/ZIP4B     CGTTCACTGAGGATCTCGTGCCCTTCTTTTTTATGGACCCAAAAATTAGAGAAAAGACCG    423
ClonedZIP4B              CGTTCACTGAGGATCTCGTGCCCTTCTTTTTTATGGACCCAAAAATTAGAGAAAAGACCG    64

JZ875395.1               ------------------------------------------------------------    0
XM_016647965.1/ZIP4A     GGGCTTTCTCAGATACCATTATGCTGAAACTTTATCAATCTGTTTCCAATACCACCTGTG    539
XM_016586154.1/ZIP4B     GGGCTTTCTCAGATACCGTTATGCTGAAACTTTATCAATCTGTTTCCAATATTACCTGCG    483
ClonedZIP4B              GGGCTTTCTCAGATACCGTTATGCTGAAACTTTATCAATCTGTTTCCAATATTACCTGCG    124

JZ875395.1               ------------------------------------------------------------    0
XM_016647965.1/ZIP4A     GCAGTGCTGATGAAGAAATAGAAGGCTGCCGAGACAGCTCGGCTGCTCTCACCCTTAAAA    599
XM_016586154.1/ZIP4B     GCAGTGCTGATGAAGAGATAGAAGGCTGCCGAGACAGCTCGGCTGCTCTTACCCTTAAAA    543
ClonedZIP4B              GCAGTGCTGATGAAGAGATAGAAGGCTGCCGAGACAGCTCGGCTGCTCTTACCCTTAAAA    184

JZ875395.1               ------------------------------------------------------------    0
XM_016647965.1/ZIP4A     TTGTGGCTATCTCTGCCATCCTCATAGCTAGCACTTGCGGAGTTGGTATTCCATTAGTTG    659
XM_016586154.1/ZIP4B     TCGTGGCTATCTCTGCCATCCTAATAGCTAGTACTTGCGGAGTTGGTATCCCGTTAGTTG    603
ClonedZIP4B              TCGTGGCTATCTCTGCCATCCTAATAGCTAGTACTTGCGGAGTTGGTATCCCGTTAGTTG    244

JZ875395.1               ------------------------------------------------------------    0
XM_016647965.1/ZIP4A     GCAAGAAGCATCGGTTCCTCCGAACTGACTCCAATCTCTTTCTTACTGTTAAAGCCTTTG    719
XM_016586154.1/ZIP4B     GCAAGAAGCATCGGTTCCTCCGAACTGACTCTAATCTCTTTCTTGCTGTTAAAGCCTTTG    663
ClonedZIP4B              GCAAGAAGCATCGGTTCCTCCGAACTGACTCTAATCTCTTTCTTGCTGTTAAAGCCTTTG    304

JZ875395.1               ------------------------------------------------------------    0
XM_016647965.1/ZIP4A     CTGCTGGTGTCATCCTCTCTACAGGATTTGTCCACATATTACCAGGCGCCACCTCATCAT    779
XM_016586154.1/ZIP4B     CTGCTGGTGTCATCCTCTCTACAGGCTTTGTCCACATATTACCAGGCGCCACCTCATCAT    723
ClonedZIP4B              CTGCTGGTGTCATCCTCTCTACAGGCTTTGTCCACATATTACCAGGCGCCACCTCATCAT    364

JZ875395.1               ------------------------------------------------------------    0
XM_016647965.1/ZIP4A     TAACTAATCCTTGCCTTCCGAAATTTCCTTGGTTGAAATTCCCTTTTGCTGGTTTTATTG    839
XM_016586154.1/ZIP4B     TAACTAATCCTTGTCTTCCGAAATCTCCTTGGTTGAAATTCCCTTTCGCTGGTTTTATCG    783
ClonedZIP4B              TAACTAATCCTTGTCTTCCGAAATCTCCTTGGTTGAAATTCCCTTTCGCTGGTTTTATCG    424

JZ875395.1               ------------------------------------------------------------    0
XM_016647965.1/ZIP4A     CTATGATGGCTGCATTGGCTACGTTGGTGGTTGACTTTGTTGGGACTCAGTATTATGAGA    899
XM_016586154.1/ZIP4B     CCATGATGGCTGCATTGACTACCTTGGTGGTTGACTTTGTTGGGACTCAGTATTATGAGA    843
ClonedZIP4B              CCATGATGGCTGCATTGACTACCTTGGTGGTTGACTTTGTTGGGACTCAGTATTATGAGA    484

JZ875395.1               ------------------------------------------------------------    0
XM_016647965.1/ZIP4A     GGAAGCAAGAGAAACAAAGCCAAAAAGATCAGATTGATTCAGTGGATTTGGTGTCAGAAT    959
XM_016586154.1/ZIP4B     GGAAGCAAGAGAAACAAAGCCAAAAAGATCAGATTGATTCAGTGGATTTGGTGTCAGAAT    903
ClonedZIP4B              GGAAGCAAGAGAAACAAAGCCAAAAAGATCAGATTGATTCAGTGGATTTGGTGTCAGAAT    544

JZ875395.1               --------GTACCGGTTGAACCAAAGGCAAGGAATGAGAAATTGTTTGGTGAAGAAGACG    52
XM_016647965.1/ZIP4A     CAGCTATTGTACCGGTTGAACCAAAGGCAAGGAATGAGAAATTGTTTGGTGAAGAAGACG    1019
XM_016586154.1/ZIP4B     CAGCTATTGTACCAGTTGAACCAAAGGCAGGGAATGAGAAATTGTTTGGTGAAGAAGATG    963
ClonedZIP4B              CAGCTATTGTACCAGTTGAACCAAAGGCAGGGAATGAGAAATTGTTTGGTGAAGAAGATG    604
```

```
                                        ***** *************** **************************** *

JZ875395.1                 GTGGTGCAATACACATTGTTGGGATGCATGCACATGCAGCTCATCACAGACATAGCCATT    112
XM_016647965.1/ZIP4A       GTGGTGCAATACACATTGTTGGGATGCATGCACATGCAGCTCATCACAGACATAGCCATT    1079
XM_016586154.1/ZIP4B       GTGGTGCAATACACATTGTTGGGATGCATGCACATGCAGCTCATCACAGACATAGCCATT    1023
ClonedZIP4B                GTGGTGCAATACACATTGTTGGGATGCATGCACATGCAGCTCATCACAGACATAGCCATT    664
                           ************************************************************

JZ875395.1                 CACAAGAACAAGGGGCATGTCAAGGGAACGTGGGGGAGCATTCCCATGGTCATTCGCACT    172
XM_016647965.1/ZIP4A       CACAAGAACAAGGGGCATGTCAAGGGAACGTGAGGGAGCATTCCCATGGTCATTCGCACT    1139
XM_016586154.1/ZIP4B       CACAAGAACAAGGGGCATGTCAAGGGAACGTGAGGGAGCATTCCCATGGTCATTCGCACT    1083
ClonedZIP4B                CACAAGAACAAGGGGCATGTCAAGGGAACGTGAGGGAGCATTCCCATGGTCATTCGCACT    724
                           ******************************** ***************************

JZ875395.1                 CCCATAGCTTTGGTGGTGGAGATGAGGAAGGTGGAGGGAGGCATGTTGTTGTTTCTCAGG    232
XM_016647965.1/ZIP4A       CCCATAGCTTTGGTGGTGGAGATGAGGAAGGTGGAGGGAGGCATGTTGTTGTTTCTCAGG    1199
XM_016586154.1/ZIP4B       CCCATAGCTTTGGTGGTGGAGATGAGGAAGGTGGAGGGAGGCATGTTGTTGTTTCTCAGG    1143
ClonedZIP4B                CCCATAGCTTTGGTGGTGGAGATGAGGAAGGTGGAGGGAGGCATGTTGTTGTTTCTCAGG    784
                           ************************************************************

JZ875395.1                 TCTTGGAGCTGGGGATAGTATCACATTCTCTCATAATAGGCATAGCATTGGGTGTTTCAG    292
XM_016647965.1/ZIP4A       TCTTGGAGCTGGGGATAGTATCACATTCTCTCATAATAGGCATAGCATTGGGTGTTTCAG    1259
XM_016586154.1/ZIP4B       TCTTGGAGCTGGGAATAGTATCACATTCTCTCATAATAGGCATAGCATTGGGTGTTTCAG    1203
ClonedZIP4B                TCTTGGAGCTGGGAATAGTATCACATTCTCTCATAATAGGCATAGCATTGGGTGTTTCAG    844
                           ************* **********************************************

JZ875395.1                 AAAGTCCGTGCACAATTAGACCCTTGCTTGTGGCCTTGTCGTTCCACCAGTTCTTCGAAG    352
XM_016647965.1/ZIP4A       AAAGTCCGTGCACAATTAGACCCTTGCTTGTGGCCTTGTCGTTCCACCAGTTCTTCGAAG    1319
XM_016586154.1/ZIP4B       AAAGTCCATGCACAATTAGACCCTTGCTCGTGGCCTTATCGTTCCACCAGTTCTTCGAAG    1263
ClonedZIP4B                AAAGTCCATGCACAATTAGACCCTTGCTCGTGGCCTTATCGTTCCACCAGTTCTTCGAAG    904
                           ******* ******************** ******** **********************

JZ875395.1                 GTTTTGCGTTAGGAGGCTGCATCTCACAGGCACAGTTCAATTCCCTCCGTTCCACTATAA    412
XM_016647965.1/ZIP4A       GTTTTGCGTTAGGAGGCTGCATCTCACAGGCACAGTTCAATTCCCTCCGTTCCACTATAA    1379
XM_016586154.1/ZIP4B       GTTTTGCGTTAGGAGGTTGCATCTCGCAGGCACAGTTCAATTCCCTCCGTTCCACTATAA    1323
ClonedZIP4B                GTTTTGCGTTAGGAGGTTGCATCTCGCAGGCACAGTTCAATTCCCTCCGTTCCACTATAA    964
                           **************** ******** **********************************

JZ875395.1                 TGGCAACGTTTTTCGCCGTAACAACACCCTTGGGAATTGCTATAGGGATTCTAGCTTCTT    472
XM_016647965.1/ZIP4A       TGGCAACGTTTTTCGCCGTAACAACACCCTTGGGAATTGCTATAGGGATTCTAGCTTCTT    1439
XM_016586154.1/ZIP4B       TGGCAACGTTTTTCGCCGTAACAACACCCTTGGGAATTGCTATAGGAATTCTAGCTTCTT    1383
ClonedZIP4B                TGGCAACGTTTTTCGCCGTAACAACACCCTTGGGAATTGCTATAGGAATTCTAGCTTCTT    1024
                           ********************************************** *************

JZ875395.1                 CGTCGTAC----------------------------------------------------    480
XM_016647965.1/ZIP4A       CGTCGTACAATCCACATAGCCCAAGAGCTTTGGTGGTGGAAGGGATCCTAAACTCTATAT    1499
XM_016586154.1/ZIP4B       CATCTTACAATCCACATAGCCCAAGAGCTTTGGTAGTGGAAGGGAGCCTTAACTCTATAT    1443
ClonedZIP4B                CATCTTACAATCCACATAGCCCAAGAGCTTTGGTAGTGGAAGGGAGCCTTAACTCTATAT    1084
                           * ** ***

JZ875395.1                 ------------------------------------------------------------    480
XM_016647965.1/ZIP4A       CTTCTGGAATTCTAATCTACATGGCTTTAGTAGACCTAATTGCTGCAGATTTCTTGAGTA    1559
XM_016586154.1/ZIP4B       CTGCTGGAATTTTAATCTACATGGCTTTAGTAGACCTAATTGCTGCAGATTTCTTGAGTA    1503
ClonedZIP4B                CTGCTGGAATTTTAATCTACATGGCTTTAGTAGACCTAATTGCTGCAGATTTCTTGAGTA    1144

JZ875395.1                 ------------------------------------------------------------    480
XM_016647965.1/ZIP4A       AGAGAATGAGCTGCAATACAAGGCTTCAAATAGTATCTTATTTTGCACTATTCTTAGGGG    1619
XM_016586154.1/ZIP4B       AAAGAATGAGCTGCAATACAAGGCTTCAAATAGTATCTTATTTTGCACTATTCTTAGGGG    1563
ClonedZIP4B                AAAGAATGAGCTGCAATACAAGGCTTCAAATAGTATCTTATTTTGCACTATTCTTAGGGG    1204

JZ875395.1                 ------------------------------------------------------------    480
XM_016647965.1/ZIP4A       CTGGACTCATGTCCCTTCTTGCAATATGGGCATGATATTGCTTTTTATTATA-TGCATGT    1678
XM_016586154.1/ZIP4B       CTAGACTCATGTCCCTTCTTGCAATATGGGCATGATATTGCTTTTTTATTATATGCATGT    1623
ClonedZIP4B                CTGGACTCATGTCCCTTCTTGCAATATGGGCATGA-------------------------    1239

JZ875395.1                 ------------------------------------------------------------    480
XM_016647965.1/ZIP4A       AATTTTTTTGTTTTTATATTTTATTAACCACAAAAAAGCCAAGCAAGCAAGCAATTGTTT    1738
XM_016586154.1/ZIP4B       AATAATTTTTTTTTTT------TTTGGCTAACCAAAAAAAAAGTCAAGCAAGCAATTGTTT   1677
ClonedZIP4B                ------------------------------------------------------------    1239

JZ875395.1                 ------------------------------------------------------------    480
XM_016647965.1/ZIP4A       ACCATTTCTTTTTCCTGATTTTTACTTTTTCTTATCCTTGAGAGGATGTTGTTTTGGGCC    1798
XM_016586154.1/ZIP4B       ACCATATCTTTTTTTCCTGATT----------------------TTACTTTGTGAGCC    1713
```

```
ClonedZIP4B              ------------------------------------------------------------         1239


JZ875395.1               ------------------------------------------------------------         480
XM_016647965.1/ZIP4A     CTGGCTGTGGAAGGGTGCTTGTGTAATAGGTATGGCA-CCCTGGTAGTAGGGTTGAAGAT         1857
XM_016586154.1/ZIP4B     CTCGTTGTGGAAGGGTGCTTTTGTAATAGGTATGGCAACCCTGTTAGTAGGGTTGAAGTT         1773
ClonedZIP4B              ------------------------------------------------------------         1239


JZ875395.1               ------------------------------------------------------------         480
XM_016647965.1/ZIP4A     GAAGTTGCAAACAGAAGAGAGAGAAGAAGAGAAATATTGCATTTAATTCATGCAATCAAA         1917
XM_016586154.1/ZIP4B     GAAGTTGCAAACTGAAAAGAGAAAAGA---GAAATATTGCATTTAA--------------         1816
ClonedZIP4B              ------------------------------------------------------------         1239


JZ875395.1               ------------------------------------------------------------         480
XM_016647965.1/ZIP4A     TAATTTAAAGTTCTTTTCTATGTTGTATGATCATTTTCCTTATTTTGAGATTTGATTATG         1977
XM_016586154.1/ZIP4B     ------------------------------------------------------------         1816
ClonedZIP4B              ------------------------------------------------------------         1239


JZ875395.1               --------------------  480
XM_016647965.1/ZIP4A     ATTATGATTTTCAGAGGCAA  1997
XM_016586154.1/ZIP4B     --------------------  1816
ClonedZIP4B              --------------------  1239
```

C. Specificity of primers used for expression analysis of *NtZIP4A* and *NtZIP4B*.

(C1) As a template plasmid pRACE with the partial sequence of *NtZIP4A*, from 144 bp – to 1231 bp of the sequence XM_016647965.1/ZIP4A was used (as marked with a black arrow over the sequence at part B above).

(C2) As a template plasmid pENTR-NtZIP4B-STOP with the full sequence of the ORF of *NtZIP4B* was used.

C1

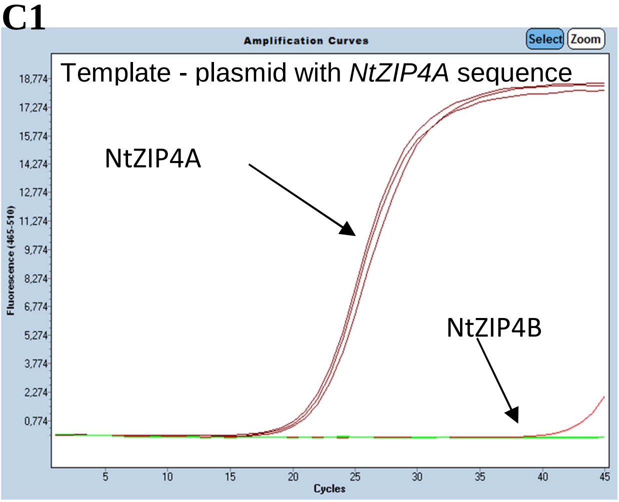

C2

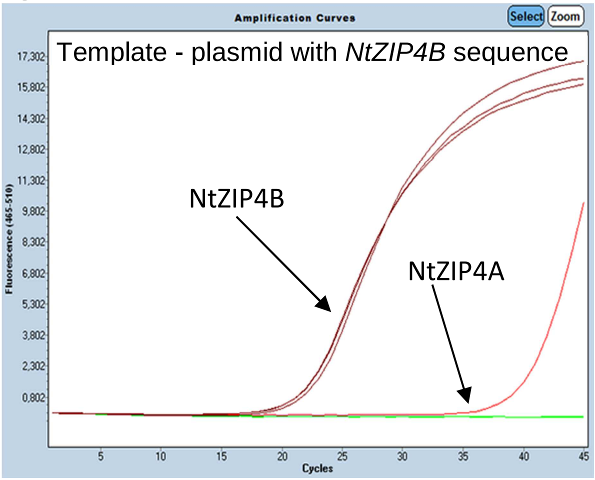

Supplement: FILE S1 — List of primers for cloning and expression analysis; nucleotide sequences used for cloning of NtZIP4B. [file Data_Sheet_1.PDF]
