# Supplementary material for: Functional Analysis of NtZIP4B and Zn Status-Dependent Expression Pattern of Tobacco ZIP Genes
Source: Front Plant Sci. 2019 Jan 10;9:1984. doi: 10.3389/fpls.2018.01984 (PMC6335357; doi:10.3389/fpls.2018.01984)
Supplement: FILE S2 — Stability of PP2A. [file Data_Sheet_2.PDF]

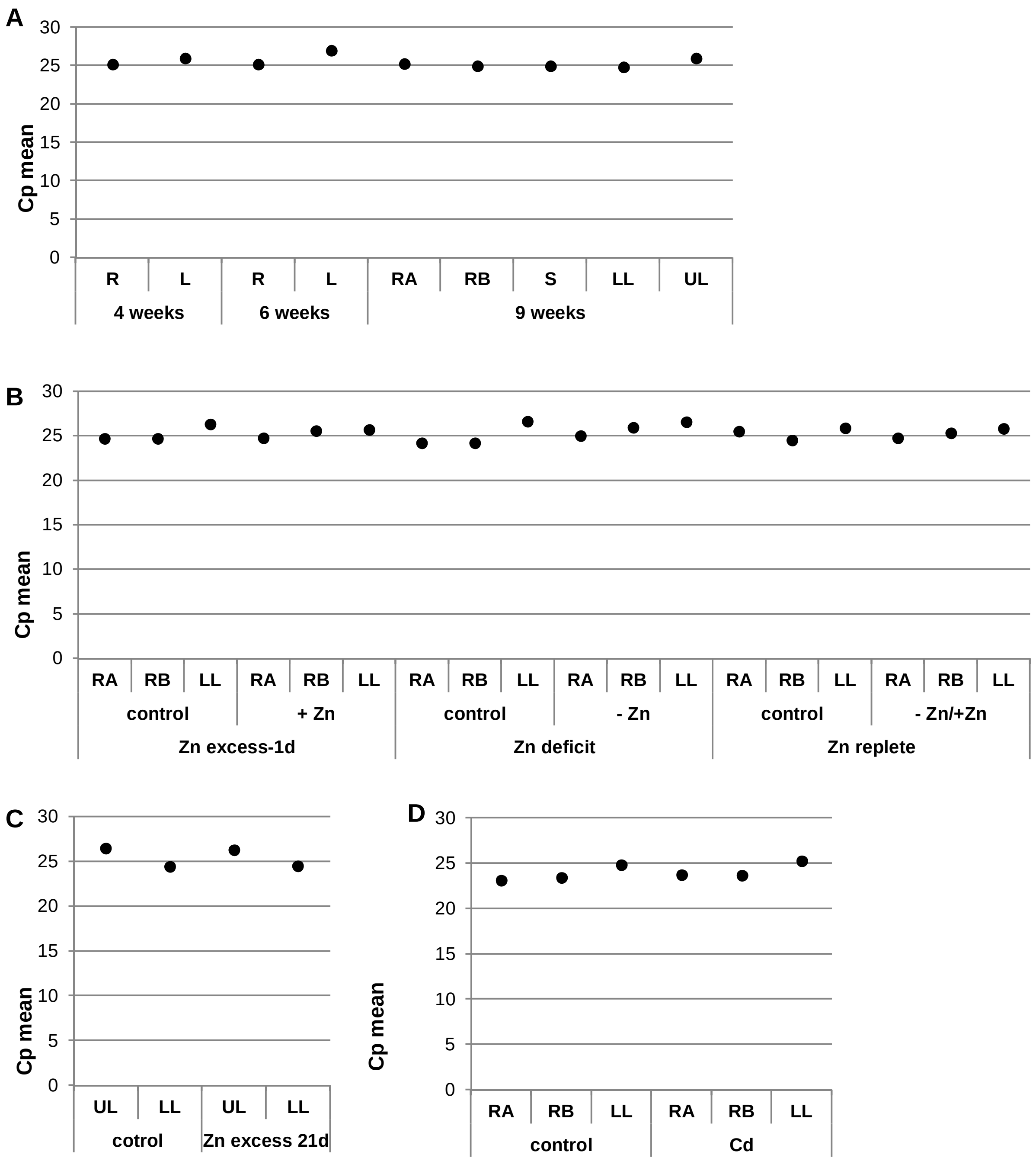

Supplementary File S2 : RNA transcription levels of *PP2A* gene, presented as Cp mean values in diffrent samples: (A) in whole roots, whole leaves, stems, apical and basal segments of roots, young leaves and old leaves from plants grown at control conditions for 4 weeks, 6 weeks and 9 weeks; (B) under various Zn conditions; plants were grown in standard nutrient solution (control) and then transferred into modified control media: supplemented with 50 μM Zn for 1 day (1d); without Zn for 4 days (4d - Zn deficiency); plants grown at Zn-deficiency conditions for four days were transferred to the control medium for two days (6d - Zn replete).

(C) in upper and lower leaves of plants grown at control medium and in the presence of 50 μM Zn for twenty-one days; (D) in the apical and basal parts of the roots and in lower leaves of plants grown at control medium and in the presence of 4 μM Zn for three days
