# Supplementary material for: Functional Analysis of NtZIP4B and Zn Status-Dependent Expression Pattern of Tobacco ZIP Genes
Source: Front Plant Sci. 2019 Jan 10;9:1984. doi: 10.3389/fpls.2018.01984 (PMC6335357; doi:10.3389/fpls.2018.01984)
Supplement: FILE S3 — Supplementary information for cloning of NtZIP4B promoter (primers, promoter sequences). [file Data_Sheet_3.PDF]

Supplementary File S3

Content:

A. Primers used for cloning of *NtZIP4B* promoter
B. Comparison of the promoter sequences including 5'UTR of *NtZIP4A* and *NtZIP4*
C. Comparison of the promoter sequences including 5'UTR of *NtZIP4A* from three tobacco cultivars: NT90, K326 and Basma Xanthi
D. Comparison of the promoter sequences including 5'UTR of *NtZIP4B* from three tobacco cultivars: NT90, K326 and **Basma Xanthi**

A. Primers used for cloning of *NtZIP4B* promoter

Primer Forward	promZIP4B-F	CACCCCAAACTATACCAACTTACATTATTGG
Primer Reverse	promZIP4B-R	CATGGGATATGTGGAAAAGAGAG

CACC – sequence added to clone the insert into the pENTR plasmid in propper orientation.

Cloned promoter sequence of *NtZIP4B* + 5'UTR + START codon:

In red - START codon.

In red underlined - sequence of the forward primer and the sequence complementary to the reverse primer.

```
CCAAACTATACCAACTTACATTATTGGGTGTTTGGATTGGTTTTTAGGCTAGTCAAATCAACTTTTAAATTCTTTTTAACCTTTTTTAGTATTTGGCAAAG
CTATAAAGTGCTTAAAATAAATTAAAAACTGCTTAAAACAAGCCAAAGAAACAAGCTGACCAATCTCAACTTATTGCTTTTTGGCTTAAAAACTATTTCTG
CTGAAAAGTCATTTTTATAATCTAATCCAAACAGACCCTAAGTGAATTAATGAAGTAAAACAATGTTAATTAACTATGGTTTATTGAGAAAAATTATGCAA
AGCATCCAATGGTTTTGCCAGCTTGATTTACTTTAATATTCAAATTACCTAAGATTACTTGATAATGCATGAGTTTTTCTACACCAAGTGAAATTGACATA
TAATATAATAACTTAGAAAATAGAGTAATAACGTATATGTCTGTTCTTATATTTAAACTCATATATAACCACTAACAATTGCCCTAAAGGATAATGGGGGA
AAAAGAAAGAAAAAGCACATACAAAGGTGTAACCTCAAATAACCTATACATTTTACCTCAAATCACATTTAATTTATGTGCTTTTACATCACTTAATAAGG
ATAAATCAATGTGATTCGATGTACAAATGAGGATGTGGATAAATATAATTTTCAAAGGTTTGAATTCGAACGTTGAGCCTTATACTATGGGAGGGCAATCC
GGTCCACGAAATAATATTCTGCATCCATGCATGGTCCAGGGAAGGGTTGTACCTAAAGGTAGGGGTGTTCATAAAAACCCAAAAAATCGAACCAAACCGAA
AATCAAACCAAGCCGATCAAAAAAACCAATACTTTTTGGTTTGGTTTGGTTTTGGTTTTGAATTTTAAAAACCGATCAAACTTGGTTTGGTTTTGGTTTTA
ATCAAAAAATAACCGGAAAAAACCGAACCAAACCAACTAAAGGAGTAGCTATTTCAAATTTATTATTACACCTATATATATGTATATTTTTATACAAAGTT
TCAAAAATTTTATGACGAAAGTTAATCGTTTGCACTTTTAGTATAGTTCTTTACCTTTACATTCTAGTTTGATTGGTAGTTTTCTTTTGTTAAGTGTAAGA
ATCCATTTCATGTTTAAAAAAAAATATATTTTTAATTGAGTCCTTAAATTATTCATCACCATTTGATTCAATTATCATCAATATATCTTAGTAAATAATAG
ATTTCTCAAAGGACAATTGATTTGATAGTGTTACGTTGAAAATATGGTCGCCAGAATATGTGTTTGGTAGTGTATGTCTTATATTTAAGAAAAAACCGAAA
AATAACCGAAAAAACCGACTAAACCCGACATTAACTGAATCGAGAAAAAACCAACTTAATTGGTTTGGTTTGATTCGAATATTTGAAAAACCGACTTACTT
GGTTTGATTTCTTTTTAGGGAAAAACCGAACCAAACCGAACCATGAACACCCCTACCTAAAGGGAAATGATGTATGCAATCTATCCTGAGTATCGGTGGCT
GATTCCACGACTCGAATTCATGACCTATAGATCATGCAGAGACAACTTTATTACTGCTCCGAGACTCTCCTTAAGCTTTATACAATGTGAATTCGAATTAA
TTGCCATTTCAAATTAGATATAAAATACCTTAAAAAATAAATATTTTTACTCTGAAAATGCACATTGGCTCAGACAACTACTAATATGTTATTAAAAAATC
GAAAAAAGGAAATAAAAAGTGGAGTGCATGACTTATTACGCAGAGGTGTCGACACAGTGGAGCTCATTCTCTTGTAAAACAAGCGACTGTATCAAAGTCTC
CCCATCTCCTCTCTCTTCTTTCTTTACTAATAATGTCGACATCATTCCCTGTATCTTCTTCCTTTTCTCGTCCCCATCTATTTTCCCTTTCTTTCTTTCTT
TCTTTATCCATTTCTAAATCTTCCCTACTCCCTCCTACAACAACAACAGGATTTATAAAACAGACAAAAGGTGTTTGTTCTCGTCAAGATTTATTACAAAA
TTCACCACTTTCGTATAATTTTGGTCAAGACCCTTCAGCTATATATAGAGATTTGTGTTAGTGTTATGCTTTCTCTGTTTATTACGTAATTGGAAATAGAG
CTTTTGAGGGACAAATTCCCTCTCTTTTCCACATATCCCATG
```

B. Comparison of the promoter sequences including 5'UTR of *NtZIP4A* (from the contig AWOK01066417.1 position 1230..4748 – without ATG), **and *NtZIP4B*** (from the contig AWOK01166346.1 position 9067..12566 – without ATG).

For comparison made by Clustal Omega https://www.ebi.ac.uk/Tools/msa/clustalo/ the sequences of 3519 bp (for *NtZIP4A*) and 3500 bp (for *NtZIP4B*) above the START codon were used.
The program PlantCARE: http://bioinformatics.psb.ugent.be/webtools/plantcare/html/ , was used to identify *cis*-acting elements within the promoter region. For analysis the 1500 bp fragment of the promoter region (counting from the START codon upstream) was used.

In green - ZDRE sequence (Zinc Deficiency Response Element – Assunção et al., 2010), localized at -335 bp (for *NtZIP4A*prom) and -310 bp (for *NtZIP4B*prom).
In red - START codon.
small letters – putative 5'UTR sequences present in the mRNA reference sequences (XM_016647965.1 and XM_016586154.1).
In pink – first bases of the EST sequence available in the data base with 100% homology to putative 5'UTR from the mRNA reference sequence
In red underlined – sequence of the forward primer and the sequence complementary to the reverse primer which were used to clone the promoter sequence of 2160 bp including 5'UTR.
* depicts identical nucleotides at a given position.

GT1 – light responsive element motif from *Arabidopsis thaliana*;
MBS – MYB binding site involved in drought-inducibility from *Arabidopsis thaliana*;
Skn-1 - cis-acting regulatory element required for endosperm expression motif from *O. sativa*;
Box-1 – light responsive element from *Pisum sativum*. The Box-1 sequence localized within the ERE sequence; ;
CAAT-box – common cis-acting element in promoter and enhancer regions;
TATA-box - core promoter element around -30 of transcription start site;
ERE – ethylene-responsive element from *Dianthus caryophyllus*
G-box – cis-acting regulatory element involved in light responsiveness from *Zea mays*;
LTR – cis-acting element involved in low-temperature responsiveness from *Hordeum vulgare*;
RY-element – cis-acting regulatory element involved in seed-specific regulation from *Helianthus annuus*;
TGACG-motif - cis-acting regulatory element involved in the MeJA-responsiveness from *Hordeum vulgare*;
Circadian - cis-acting regulatory element involved in circadian control from *Lycopersicon esculentum*;

```
 -3519 bp ----------CACTAATAAGAAACAGAAAAAGAAAAACAAGTTGCTGATTTTTTGTTTAA       ZIP4A_AWOK01066417.1
 -3500 bp ATCATATTTCCACATAAAAGTGTTCCGAATATAC--GCCCA-GACCGAGTACGTAAATCG       ZIP4B_AWOK01166346.1
          ***  * ***       ** * *    *      * ** *   *   *

-3469 bp  AGTTTA-----AGAGAAGCATA-AAGAAATGCGTGCAAGTTATATACAGTTAGACATGGT       ZIP4A_AWOK01066417.1
-3443 bp  AGGTGAGGAAAAAGGAGGCTTCTAAGGCCTCGAAACATGAAATTTACTCGTAAATCAAGT       ZIP4B_AWOK01166346.1
          ** * *     *  ** ** *  ***   *     ** *  ** ***   ** *    **

-3415 bp  CAGCATAATTAACCTAATCAATCCTCGTTTTGGA----AGAAATCAAGAATAAAATATGT       ZIP4A_AWOK01066417.1
-3383 bp  GATG-------ACCTTTTGGGTCATCACATTATAACTATGTGACCAATATTTAGTAAGGG       ZIP4B_AWOK01166346.1
          *         ****  *   ** **   **  *     *  * *** * * *   * *

-3361 bp  TCGTTTAATATTAACCTCTTCTTTTATCGACGAATATTC----TCT--GATTCTTTTCTG       ZIP4A_AWOK01066417.1
-3330 bp  TAGTTTAGTCATACTAGGTATTTTTGTATAGGATTTAGCATTTTCTTAATGGGCATGCTA       ZIP4B_AWOK01166346.1
          * ***** *  **     *  **** *  * ** *   *    ***         * **
```

```
-3307 bp  AACCTTTCGTTCTCTTTTTTACT---GTAAAAATAGTCTTATTGACGTATTTTAGTAGAA       ZIP4A_AWOK01066417.1
-3270 bp  AAAGAAAACTGGTCACTTATTATGAACCGAAGGGAGTAAGTTGAAAGAAATCGCCTACTG       ZIP4B_AWOK01166346.1
          **       *  **  ** *  *      **   ***    *  * * * *    **

-3250 bp  ATAGTTTTGGCTGATCATTAAATTATGAAATACGTAAACTTTAATTACATATAATCGATG       ZIP4A_AWOK01066417.1
-3210 bp  CTATTTTTGCCTCTCC-TA-AAATATGAAACAGAGATCTCA---TGATTATTAATCCACT       ZIP4B_AWOK01166346.1
          ** ***** **   * *  ** ******* *   *        * *    ***** *

-3190 bp  TGTTTGGTGAG-AAATCCAACTTT----GATTTTGCGCCAATTAG------AATAACTGT       ZIP4A_AWOK01066417.1
-3155 bp  TAATTAAGCACTAGGTCCACTCTTAGGTGTATGTTTGACAATCAATTTTACTATAACCGA       ZIP4B_AWOK01166346.1
          *  **    *  *  ****   **    *  * *  * **** *        ***** *

-3141 bp  ATTGGAACAAAGAAAATGTGCTTACAAATTAAAACCATAGATTAAGCATCCTACCATGGT       ZIP4A_AWOK01066417.1
-3095 bp  ATTATCACTAGAGTTAGGGGAATACCAATCAAACTGAAAAATCTCACCAAATCGGATAGT       ZIP4B_AWOK01166346.1
          ***   ** *     * * *  *** *** ***   * * **    *    *   ** **

-3081 bp  ACTACTATTCGTA--ACGAAATTTTGATAATCATTTCTTTTAATAATCGTGATGTCCAAA       ZIP4A_AWOK01066417.1
-3035 bp  CAAATCAAACCGATTAACAACTTGTTTGGATG---GTTGTTACTCATTGTATTGTATCGT       ZIP4B_AWOK01166346.1
             *  *  *  *  *  ** ** *    **      * *** * ** **  ***

-3032 bp  TCAGCTTACAGACACGTAGTTTAATTTTACACGATACATGTTATCTCACACTAACTGATG       ZIP4A_AWOK01066417.1
-2978 bp  ---ATTGTTCGATGTTTGTTTTGATTGTTACTTAAATTTATTGTATC-------------       ZIP4B_AWOK01166346.1
               *    **    *  *** *** *     * *  * ** * **

-2963 bp  TATCAGATAACTTTATCCATTAAAAGCTCGAACAAATTGAAAATACTCACCTACTACTAT       ZIP4A_AWOK01066417.1
-2934 bp  GTACCGTTAAATCCGTCGTTACATAA---------CGACGAAATGTGCCACTTTATGTAA       ZIP4B_AWOK01166346.1
             * * *** *   **  *  * *                ****   *  **     **

-2903 bp  TTAAACATTTGGGTTAATATTTTGGGATAAAATTTACCCGTGCGGCCGTACCTGCGACCA       ZIP4A_AWOK01066417.1
-2883 bp  CGACCTATTTGGTGTGGT--CGCGTCGTTACCTTATCTT--------TTTCTCTCAATCT       ZIP4B_AWOK01166346.1
            *   ******  *  *     *   * *  **  *           * *   * * *

-2843 bp  AACTATACCAACTTACATGATTAAGTGAATTAATGAAGTAAAACAATGTTAATTAACTAT       ZIP4A_AWOK01066417.1
-2833 bp  CACCCTTCATTATTATTAAATAATTTTATTTTATCATTTGCCCTATTTTTTTAT-------       ZIP4B_AWOK01166346.1
          **  * *    ***    ** *  * * ** ** *  *     * * ** **

-2783 bp  GGTTAATTGAGAAAAGTATGCAAAGCATCCAATGATTTTGCCAGCTTGATTTACTTTAAT       ZIP4A_AWOK01066417.1
-2780 bp  --------ATAATAATTTTACCCTGTATCATAATTTTTTTTATAATATTGCAAGTTTATT       ZIP4B_AWOK01166346.1
           * ** * * *   * ***  *   ****      *      * **** *

-2723 bp  ATTCAAATTACCTAAAATTACTTGAAAATGCATGAGTTTTTCTACACCG-----------       ZIP4A_AWOK01066417.1
-2728 bp  CTTCATATTGCTGGTGTGTGATCATGAAACGATGACAAACGATACAGTGCAATGCAATAC       ZIP4B_AWOK01166346.1
          **** *** *        *  *    **   ****       ****  *

-2674 bp  ----AGTGAAATTGACATATAATATAAT-------AACTTAAAAAATAGAGTAATAAAGT       ZIP4A_AWOK01066417.1
-2668 bp  AGTACGATACGATACACTATGAAACGATAGGTAACAATCCAAACAAGCTGTAAAAAAATT       ZIP4B_AWOK01166346.1
               *  *   *    *** * *  **       **   *** **      ** *** *

-2625 bp  ATATATCTGTTCTTATATTTAAACTCAATTGCCCTAAAAGATAAAGGGAAAAAAAGAGAA       ZIP4A_AWOK01066417.1
-2608 bp  CGATAAG----GTTTGGTTTGATTTGATTTGGTATTG-------AGTTAAAAAA-TCCGA       ZIP4B_AWOK01166346.1
            ***       **   *** *  * * ***   *         **  ******     *

-2565 bp  AGAAAAAACACATGCAAAGGTGTAACCTCAAATAACCTATACATTTTACCTCAAATCACA       ZIP4A_AWOK01066417.1
-2560 bp  ACCAAACCGACATATAAATATATAATTTTTT-------AT---ATATACTTTTAAGACTT       ZIP4B_AWOK01166346.1
          *  ***   ****  ***  * ***  *          **    * *** *  **

-2505 bp  ATTAATTATGTGCTTTTACATCACTTAATTAGGATAAATCAATGTAATTCGAGGTATAAA       ZIP4A_AWOK01066417.1
-2510 bp  TTATAGAATTTTCTTTAAAAAAA----ATGTCTAGAAATATTTGTGATTCTCCT------       ZIP4B_AWOK01166346.1
          *  *  ** * **** * *  *    **    * ****   *** ****

-2445 bp  TGGGGATTTGAATAAAATATTTTTTCACCTCTTAATTAAAGATTCTGAATTTGAAC-GCT       ZIP4A_AWOK01066417.1
-2460 bp  ------ATGGGATGTAATATTTAGTTAAATATGAA---GTGTTTCCATATGTATTAACTT       ZIP4B_AWOK01166346.1
                * * **  *******  * *  * * **     * ***   ** *       *

-2386 bp  GAAAATAAAATAAAAAAGCTCTAATAAAGTGTATTTTTTCTTTTAATGATCCTTATACGA       ZIP4A_AWOK01066417.1
-2409 bp  TAAAAAGAAATCGTGCAGCTTATGTTAGTTGATTCATTGCTATTGATAAGTCGTAACAAT       ZIP4B_AWOK01166346.1
          ****  ****     ****    * *  **  *  ** ** ** ** *  * **

-2326 bp  T-----GAAAGG-GCAGCTCGGTGCACGAAACAACATCTCGCATTCACGCAGGATCCAGG       ZIP4A_AWOK01066417.1
-2349 bp  GAAGACCAGATGTTAACCTGTGTTATCGT-ACTAAAAATATCTTAAGAAAACGAAGGAGG       ZIP4B_AWOK01166346.1
                * * *   * **  **   **  ** * *  *  * *      * **   ***

-2272 bp  GAAGGGACGCACCCCAATAAGTGTGC---------------CG-TAGGCAGTCTATCTTT       ZIP4A_AWOK01066417.1
-2290 bp  AGAAGGATGTGGAGAAAGGGGGCTGAAGTTGTTTAAAAAGTGGTTACAAGTTAAAACTTT       ZIP4B_AWOK01166346.1
            * *** *      **   *  **                * **      *  * ****

-2228 bp  ATGAAAGTATCAATGACTGATTACACGGCTCAAACCCATAACCTATAAGTCACACAAAGA       ZIP4A_AWOK01066417.1
```

```
-2230 bp  TTTAAAAAATGGGTATATGTTAAATGGGGGCGACCAAATA-------GG--GCGCCCCGT      ZIP4B_AWOK01166346.1
          * ***  **   *   ** * *   **  * * *  ***        *   * *   *

-2168 bp  GACAACTTTATCGTTGCTTCAAGGCTCTCTTTCACCATAGGAATTTGAATAAATTGGCAT      ZIP4A_AWOK01066417.1
-2179 bp  GCAATTTTTACACTTGCGACCAAAC----------TATACCAACTTACATTATTGGGTGT      ZIP4B_AWOK01166346.1
          *  *  ****   ****  * *  *           ***  ** **  ** * * **  *

-2108 bp  T--------------------------------------------TCAAATTAGATAGTA      ZIP4A_AWOK01066417.1
-2129 bp  TTGGATTGGTTTTTAGGCTAGTCAAATCAACTTTTAAATTCTTTTTAACCTTTTTTAGTA      ZIP4B_AWOK01166346.1
          *                                            * *  **   *****

-2092 bp  TCAAATACGGAATGAGAAATTAAAAAATAAATATTTTTACTCTTTGAATGTACATTGG--      ZIP4A_AWOK01066417.1
-2069 bp  TTTGGCAAAGCTATAAAGTGCTTAAAATAAATTAAAAACTGCTTAAAACAAGCCAAAGAA      ZIP4B_AWOK01166346.1
          *     *  *    * *      *********         ***  **    *    *

-2034 bp  -ATCACACAACAATGTTA--TCCTTATATTTTACATAAAACGCCTATTCTTTTT----GT      ZIP4A_AWOK01066417.1
-2009 bp  ACAAGCTGACCAATCTCAACTTATTGCTTTTTGGCTTAAAAACTATTTCTGCTGAAAAGT      ZIP4B_AWOK01166346.1
               *  * **** * *  *  **   ****   * ***  *   ****  *     **

-1980 bp  GTTTGGTGTCATTTAATCAAACAAGCTGCTTTATTTTTCATAGTTGTGCTAATAGCTGAG      ZIP4A_AWOK01066417.1
-1949 bp  CATTTTTATAATCTAATCCAAACAGACCCTAAGTG---------------AATTAATGAA      ZIP4B_AWOK01166346.1
            **  * * ** ***** **  **   **   *                ***   ***

-1920 bp  GCGGACCCACGTTGTATCAAGTTGGTTCAACTGAACTTGTTTTGCTAATTTTTATTATTA      ZIP4A_AWOK01066417.1
-1904 bp  GTAAAACAATGTTAATTAACTATGGTTTATTGAGAAAAATTATGCAAAGCATCCAATGGT      ZIP4B_AWOK01166346.1
          *   * * * ***   * *   ***** *     *    ** *** **   *

-1860 bp  TTTATACGTGTAAATTATACTCATAAACAAATATTTAAATAAAATGAATCTACTTGGAAC      ZIP4A_AWOK01066417.1
-1844 bp  TTTGCCAGCTTGATTTA--------CTTTAATATTCAAATTACCTAAGATTACTTGATAA      ZIP4B_AWOK01166346.1
          ***    *  * * ***            ****** **** *  * *   ******  *

-1800 bp  GACGAGGAATGTTAGCTCGATGGTCAAG-TGA--GGTCATTTTAAGATATAGAACGTGGA      ZIP4A_AWOK01066417.1
-1792 bp  TGCATGAGTTTTTCTACACCAAGTGAAATTGACATATAATATAATAACTTAGAAAATAGA      ZIP4B_AWOK01166346.1
            *  *   * **         ** **  ***    * ** * *  *  *****  * **

-1743 bo  T---TCGATTAT-------ATGCTATGTTCAGACTCTTCCGTATATTTT--TAATTTCTG      ZIP4A_AWOK01066417.1
-1732 bp  GTAATAACGTATATGTCTGTTCTTATATTTAAACTCATATATAACCACTAACAATTGCCC      ZIP4B_AWOK01166346.1
              *    ***        * *** ** * **** *   **     *   **** *

-1695 bp  ----AAATCACGAG---GAATGCACATGTTTGCACAGTACA-ACTGTAACCGGCTACTCA      ZIP4A_AWOK01066417.1
-1672 bp  TAAAGGATAATGGGGGAAAAAGAAAGAAAAAGCACATACAAAGGTGTAACCT-CAAATAA      ZIP4B_AWOK01166346.1
                ** * *    ** * *       *****    *   *******  * * * *

-1643 bp  CTTTGACATCT-GACTACTATGACTATTGTTAAATTAGTTTAATCTACTAATATGTTAAA      ZIP4A_AWOK01066417.1
-1613 bp  CCTATACATTTTACCTCAAATCACA---------TTTAATTTATGTGCTTTTACATCACT      ZIP4B_AWOK01166346.1
          * *  **** *   **   ** **          **   ** ** * **  **  * *

-1584 bp  AATGTTAGATTATTTTTCGTTTTGAATTTTATTATTTGCCATTGTCATATAGAAGTTAGT      ZIP4A_AWOK01066417.1
-1562 bp  TAATAAGGATAAAT---------CAATGTGATTCGATGTACAAATGAGGATGTGGATAAA      ZIP4B_AWOK01166346.1
          *     *** * *          *** * ***   **      * *    *  * **

-1524 bp  TTTAATTGTTATCTGTTTAGATTATATTATTTATTGTTTGTAAATAACAC---TTAGCTT      ZIP4A_AWOK01066417.1
-1511 bp  TATAATTTTCAAAGGTTTGAATTCGAACGTTGAGCCTTATACTATGGGAGGGCAATCCGG
          * ***** * *   ****  ***  *   ** *   **     **   *        *

                                 RY-element
-1467 bp  TTAANGTAACACTTAACTTTTAAAAGTTATGTTTGTGTGAAATTTATTTAGTGTCGATTA      ZIP4A_AWOK01066417.1
-1451 bp  TCCACGAAATAATATTCTGCATCCATGCATGGTCCAGGGAAGGGTT------GTACCTAA      ZIP4B_AWOK01166346.1
          *  * * ** * *   **      *   *** *   * ***   *       **   * *

                                                          LTR
-1407 bp  TATTACTTGAGTACTTCCATACGTTATAAAGTTGTATTTTTTCACTTCTTCTTTTTGCAA      ZIP4A_AWOK01066417.1
-1397 bp  AGGTAGGGGTGTTCATAAAAACCCAAAAAA-TCGAA------CCAAACCGAAAATCAAAC      ZIP4B_AWOK01166346.1
             **   * ** * *  * **   * *** * * *      *    *      *   *

          circadian
-1347 bp  TTAGCTCTTTTAAAATTTATTTACTATTTTATTAAGTTGTGTGTTTATTTATTTATTGTT      ZIP4A_AWOK01066417.1
-1344 bp  CAAGCCGATCAAAAAAACCAATACTTTTTGGTTTGGTTTGGTTTTGG-------------      ZIP4B_AWOK01166346.1
            ***   *  ****      **** ***  **  ***  ** **

-1287 bp  CAAGTAAATTCTCTGTTAATTTTAATTCTTATTTTATTTATATTTATGGTGCTTCAAAAT      ZIP4A_AWOK01066417.1
-1297 bp  -------------------------------------------TTTTGAATTTTAAAAAC      ZIP4B_AWOK01166346.1
                                                     ** **    ** ****

-1227 bp  TGTTAGAATGTTATTGTATTAACTTATCTTTTGAGTAAATCATAAAAATTTA-----AAA      ZIP4A_AWOK01066417.1
-1280 bp  CGATCAAACTTG----GTTTGGTTTTGGTTTTAATCAAAAAATAACCGGAAAAAAACCGAA      ZIP4B_AWOK01166346.1
```

```
          * *  **  *       **   **   **** *  ***  ****      *      **

                                  ERE  Box-1   GT1-motif
-1172 bp  CTGAAGAATTTAAAGGAAAATTTATAGTCTAATAACGGGTTAAAAATAATATATATATAT      ZIP4A_AWOK01066417.1
-1225 bp  CCAAACCAACTAAAGGAGTAGCTATTTCAAATTT----ATTATTACACCTATAT--ATAT      ZIP4B_AWOK01166346.1
          *  **  *  *******  *  ***     * *      ***  *    *****  ****

                             Box-1       TGACG-motif
-1112 bp  ATATATTTCTATTAT--------AGACTATTATCACCTAATG-----TTTACCATA----      ZIP4A_AWOK01066417.1
-1171 bp  GTATATTTTTATACAAAGTTTCAAAATTTTATGACGAAAGTTAATCGTTTGCACTTTTA      ZIP4B_AWOK01166346.1
          ******* ***           * * * **** **  **        **  **

-1069 bp  ---T-CTTAATGAACTCTTGAATATATTCAGGGTCCGCCCCTAGCTATTAGTACTACCAG      ZIP4A_AWOK01066417.1
-1111 bp  GTATAGTTCTTTACCTTTACATTCTAGTTTG-ATTGGTAGTTTTCTTTTGTTAAGTGTAA      ZIP4B_AWOK01166346.1
             *  **  * * ** *  * * ** *  *  *    *  ** **  **     *

-1013 bp  TAGGGGGTTCCATTTTTATTATTAATTGTAGTTTTGAGACACTAGAAGATTCATTAATTT      ZIP4A_AWOK01066417.1
-1052 bp  GAATCCATTTCATGTTTAAAAAAAAATATATTTTTAATT--------GAGTCCTTAAATT      ZIP4B_AWOK01166346.1
          *     ** *** ****  *  ** * ** **** *          ** ** **** **

-953 bp   ATTCTCTGCGATGCAATTCTATATTCTATTGGATTTTTTGTTTCCTTTCTT-TTTTCCTT      ZIP4A_AWOK01066417.1
-1000 bp  ATTCATCACCATTTGATTCAATTATCATCAATATATCTTAGTAAATAATAGATTTCTCAA      ZIP4B_AWOK01166346.1
          ****    * **   **** **  **      ** * **  *   *      ***  *

-894 bp   ATGAGACGTAGCTCTTTCCCTTTTCTTTTTCATTTTCTTTCCTACTTTGTTTATGTAATA      ZIP4A_AWOK01066417.1
-940 bp   AGGACAATTGATTTGATAGTGTTACGTTGAAAATATGGTCGCCAGAATATGTGTTTGGTA      ZIP4B_AWOK01166346.1
          * ** *  *   *   *    ** * **   * * *  *  * *   * * * * *  **

                                       LTR        LTR
-834 bp   CTGTAGGTTTATTTATCTTCTATACTTTAACTATATAATTTAGAAAACTAACTAGAA---      ZIP4A_AWOK01066417.1
-880 bp   GTGTATGTCTTATATTTAAGAA-AAAACCGAAAAATAACCGAAAAAACCGACTAAACCCG      ZIP4B_AWOK01166346.1
          **** ** *  *  *     * *        * ****   * *****  **** *

               MBS
-777 bp   -TAAAACAAGTAACCATATAAAAC--------------TGATATAAATC-----------      ZIP4A_AWOK01066417.1
-821 bp   ACATTAACTGAATCGAGAAAAAACCAACTTAATTGGTTTGGTTTGATTCGAATATTTGAA      ZIP4B_AWOK01166346.1
            *  *   * * * * * *****              ** * * * **

-743 bp   TAAAAGATTTA----ATTTTACAAAATTAAACTGAAA-AGGATATAAGAGAAATTAAAGA      ZIP4A_AWOK01066417.1
-761 bp   AAACCGACTTACTTGGTTTGATTTCTTTTTAGGGAAAAACCGAACCAAACCGAACCATGA      ZIP4B_AWOK01166346.1
          **  ** ***     *** *     **  *  **** *    *  * *   *   * **

-687 bp   ATATTTTTTAAGAAAATAAAATAAAATATATATACACTTC--------------------      ZIP4A_AWOK01066417.1
-701 bp   ACACCCCTACCTAAAGGGAAATGATGTATGCAATCTATCCTGAGTATCGGTGGCTGATTC      ZIP4B_AWOK01166346.1
          * *    *    ***   **** *  ***  *  *  * *

          G-box
-647 bp   -------------TATGAACGAATATGTATGTAAAGACGTGTACAAAACCAGAGAATTAA      ZIP4A_AWOK01066417.1
-641 bp   CACGACTCGAATTCATGACCTATAGATCATGCAGAGACAACTTTATTACTGCTCCGAGAC      ZIP4B_AWOK01166346.1
              **** * *      *** * ****   *  *  **         *

                                    Skn-1 motif   ERE   CAAT-box
-600 bp   GTGCGTGCAAAATTATACATTTGTACATTTTGGTCATTTTTAATATTCAAATATTTATAA      ZIP4A_AWOK01066417.1
-581 bp   TCTCCTTAAGCTTTATACAA-TGTGAATTCGAATTAATTGCCATTTCAAATTAGATATAA      ZIP4B_AWOK01166346.1
             * *  *   *******  ***  ***    * * **   ** *  ** **  *****

                                                          MBS
-540 bp   AATAA------TAATTATTCTAATTTAATGAAGTGCTCTAATCGACCAACTGCACTAGTG      ZIP4A_AWOK01066417.1
-521 bp   AATACCTTAAAAAATAAATATTTTTACTCTGAAAATGCACATTGGCTCAGACAACTACTA      ZIP4B_AWOK01166346.1
          ****        *** * * *  **      *     *  ** * *  *    **** *

-486 bp   TTATTCAAAAAGAAAAGGAAAGGAAGGAAATATAAAGTGGAGTGCATGACTTATTACGCA      ZIP4A_AWOK01066417.1
-461 bp   ATATGTTATTAAAAAATCGAAAAAAGGAAATAAAAAGTGGAGTGCATGACTTATTACGCA      ZIP4B_AWOK01166346.1
          ***   *  * ****   **  ********* ***************************

-426 bp   GAGGTGTCGACacagtggagctcattctcttctaaaacaagcgactgtatcaaagtctcc      ZIP4A_AWOK01066417.1
-401 bp   GAGGTGTCGACACAGTGGAGCTCATTCTCTTGTAAAACAAGCgactgtatcaaagtctcc      ZIP4B_AWOK01166346.1
          ******************************* ****************************

                                       TATA-box
-366 bp   ccatctcctctctcttctttctttactaataatgtcgacatcattcccagtatcttcttc      ZIP4A_AWOK01066417.1
-341 bp   ccatctcctctctcttctttctttactaataatgtcgacatcattccctgtatcttcttc      ZIP4B_AWOK01166346.1
          ************************************************ ***********
```

```
-306 bp    catttctcatccccatctattttccccttctttcattctttctttatccttttctaaacc       ZIP4A_AWOK01066417.1
-281 bp    cttttctcgtccccatctattttccctttctttctttctttctttatccatttctaaatc       ZIP4B_AWOK01166346.1
           * ****** ***************** ******* ************** ******** *

-246 bp    ctccctagtccctccttacaacagcaagaggatttataaaacagtgttttcggacaaaag       ZIP4A_AWOK01066417.1
-221 bp    ttccctactccctcct-acaacaacaacaggatttataaaacag---------acaaaag       ZIP4B_AWOK01166346.1
            ****** ******** ****** *** ****************         *******

-186 bp    gggtttgttctcgtcaagaattattacaaaattcaccactttcttgttagattaatacta       ZIP4A_AWOK01066417.1
-171 bp    gtgtttgttctcgtcaagatttattacaaaattcaccactttcgt--------------a       ZIP4B_AWOK01166346.1
           * ***************** *********************** *              *

-126 bp    taattttggtcaagacctttcacctatatatagagatttgtgatgctttctctgtttgtg       ZIP4A_AWOK01066417.1
-125 bp    taattttggtcaagacccttcagctatatatagagatttgtgttagtgttatgctttct-       ZIP4B_AWOK01166346.1
           ***************** **** ******************* *  * *     *** *

-66 bp     taaacttgttacgtaattggaaatag-cgttttgagggacaaattccctctcttttcccc       ZIP4A_AWOK01066417.1
-66 bp     -ctgtttattacgtaattggaaatagagcttttgagggacaaattccctctcttttccac       ZIP4B_AWOK01166346.1
                ** ******************   ***************************** *

-7 bp      atatcccATG                                                         ZIP4A_AWOK01066417.1
-7 bp      atatcccATG                                                         ZIP4B_AWOK01166346.1
           **********
```

C. Comparison of the promoter sequences including 5'UTR of *NtZIP4A* from three tobacco cultivars: NT90 (AYMY01036928.1 position 1204..4716), **K326** (AWOJ01064181.1 position 22570..26069) and **Basma Xanthi** (AWOK01066417.1 position 1230..4748).

For comparison the following fragments were used: **3513 bp** (cultivar **NT90**);
3500 bp (cultivar **K326**) and **3519 bp** (cultivar **Basma Xanthi**) upstream from the START codon.
Comparison was made with the Clustal Omega https://www.ebi.ac.uk/Tools/msa/clustalo/
In blue - differences between sequences;
* depicts identical nucleotides at a given position.

```
AYMY01036928.1      CACTAATAAGAAACAGAAAAAGAAAAACAAGTTGCTGATTTTTTGTTTAAAGTTTAAGAG      60
AWOJ01064181.1      CACTAATAAGAAACAGAAAAAGAAAAACAAGTTGCTGATTTTTTGTTTAAAGTTTAAGAG      60
AWOK01066417.1      CACTAATAAGAAACAGAAAAAGAAAAACAAGTTGCTGATTTTTTGTTTAAAGTTTAAGAG      60
                    ************************************************************

AYMY01036928.1      AAGCATAAAGAAATGCGTGCAAGTTATATACAGTTAGACATGGTCAGCATAATTAACCTA      120
AWOJ01064181.1      AAGCATAAAGAAATGCGTGCAAGTTATATACAGTTAGACATGGTCAGCATAATTAACCTA      120
AWOK01066417.1      AAGCATAAAGAAATGCGTGCAAGTTATATACAGTTAGACATGGTCAGCATAATTAACCTA      120
                    ************************************************************

AYMY01036928.1      ATCAATCCTCGTTTTGGAAGAAATCAAGAATAAAATATGTTCGTTTAATATTAACCTCTT      180
AWOJ01064181.1      ATCAATCCTCGTTTTGGAAGAAATCAAGAATAAAATATGTTCGTTTAATATTAACCTCTT      180
AWOK01066417.1      ATCAATCCTCGTTTTGGAAGAAATCAAGAATAAAATATGTTCGTTTAATATTAACCTCTT      180
                    ************************************************************

AYMY01036928.1      CTTTTATCGACGAATATTCTCTGATTCTTTTCTGAACCTTTCGTTCTCTTTTTTACTGTA      240
AWOJ01064181.1      CTTTTATCGACGAATATTCTCTGATTCTTTTCTGAACCTTTCGTTCTCTTTTTTACTGTA      240
AWOK01066417.1      CTTTTATCGACGAATATTCTCTGATTCTTTTCTGAACCTTTCGTTCTCTTTTTTACTGTA      240
                    ************************************************************

AYMY01036928.1      AAAATAGTCTTATTGACGTATTTTAGTAGAAATAGTTTTGGCTGATCATTAAATTATGAA      300
AWOJ01064181.1      AAAATAGTCTTATTGACGTATTTTAGTAGAAATAGTTTTGGCTGATCATTAAATTATGAA      300
AWOK01066417.1      AAAATAGTCTTATTGACGTATTTTAGTAGAAATAGTTTTGGCTGATCATTAAATTATGAA      300
                    ************************************************************

AYMY01036928.1      ATACGTAAACTTTAATTACATATAATCGATGTGTTTGGTGAGAAATCCAACTTTGATTTT      360
AWOJ01064181.1      ATACGTAAACTTTAATTACATATAATCGATGTGTTTGGTGAGAAATCCAACTTTGATTTT      360
AWOK01066417.1      ATACGTAAACTTTAATTACATATAATCGATGTGTTTGGTGAGAAATCCAACTTTGATTTT      360
                    ************************************************************

AYMY01036928.1      GCGCCAATTAGAATAACTGTATTGGAACAAAGAAAATGTGCTTACAAATTAAAACCATAG      420
AWOJ01064181.1      GCGCCAATTAGAATAACTGTATTGGAACAAAGAAAATGTGCTTACAAATTAAAACCATAG      420
AWOK01066417.1      GCGCCAATTAGAATAACTGTATTGGAACAAAGAAAATGTGCTTACAAATTAAAACCATAG      420
                    ************************************************************

AYMY01036928.1      ATTAAGCATCCTACCATGGTACTACTATTCGTAACGAAATTTTGATAATCATTTCTTTTA      480
AWOJ01064181.1      ATTAAGCATCCTACCATGGTACTACTATTCGTAACGAAATTTTGATAATCATTTCTTTTA      480
AWOK01066417.1      ATTAAGCATCCTACCATGGTACTACTATTCGTAACGAAATTTTGATAATCATTTCTTTTA      480
                    ************************************************************

AYMY01036928.1      ATAATCGTGATGTCCAAATCAGCTTACAGACACGTAGTTTAATTTTACACGATACATGTT      540
AWOJ01064181.1      ATAATCGTGATGTCCAAATCAGCTTACAGACACGTAGTTTAATTTTACACGATACATGTT      540
AWOK01066417.1      ATAATCGTGATGTCCAAATCAGCTTACAGACACGTAGTTTAATTTTACACGATACATGTT      540
                    ************************************************************

AYMY01036928.1      ATCTCACACTAACTGATGTATCAGATAACTTTATCCATTAAAAGCTCGAACAAATTGAAA      600
AWOJ01064181.1      ATCTCACACTAACTGATGTATCAGATAACTTTATCCATTAAAAGCTCGAACAAATTGAAA      600
AWOK01066417.1      ATCTCACACTAACTGATGTATCAGATAACTTTATCCATTAAAAGCTCGAACAAATTGAAA      600
                    ************************************************************

AYMY01036928.1      ATACTCACCTACTACTATTTAAACATTTGGGTTAATATTTTGGGATAAAATTTACCCGTG      660
AWOJ01064181.1      ATACTCACCTACTACTATTTAAACATTTGGGTTAATATTTTGGGATAAAATTTACCCGTG      660
AWOK01066417.1      ATACTCACCTACTACTATTTAAACATTTGGGTTAATATTTTGGGATAAAATTTACCCGTG      660
                    ************************************************************

AYMY01036928.1      CGGCCGTACCTGCGACCAAACTATACCAACTTACATGATTAAGTGAATTAATGAAGTAAA      720
AWOJ01064181.1      CGGCCGTACCTGCGACCAAACTATACCAACTTACATGATTAAGTGAATTAATGAAGTAAA      720
AWOK01066417.1      CGGCCGTACCTGCGACCAAACTATACCAACTTACATGATTAAGTGAATTAATGAAGTAAA      720
```

```
                             ************************************************************

AYMY01036928.1      ACAATGTTAATTAACTATGGTTAATTGAGAAAAGTATGCAAAGCATCCAATGATTTTGCC      780
AWOJ01064181.1      ACAATGTTAATTAACTATGGTTAATTGAGAAAAGTATGCAAAGCATCCAATGATTTTGCC      780
AWOK01066417.1      ACAATGTTAATTAACTATGGTTAATTGAGAAAAGTATGCAAAGCATCCAATGATTTTGCC      780
                    ************************************************************

AYMY01036928.1      AGCTTGATTTACTTTAATATTCAAATTACCTAAAATTACTTGAAAATGCATGAGTTTTTC      840
AWOJ01064181.1      AGCTTGATTTACTTTAATATTCAAATTACCTAAAATTACTTGAAAATGCATGAGTTTTTC      840
AWOK01066417.1      AGCTTGATTTACTTTAATATTCAAATTACCTAAAATTACTTGAAAATGCATGAGTTTTTC      840
                    ************************************************************

AYMY01036928.1      TACACCGAGTGAAATTGACATATAATATAATAACTTAAAAAATAGAGTAATAAAGTATAT      900
AWOJ01064181.1      TACACCGAGTGAAATTGACATATAATATAATAACTTAAAAAATAGAGTAATAAAGTATAT      900
AWOK01066417.1      TACACCGAGTGAAATTGACATATAATATAATAACTTAAAAAATAGAGTAATAAAGTATAT      900
                    ************************************************************

AYMY01036928.1      ATCTGTTCTTATATTTAAACTCAATTGCCCTAAAAGATAAAGGGAAAAAAAGAGAAAGAA      960
AWOJ01064181.1      ATCTGTTCTTATATTTAAACTCAATTGCCCTAAAAGATAAAGGGAAAAAAAGAGAAAGAA      960
AWOK01066417.1      ATCTGTTCTTATATTTAAACTCAATTGCCCTAAAAGATAAAGGGAAAAAAAGAGAAAGAA      960
                    ************************************************************

AYMY01036928.1      AAAACACATGCAAAGGTGTAACCTCAAATAACCTATACATTTTACCTCAAATCACAATTA      1020
AWOJ01064181.1      AAAACACATGCAAAGGTGTAACCTCAAATAACCTATACATTTTACCTCAAATCACAATTA      1020
AWOK01066417.1      AAAACACATGCAAAGGTGTAACCTCAAATAACCTATACATTTTACCTCAAATCACAATTA      1020
                    ************************************************************

AYMY01036928.1      ATTATGTGCTTTTACATCACTTAATTAGGATAAATCAATGTAATTCGAGGTATAAATGGG      1080
AWOJ01064181.1      ATTATGTGCTTTTACATCACTTAATTAGGATAAATCAATGTAATTCGAGGTATAAATGGG      1080
AWOK01066417.1      ATTATGTGCTTTTACATCACTTAATTAGGATAAATCAATGTAATTCGAGGTATAAATGGG      1080
                    ************************************************************

AYMY01036928.1      GATTTGAATAAAATATTTTTTCACCTCTTAATTAAAGATTCTGAATTTGAACGCTGAAAA      1140
AWOJ01064181.1      GATTTGAATAAAATATTTTTTCACCTCTTAATTAAAGATTCTGAATTTGAACGCTGAAAA      1140
AWOK01066417.1      GATTTGAATAAAATATTTTTTCACCTCTTAATTAAAGATTCTGAATTTGAACGCTGAAAA      1140
                    ************************************************************

AYMY01036928.1      TAAAATAAAAAAGCTCTAATAAAGTGTATTTTTTCTTTTAATGATCCTTATACGATGAAA      1200
AWOJ01064181.1      TAAAATAAAAAAGCTCTAATAAAGTGTATTTTTTCTTTTAATGATCCTTATACGATGAAA      1200
AWOK01066417.1      TAAAATAAAAAAGCTCTAATAAAGTGTATTTTTTCTTTTAATGATCCTTATACGATGAAA      1200
                    ************************************************************

AYMY01036928.1      GGGCAGCTCGGTGCACGAAACAACATCTCGCATTCACGCAGGATCCAGGGAAGGGACGCA      1260
AWOJ01064181.1      GGGCAGCTCGGTGCACGAAACAACATCTCGCATTCACGCAGGATCCAGGGAAGGGACGCA      1260
AWOK01066417.1      GGGCAGCTCGGTGCACGAAACAACATCTCGCATTCACGCAGGATCCAGGGAAGGGACGCA      1260
                    ************************************************************

AYMY01036928.1      CCCCAATAAGTGTGCCGTAGGCAGTCTATCTTTATGAAAGTATCAATGACTGATTACACG      1320
AWOJ01064181.1      CCCCAATAAGTGTGCCGTAGGCAGTCTATCTTTATGAAAGTATCAATGACTGATTACACG      1320
AWOK01066417.1      CCCCAATAAGTGTGCCGTAGGCAGTCTATCTTTATGAAAGTATCAATGACTGATTACACG      1320
                    ************************************************************

AYMY01036928.1      GCTCAAACCCATAACCTATAAGTCACACAAAGAGACAACTTTATCGTTGCTTCAAGGCTC      1380
AWOJ01064181.1      GCTCAAACCCATAACCTATAAGTCACACAAAGAGACAACTTTATCGTTGCTTCAAGGCTC      1380
AWOK01066417.1      GCTCAAACCCATAACCTATAAGTCACACAAAGAGACAACTTTATCGTTGCTTCAAGGCTC      1380
                    ************************************************************

AYMY01036928.1      TCTTTCACCATAGGAATTTGAATAAATTGGCATTTCAAATTAGATAGTATCAAATACGGA      1440
AWOJ01064181.1      TCTTTCACCATAGGAATTTGAATAAATTGGCATTTCAAATTAGATAGTATCAAATACGGA      1440
AWOK01066417.1      TCTTTCACCATAGGAATTTGAATAAATTGGCATTTCAAATTAGATAGTATCAAATACGGA      1440
                    ************************************************************

AYMY01036928.1      ATGAGAAATTAAAAAATAAATATTTTTACTCTTTGAATGTACATTGGATCACACAACAAT      1500
AWOJ01064181.1      ATGAGAAATTAAAAAATAAATATTTTTACTCTTTGAATGTACATTGGATCACACAACAAT      1500
AWOK01066417.1      ATGAGAAATTAAAAAATAAATATTTTTACTCTTTGAATGTACATTGGATCACACAACAAT      1500
                    ************************************************************

AYMY01036928.1      GTTATCCTTATATTTTACATAAAACGCCTATTCTTTTTGTGTTTGGTGTCATTTAATCAA      1560
AWOJ01064181.1      GTTATCCTTATATTTTACATAAAACGCCTATTCTTTTTGTGTTTGGTGTCATTTAATCAA      1560
AWOK01066417.1      GTTATCCTTATATTTTACATAAAACGCCTATTCTTTTTGTGTTTGGTGTCATTTAATCAA      1560
                    ************************************************************

AYMY01036928.1      ACAAGCTGCTTTATTTTTCATAGTTGTGCTAATAGCTGAGGCGGACCCACGTTGTATCAA      1620
AWOJ01064181.1      ACAAGCTGCTTTATTTTTCATAGTTGTGCTAATAGCTGAGGCGGACCCACGTTGTATCAA      1620
AWOK01066417.1      ACAAGCTGCTTTATTTTTCATAGTTGTGCTAATAGCTGAGGCGGACCCACGTTGTATCAA      1620
                    ************************************************************
```

```
AYMY01036928.1      GTTGGTTCAACTGAACTTGTTTTGCTAATTTTTATTATTATTTATACGTGTAAATTATAC      1680
AWOJ01064181.1      GTTGGTTCAACTGAACTTGTTTTGCTAATTTTTATTATTATTTATACGTGTAAATTATAC      1680
AWOK01066417.1      GTTGGTTCAACTGAACTTGTTTTGCTAATTTTTATTATTATTTATACGTGTAAATTATAC      1680
                    ************************************************************

AYMY01036928.1      TCATAAACAAATATTTAAATAAAATGAATCTACTTGGAACGACGAGGAATGTTAGCTCGA      1740
AWOJ01064181.1      TCATAAACAAATATTTAAATAAAATGAATCTACTTGGAACGACGAGGAATGTTAGCTCGA      1740
AWOK01066417.1      TCATAAACAAATATTTAAATAAAATGAATCTACTTGGAACGACGAGGAATGTTAGCTCGA      1740
                    ************************************************************

AYMY01036928.1      TGGTCAAGTGAGGTCATTTTAAGATATAGAACGTGGATTCGATTATATGCTATGTTCA--      1798
AWOJ01064181.1      TGGTCAAGTGAGGTCATTTTAAGATATAGAACGTGGATTCGATTATATGCTATGTTCAGA      1800
AWOK01066417.1      TGGTCAAGTGAGGTCATTTTAAGATATAGAACGTGGATTCGATTATATGCTATGTTCAGA      1800
                    **********************************************************  

AYMY01036928.1      CTCTTCCGTATATTTTTAATTTCTGAAATCACGAGGAATGCACATGTTTGCACAGTACAA      1858
AWOJ01064181.1      CTCTTCCGTATATTTTTAATTTCTGAAATCACGAGGAATGCACATGTTTGCACAGTACAA      1860
AWOK01066417.1      CTCTTCCGTATATTTTTAATTTCTGAAATCACGAGGAATGCACATGTTTGCACAGTACAA      1860
                    ************************************************************

AYMY01036928.1      CTGTAACCGGCTACTCACTTTGACATCTGACTACTATGACTATTGTTAAATTAGTTTAAT      1918
AWOJ01064181.1      CTGTAACCGGCTACTCACTTTGACATCTGACTACTATGACTATTGTTAAATTAGTTTAAT      1920
AWOK01066417.1      CTGTAACCGGCTACTCACTTTGACATCTGACTACTATGACTATTGTTAAATTAGTTTAAT      1920
                    ************************************************************

AYMY01036928.1      CTACTAATATGTTAAAAATGTTAGATTATTTTTCGTTTTGAATTTTATTATTTGCCATTG      1978
AWOJ01064181.1      CTACTAATATGTTAAAAATGTTAGATTATTTTTCGTTTTGAATTTTATTATTTGCCATTG      1980
AWOK01066417.1      CTACTAATATGTTAAAAATGTTAGATTATTTTTCGTTTTGAATTTTATTATTTGCCATTG      1980
                    ************************************************************

AYMY01036928.1      TCATATAGAAGTTAGTTTTAATTGTTATCTGTTTAGATTATATTATTTATTGTTTGTAAA      2038
AWOJ01064181.1      TCATATAGAAGTTAGTTTTAATTGTTATCTGTTTAGATTATATTATTTATTGTTTGTAA-      2039
AWOK01066417.1      TCATATAGAAGTTAGTTTTAATTGTTATCTGTTTAGATTATATTATTTATTGTTTGTAAA      2040
                    *********************************************************** 

AYMY01036928.1      TAACACTTAGC------NGTAACACTTAACTTTTAAAAGTTATGTTTGTGTGAAATTTAT      2092
AWOJ01064181.1      -----------------GTAACACTTAACTTTTAAAAGTTATGTTTGTGTGAAATTTAT      2081
AWOK01066417.1      TAACACTTAGCTTTTAANGTAACACTTAACTTTTAAAAGTTATGTTTGTGTGAAATTTAT      2100
                                      ******************************************

AYMY01036928.1      TTAGTGTCGATTATATTACTTGAGTACTTCCATACGTTATAAAGTTGTATTTTTTCACTT      2152
AWOJ01064181.1      TTAGTGTCGATTATATTACTTGAGTACTTCCATACGTTATAAAGTTGTATTTTTTCACTT      2141
AWOK01066417.1      TTAGTGTCGATTATATTACTTGAGTACTTCCATACGTTATAAAGTTGTATTTTTTCACTT      2160
                    ************************************************************

AYMY01036928.1      CTTCTTTTTGCAATTAGCTCTTTTAAAATTTATTTACTATTTTATTAAGTTGTGTGTTTA      2212
AWOJ01064181.1      CTTCTTTTTGCAATTAGCTCTTTTAAAATTTATTTACTATTTTATTAAGTTGTGTGTTTA      2201
AWOK01066417.1      CTTCTTTTTGCAATTAGCTCTTTTAAAATTTATTTACTATTTTATTAAGTTGTGTGTTTA      2220
                    ************************************************************

AYMY01036928.1      TTTATTTATTGTTCAAGTAAATTCTCTGTTAATTTTAATTCTTATTTTATTTATATTTAT      2272
AWOJ01064181.1      TTTATTTATTGTTCAAGTAAATTCTCTGTTAATTTTAATTCTTATTTTATTTATATTTAT      2261
AWOK01066417.1      TTTATTTATTGTTCAAGTAAATTCTCTGTTAATTTTAATTCTTATTTTATTTATATTTAT      2280
                    ************************************************************

AYMY01036928.1      GGTGCTTCAAAATTGTTAGAATGTTATTGTATTAACTTATCTTTTGAGTAAATCATAAAA      2332
AWOJ01064181.1      GGTGCTTCAAAATTGTTAGAATGTTATTGTATTAACTTATCTTTTGAGTAAATCATAAAA      2321
AWOK01066417.1      GGTGCTTCAAAATTGTTAGAATGTTATTGTATTAACTTATCTTTTGAGTAAATCATAAAA      2340
                    ************************************************************

AYMY01036928.1      ATTTAAAACTGAAGAATTTAAAGGAAAATTTATAGTCTAATAACGGGTTAAAAATAATAT      2392
AWOJ01064181.1      ATTTAAAACTGAAGAATTTAAAGGAAAATTTATAGTCTAATAACGGGTTAAAAATAATAT      2381
AWOK01066417.1      ATTTAAAACTGAAGAATTTAAAGGAAAATTTATAGTCTAATAACGGGTTAAAAATAATAT      2400
                    ************************************************************

AYMY01036928.1      ATATATATATATATATTTCTATTATAGACTATTATCACCTAATGTTTACCATATCTTAAT      2452
AWOJ01064181.1      ATATA--TATATATATTTCTATTATAGACTATTATCACCTAATGTTTACCATATCTTAAT      2439
AWOK01066417.1      ATATA--TATATATATTTCTATTATAGACTATTATCACCTAATGTTTACCATATCTTAAT      2458
                    *****  *****************************************************

AYMY01036928.1      GAACTCTTGAATATATTCAGGGTCCGCCCCTAGCTATTAGTACTACCAGTAGGGGGTTCC      2512
AWOJ01064181.1      GAACTCTTGAATATATTCAGGGTCCGCCCCTAGCTATTAGTACTACCAGTAGGGGGTTCC      2499
AWOK01066417.1      GAACTCTTGAATATATTCAGGGTCCGCCCCTAGCTATTAGTACTACCAGTAGGGGGTTCC      2518
                    ************************************************************

AYMY01036928.1      ATTTTTATTATTAATTGTAGTTTTGAGACACTAGAAGATTCATTAATTTATTCTCTGCGA      2572
AWOJ01064181.1      ATTTTTATTATTAATTGTAGTTTTGAGACACTAGAAGATTCATTAATTTATTCTCTGCGA      2559
```

```
AWOK01066417.1      ATTTTTATTATTAATTGTAGTTTTGAGACACTAGAAGATTCATTAATTTATTCTCTGCGA      2578
                    ************************************************************

AYMY01036928.1      TGCAATTCTATATTCTATTGGATTTTTTGTTTCCTTTCTTTTTTCCTTATGAGACGTAGC      2632
AWOJ01064181.1      TGCAATTCTATATTCTATTGGATTTTTTGTTTCCTTTCTTTTTTCCTTATGAGACGTAGC      2619
AWOK01066417.1      TGCAATTCTATATTCTATTGGATTTTTTGTTTCCTTTCTTTTTTCCTTATGAGACGTAGC      2638
                    ************************************************************

AYMY01036928.1      TCTTTCCCTTTTCTTTTTCATTTTCTTTCCTACTTTGTTTATGTAATACTGTAGGTTTAT      2692
AWOJ01064181.1      TCTTTCCCTTTTCTTTTTCATTTTCTTTCCTACTTTGTTTATGTAATACTGTAGGTTTAT      2679
AWOK01066417.1      TCTTTCCCTTTTCTTTTTCATTTTCTTTCCTACTTTGTTTATGTAATACTGTAGGTTTAT      2698
                    ************************************************************

AYMY01036928.1      TTATCTTCTATACTTTAACTATATAATTTAGAAAACTAATTAGAATAAAACAAGTAACCA      2752
AWOJ01064181.1      TTATCTTCTATACTTTAACTATATAATTTAGAAAACTAATTAGAATAAAACAAGTAACCA      2739
AWOK01066417.1      TTATCTTCTATACTTTAACTATATAATTTAGAAAACTAACTAGAATAAAACAAGTAACCA      2758
                    *************************************** ********************

AYMY01036928.1      TATAAAACTGATATAAATCTAAAAGATTTAATTTTACAAAATTAAACTGAAAAGGATATA      2812
AWOJ01064181.1      TATAAAACTGATATAAATCTAAAAGATTTAATTTTACAAAATTAAACTGAAAAGGATATA      2799
AWOK01066417.1      TATAAAACTGATATAAATCTAAAAGATTTAATTTTACAAAATTAAACTGAAAAGGATATA      2818
                    ************************************************************

AYMY01036928.1      AGAGAAATTAAAGAATATTTTTTAAGAAAATAAAATAAAATATATATACACTTCTATGAA      2872
AWOJ01064181.1      AGAGAAATTAAAGAATATTTTTTAAGAAAATAAAATAAAATATATATACACTTCTATGAA      2859
AWOK01066417.1      AGAGAAATTAAAGAATATTTTTTAAGAAAATAAAATAAAATATATATACACTTCTATGAA      2878
                    ************************************************************

AYMY01036928.1      CGAATATGTATGCAAAGACGTGTACAAAACCAGAGAATTAAGTGCGTGCAAAATTATACA      2932
AWOJ01064181.1      CGAATATGTATGCAAAGACGTGTACAAAACCAGAGAATTAAGTGCGTGCAAAATTATACA      2919
AWOK01066417.1      CGAATATGTATGTAAAGACGTGTACAAAACCAGAGAATTAAGTGCGTGCAAAATTATACA      2938
                    ************ ***********************************************

AYMY01036928.1      TTTGTACATTTTGGTCATTTTTAATATTCAAATATTTATAAAATAATAATTATTCTAATT      2992
AWOJ01064181.1      TTTGTACATTTTGGTCATTTTTAATATTCAAATATTTATAAAATAATAATTATTCTAATT      2979
AWOK01066417.1      TTTGTACATTTTGGTCATTTTTAATATTCAAATATTTATAAAATAATAATTATTCTAATT      2998
                    ************************************************************

AYMY01036928.1      TAATGAAGTGCTCTAATCGACCAACTGCACTAGTGTTATTCAAAAAGAAAAGGAAAGGAA      3052
AWOJ01064181.1      TAATGAAGTGCTCTAATCGACCAACTGCACTAGTGTTATTCAAAAAGAAAAGGAAAGGAA      3039
AWOK01066417.1      TAATGAAGTGCTCTAATCGACCAACTGCACTAGTGTTATTCAAAAAGAAAAGGAAAGGAA      3058
                    ************************************************************

AYMY01036928.1      GGAAATATAAAGTGGAGTGCATGACTTATTACGCAGAGGTGTCGACACAGTGGAGCTCAT      3112
AWOJ01064181.1      GGAAATATAAAGTGGAGTGCATGACTTATTACGCAGAGGTGTCGACACAGTGGAGCTCAT      3099
AWOK01066417.1      GGAAATATAAAGTGGAGTGCATGACTTATTACGCAGAGGTGTCGACACAGTGGAGCTCAT      3118
                    ************************************************************

AYMY01036928.1      TCTCTTCTAAAACAAGCGACTGTATCAAAGTCTCCCCATCTCCTCTCTCTTCTTTCTTTA      3172
AWOJ01064181.1      TCTCTTCTAAAACAAGCGACTGTATCAAAGTCTCCCCATCTCCTCTCTCTTCTTTCTTTA      3159
AWOK01066417.1      TCTCTTCTAAAACAAGCGACTGTATCAAAGTCTCCCCATCTCCTCTCTCTTCTTTCTTTA      3178
                    ************************************************************

AYMY01036928.1      CTAATAATGTCGACATCATTCCCAGTATCTTCTTCCATTTCTCATCCCCATCTATTTTCC      3232
AWOJ01064181.1      CTAATAATGTCGACATCATTCCCAGTATCTTCTTCCATTTCTCATCCCCATCTATTTTCC      3219
AWOK01066417.1      CTAATAATGTCGACATCATTCCCAGTATCTTCTTCCATTTCTCATCCCCATCTATTTTCC      3238
                    ************************************************************

AYMY01036928.1      CCTTCTTTCATTCTTTCTTTATCCTTTTCTAAACCCTCCCTAGTCCCTCCTTACAACAGC      3292
AWOJ01064181.1      CCTTCTTTCATTCTTTCTTTATCCTTTTCTAAACCCTCCCTAGTCCCTCCTTACAACAGC      3279
AWOK01066417.1      CCTTCTTTCATTCTTTCTTTATCCTTTTCTAAACCCTCCCTAGTCCCTCCTTACAACAGC      3298
                    ************************************************************


AYMY01036928.1      AAGAGGATTTATAAAACAGTGTTTTCGGACAAAAGGGGTTTGTTCTCGTCAAGAATTATT      3352
AWOJ01064181.1      AAGAGGATTTATAAAACAGTGTTTTCGGACAAAAGGGGTTTGTTCTCGTCAAGAATTATT      3339
AWOK01066417.1      AAGAGGATTTATAAAACAGTGTTTTCGGACAAAAGGGGTTTGTTCTCGTCAAGAATTATT      3358
                    ************************************************************

AYMY01036928.1      ACAAAATTCACCACTTTCTTGTTAGATTAATACTATAATTTTGGTCAAGACCTTTCACCT      3412
AWOJ01064181.1      ACAAAATTCACCACTTTCTTGTTAGATTAATACTATAATTTTGGTCAAGACCTTTCACCT      3399
AWOK01066417.1      ACAAAATTCACCACTTTCTTGTTAGATTAATACTATAATTTTGGTCAAGACCTTTCACCT      3418
                    ************************************************************

AYMY01036928.1      ATATATAGAGATTTGTGATGCTTTCTCTGTTTGTGTAAACTTGTTACGTAATTGGAAATA      3472
AWOJ01064181.1      ATATATAGAGATTTGTGATGCTTTCTCTGTTTGTGTAAACTTGTTACGTAATTGGAAATA      3459
```

```
AWOK01066417.1      ATATATAGAGATTTGTGATGCTTTCTCTGTTTGTGTAAACTTGTTACGTAATTGGAAATA      3478
                    ************************************************************

AYMY01036928.1      GCGTTTTGAGGGACAAATTCCCTCTCTTTTCCCCATATCCC      3513
AWOJ01064181.1      GCGTTTTGAGGGACAAATTCCCTCTCTTTTCCCCATATCCC      3500
AWOK01066417.1      GCGTTTTGAGGGACAAATTCCCTCTCTTTTCCCCATATCCC      3519
                    *****************************************
```

D. Comparison of the promoter sequences including 5'UTR of *NtZIP4B* from three tobacco cultivars: NT90 (AYMY01036928.1 position 1204..4716), **K326** (AWOJ01064181.1 position 22570..26069) and **Basma Xanthi** (AWOK01066417.1 position 1230..4748).

For comparison **3500 bp** fragments upstream from the START codon were used. Comparison was made with the Clustal Omega https://www.ebi.ac.uk/Tools/msa/clustalo/
* depicts identical nucleotides at a given position.

```
gb|AYMY01065187.1      ATCATATTTCCACATAAAAGTGTTCCGAATATACGCCCAGACCGAGTACGTAAATCGAGG   60
gb|AWOJ01110029.1      ATCATATTTCCACATAAAAGTGTTCCGAATATACGCCCAGACCGAGTACGTAAATCGAGG   60
gb|AWOK01166346.1      ATCATATTTCCACATAAAAGTGTTCCGAATATACGCCCAGACCGAGTACGTAAATCGAGG   60
                       ************************************************************

gb|AYMY01065187.1      TGAGGAAAAAGGAGGCTTCTAAGGCCTCGAAACATGAAATTTACTCGTAAATCAAGTGAT   120
gb|AWOJ01110029.1      TGAGGAAAAAGGAGGCTTCTAAGGCCTCGAAACATGAAATTTACTCGTAAATCAAGTGAT   120
gb|AWOK01166346.1      TGAGGAAAAAGGAGGCTTCTAAGGCCTCGAAACATGAAATTTACTCGTAAATCAAGTGAT   120
                       ************************************************************

gb|AYMY01065187.1      GACCTTTTGGGTCATCACATTATAACTATGTGACCAATATTTAGTAAGGGTAGTTTAGTC   180
gb|AWOJ01110029.1      GACCTTTTGGGTCATCACATTATAACTATGTGACCAATATTTAGTAAGGGTAGTTTAGTC   180
gb|AWOK01166346.1      GACCTTTTGGGTCATCACATTATAACTATGTGACCAATATTTAGTAAGGGTAGTTTAGTC   180
                       ************************************************************

gb|AYMY01065187.1      ATACTAGGTATTTTTGTATAGGATTTAGCATTTTCTTAATGGGCATGCTAAAAGAAAACT   240
gb|AWOJ01110029.1      ATACTAGGTATTTTTGTATAGGATTTAGCATTTTCTTAATGGGCATGCTAAAAGAAAACT   240
gb|AWOK01166346.1      ATACTAGGTATTTTTGTATAGGATTTAGCATTTTCTTAATGGGCATGCTAAAAGAAAACT   240
                       ************************************************************

gb|AYMY01065187.1      GGTCACTTATTATGAACCGAAGGGAGTAAGTTGAAAGAAATCGCCTACTGCTATTTTTGC   300
gb|AWOJ01110029.1      GGTCACTTATTATGAACCGAAGGGAGTAAGTTGAAAGAAATCGCCTACTGCTATTTTTGC   300
gb|AWOK01166346.1      GGTCACTTATTATGAACCGAAGGGAGTAAGTTGAAAGAAATCGCCTACTGCTATTTTTGC   300
                       ************************************************************

gb|AYMY01065187.1      CTCTCCTAAAATATGAAACAGAGATCTCATGATTATTAATCCACTTAATTAAGCACTAGG   360
gb|AWOJ01110029.1      CTCTCCTAAAATATGAAACAGAGATCTCATGATTATTAATCCACTTAATTAAGCACTAGG   360
gb|AWOK01166346.1      CTCTCCTAAAATATGAAACAGAGATCTCATGATTATTAATCCACTTAATTAAGCACTAGG   360
                       ************************************************************

gb|AYMY01065187.1      TCCACTCTTAGGTGTATGTTTGACAATCAATTTTACTATAACCGAATTATCACTAGAGTT   420
gb|AWOJ01110029.1      TCCACTCTTAGGTGTATGTTTGACAATCAATTTTACTATAACCGAATTATCACTAGAGTT   420
gb|AWOK01166346.1      TCCACTCTTAGGTGTATGTTTGACAATCAATTTTACTATAACCGAATTATCACTAGAGTT   420
                       ************************************************************

gb|AYMY01065187.1      AGGGGAATACCAATCAAACTGAAAAATCTCACCAAATCGGATAGTCAAATCAAACCGATT   480
gb|AWOJ01110029.1      AGGGGAATACCAATCAAACTGAAAAATCTCACCAAATCGGATAGTCAAATCAAACCGATT   480
gb|AWOK01166346.1      AGGGGAATACCAATCAAACTGAAAAATCTCACCAAATCGGATAGTCAAATCAAACCGATT   480
                       ************************************************************

gb|AYMY01065187.1      AACAACTTGTTTGGATGGTTGTTACTCATTGTATTGTATCGTATTGTTCGATGTTTGTTT   540
gb|AWOJ01110029.1      AACAACTTGTTTGGATGGTTGTTACTCATTGTATTGTATCGTATTGTTCGATGTTTGTTT   540
gb|AWOK01166346.1      AACAACTTGTTTGGATGGTTGTTACTCATTGTATTGTATCGTATTGTTCGATGTTTGTTT   540
                       ************************************************************

gb|AYMY01065187.1      TGATTGTTACTTAAATTTATTGTATCGTACCGTTAAATCCGTCGTTACATAACGACGAAA   600
gb|AWOJ01110029.1      TGATTGTTACTTAAATTTATTGTATCGTACCGTTAAATCCGTCGTTACATAACGACGAAA   600
gb|AWOK01166346.1      TGATTGTTACTTAAATTTATTGTATCGTACCGTTAAATCCGTCGTTACATAACGACGAAA   600
                       ************************************************************

gb|AYMY01065187.1      TGTGCCACTTTATGTAACGACCTATTTGGTGTGGTCGCGTCGTTACCTTATCTTTTTCTC   660
gb|AWOJ01110029.1      TGTGCCACTTTATGTAACGACCTATTTGGTGTGGTCGCGTCGTTACCTTATCTTTTTCTC   660
gb|AWOK01166346.1      TGTGCCACTTTATGTAACGACCTATTTGGTGTGGTCGCGTCGTTACCTTATCTTTTTCTC   660
                       ************************************************************

gb|AYMY01065187.1      TCAATCTCACCCTTCATTATTATTAAATAATTTTATTTTATCATTTGCCCTATTTTTTAT   720
gb|AWOJ01110029.1      TCAATCTCACCCTTCATTATTATTAAATAATTTTATTTTATCATTTGCCCTATTTTTTAT   720
gb|AWOK01166346.1      TCAATCTCACCCTTCATTATTATTAAATAATTTTATTTTATCATTTGCCCTATTTTTTAT   720
                       ************************************************************

gb|AYMY01065187.1      ATAATAATTTTACCCTGTATCATAATTTTTTTTATAATATTGCAAGTTTATTCTTCATAT   780
gb|AWOJ01110029.1      ATAATAATTTTACCCTGTATCATAATTTTTTTTATAATATTGCAAGTTTATTCTTCATAT   780
gb|AWOK01166346.1      ATAATAATTTTACCCTGTATCATAATTTTTTTTATAATATTGCAAGTTTATTCTTCATAT   780
                       ************************************************************
```

```
gb|AYMY01065187.1      TGCTGGTGTGTGATCATGAAACGATGACAAACGATACAGTGCAATGCAATACAGTACGAT   840
gb|AWOJ01110029.1      TGCTGGTGTGTGATCATGAAACGATGACAAACGATACAGTGCAATGCAATACAGTACGAT   840
gb|AWOK01166346.1      TGCTGGTGTGTGATCATGAAACGATGACAAACGATACAGTGCAATGCAATACAGTACGAT   840
                       ************************************************************

gb|AYMY01065187.1      ACGATACACTATGAAACGATAGGTAACAATCCAAACAAGCTGTAAAAAAATTCGATAAGG   900
gb|AWOJ01110029.1      ACGATACACTATGAAACGATAGGTAACAATCCAAACAAGCTGTAAAAAAATTCGATAAGG   900
gb|AWOK01166346.1      ACGATACACTATGAAACGATAGGTAACAATCCAAACAAGCTGTAAAAAAATTCGATAAGG   900
                       ************************************************************

gb|AYMY01065187.1      TTTGGTTTGATTTGATTTGGTATTGAGTTAAAAAATCCGAACCAAACCGACATATAAATA   960
gb|AWOJ01110029.1      TTTGGTTTGATTTGATTTGGTATTGAGTTAAAAAATCCGAACCAAACCGACATATAAATA   960
gb|AWOK01166346.1      TTTGGTTTGATTTGATTTGGTATTGAGTTAAAAAATCCGAACCAAACCGACATATAAATA   960
                       ************************************************************

gb|AYMY01065187.1      TATAATTTTTTATATATACTTTTAAGACTTTTATAGAATTTTCTTTAAAAAAAAATGTCTA   1020
gb|AWOJ01110029.1      TATAATTTTTTATATATACTTTTAAGACTTTTATAGAATTTTCTTTAAAAAAAAATGTCTA   1020
gb|AWOK01166346.1      TATAATTTTTTATATATACTTTTAAGACTTTTATAGAATTTTCTTTAAAAAAAAATGTCTA   1020
                       ************************************************************

gb|AYMY01065187.1      GAAATATTTGTGATTCTCCTATGGGATGTAATATTTAGTTAAATATGAAGTGTTTCCATA   1080
gb|AWOJ01110029.1      GAAATATTTGTGATTCTCCTATGGGATGTAATATTTAGTTAAATATGAAGTGTTTCCATA   1080
gb|AWOK01166346.1      GAAATATTTGTGATTCTCCTATGGGATGTAATATTTAGTTAAATATGAAGTGTTTCCATA   1080
                       ************************************************************

gb|AYMY01065187.1      TGTATTAACTTTAAAAAGAAATCGTGCAGCTTATGTTAGTTGATTCATTGCTATTGATAA   1140
gb|AWOJ01110029.1      TGTATTAACTTTAAAAAGAAATCGTGCAGCTTATGTTAGTTGATTCATTGCTATTGATAA   1140
gb|AWOK01166346.1      TGTATTAACTTTAAAAAGAAATCGTGCAGCTTATGTTAGTTGATTCATTGCTATTGATAA   1140
                       ************************************************************

gb|AYMY01065187.1      GTCGTAACAATGAAGACCAGATGTTAACCTGTGTTATCGTACTAAAAATATCTTAAGAAA   1200
gb|AWOJ01110029.1      GTCGTAACAATGAAGACCAGATGTTAACCTGTGTTATCGTACTAAAAATATCTTAAGAAA   1200
gb|AWOK01166346.1      GTCGTAACAATGAAGACCAGATGTTAACCTGTGTTATCGTACTAAAAATATCTTAAGAAA   1200
                       ************************************************************

gb|AYMY01065187.1      ACGAAGGAGGAGAAGGATGTGGAGAAAGGGGGCTGAAGTTGTTTAAAAAGTGGTTACAAG   1260
gb|AWOJ01110029.1      ACGAAGGAGGAGAAGGATGTGGAGAAAGGGGGCTGAAGTTGTTTAAAAAGTGGTTACAAG   1260
gb|AWOK01166346.1      ACGAAGGAGGAGAAGGATGTGGAGAAAGGGGGCTGAAGTTGTTTAAAAAGTGGTTACAAG   1260
                       ************************************************************

gb|AYMY01065187.1      TTAAAACTTTTTTAAAAAATGGGTATATGTTAAATGGGGGCGACCAAATAGGGCGCCCCG   1320
gb|AWOJ01110029.1      TTAAAACTTTTTTAAAAAATGGGTATATGTTAAATGGGGGCGACCAAATAGGGCGCCCCG   1320
gb|AWOK01166346.1      TTAAAACTTTTTTAAAAAATGGGTATATGTTAAATGGGGGCGACCAAATAGGGCGCCCCG   1320
                       ************************************************************

gb|AYMY01065187.1      TGCAATTTTTACACTTGCGACCAAACTATACCAACTTACATTATTGGGTGTTTGGATTGG   1380
gb|AWOJ01110029.1      TGCAATTTTTACACTTGCGACCAAACTATACCAACTTACATTATTGGGTGTTTGGATTGG   1380
gb|AWOK01166346.1      TGCAATTTTTACACTTGCGACCAAACTATACCAACTTACATTATTGGGTGTTTGGATTGG   1380
                       ************************************************************

gb|AYMY01065187.1      TTTTTAGGCTAGTCAAATCAACTTTTAAATTCTTTTTAACCTTTTTTAGTATTTGGCAAA   1440
gb|AWOJ01110029.1      TTTTTAGGCTAGTCAAATCAACTTTTAAATTCTTTTTAACCTTTTTTAGTATTTGGCAAA   1440
gb|AWOK01166346.1      TTTTTAGGCTAGTCAAATCAACTTTTAAATTCTTTTTAACCTTTTTTAGTATTTGGCAAA   1440
                       ************************************************************

gb|AYMY01065187.1      GCTATAAAGTGCTTAAAATAAATTAAAAACTGCTTAAAACAAGCCAAAGAAACAAGCTGA   1500
gb|AWOJ01110029.1      GCTATAAAGTGCTTAAAATAAATTAAAAACTGCTTAAAACAAGCCAAAGAAACAAGCTGA   1500
gb|AWOK01166346.1      GCTATAAAGTGCTTAAAATAAATTAAAAACTGCTTAAAACAAGCCAAAGAAACAAGCTGA   1500
                       ************************************************************

gb|AYMY01065187.1      CCAATCTCAACTTATTGCTTTTTGGCTTAAAAACTATTTCTGCTGAAAAGTCATTTTTAT   1560
gb|AWOJ01110029.1      CCAATCTCAACTTATTGCTTTTTGGCTTAAAAACTATTTCTGCTGAAAAGTCATTTTTAT   1560
gb|AWOK01166346.1      CCAATCTCAACTTATTGCTTTTTGGCTTAAAAACTATTTCTGCTGAAAAGTCATTTTTAT   1560
                       ************************************************************

gb|AYMY01065187.1      AATCTAATCCAAACAGACCCTAAGTGAATTAATGAAGTAAAACAATGTTAATTAACTATG   1620
gb|AWOJ01110029.1      AATCTAATCCAAACAGACCCTAAGTGAATTAATGAAGTAAAACAATGTTAATTAACTATG   1620
gb|AWOK01166346.1      AATCTAATCCAAACAGACCCTAAGTGAATTAATGAAGTAAAACAATGTTAATTAACTATG   1620
                       ************************************************************

gb|AYMY01065187.1      GTTTATTGAGAAAAATTATGCAAAGCATCCAATGGTTTTGCCAGCTTGATTTACTTTAAT   1680
gb|AWOJ01110029.1      GTTTATTGAGAAAAATTATGCAAAGCATCCAATGGTTTTGCCAGCTTGATTTACTTTAAT   1680
gb|AWOK01166346.1      GTTTATTGAGAAAAATTATGCAAAGCATCCAATGGTTTTGCCAGCTTGATTTACTTTAAT   1680
                       ************************************************************

gb|AYMY01065187.1      ATTCAAATTACCTAAGATTACTTGATAATGCATGAGTTTTTCTACACCAAGTGAAATTGA   1740
```

```
gb|AWOJ01110029.1      ATTCAAATTACCTAAGATTACTTGATAATGCATGAGTTTTTCTACACCAAGTGAAATTGA   1740
gb|AWOK01166346.1      ATTCAAATTACCTAAGATTACTTGATAATGCATGAGTTTTTCTACACCAAGTGAAATTGA   1740
                       ************************************************************

gb|AYMY01065187.1      CATATAATATAATAACTTAGAAAATAGAGTAATAACGTATATGTCTGTTCTTATATTTAA   1800
gb|AWOJ01110029.1      CATATAATATAATAACTTAGAAAATAGAGTAATAACGTATATGTCTGTTCTTATATTTAA   1800
gb|AWOK01166346.1      CATATAATATAATAACTTAGAAAATAGAGTAATAACGTATATGTCTGTTCTTATATTTAA   1800
                       ************************************************************

gb|AYMY01065187.1      ACTCATATATAACCACTAACAATTGCCCTAAAGGATAATGGGGGAAAAAGAAAGAAAAAG   1860
gb|AWOJ01110029.1      ACTCATATATAACCACTAACAATTGCCCTAAAGGATAATGGGGGAAAAAGAAAGAAAAAG   1860
gb|AWOK01166346.1      ACTCATATATAACCACTAACAATTGCCCTAAAGGATAATGGGGGAAAAAGAAAGAAAAAG   1860
                       ************************************************************

gb|AYMY01065187.1      CACATACAAAGGTGTAACCTCAAATAACCTATACATTTTACCTCAAATCACATTTAATTT   1920
gb|AWOJ01110029.1      CACATACAAAGGTGTAACCTCAAATAACCTATACATTTTACCTCAAATCACATTTAATTT   1920
gb|AWOK01166346.1      CACATACAAAGGTGTAACCTCAAATAACCTATACATTTTACCTCAAATCACATTTAATTT   1920
                       ************************************************************

gb|AYMY01065187.1      ATGTGCTTTTACATCACTTAATAAGGATAAATCAATGTGATTCGATGTACAAATGAGGAT   1980
gb|AWOJ01110029.1      ATGTGCTTTTACATCACTTAATAAGGATAAATCAATGTGATTCGATGTACAAATGAGGAT   1980
gb|AWOK01166346.1      ATGTGCTTTTACATCACTTAATAAGGATAAATCAATGTGATTCGATGTACAAATGAGGAT   1980
                       ************************************************************

gb|AYMY01065187.1      GTGGATAAATATAATTTTCAAAGGTTTGAATTCGAACGTTGAGCCTTATACTATGGGAGG   2040
gb|AWOJ01110029.1      GTGGATAAATATAATTTTCAAAGGTTTGAATTCGAACGTTGAGCCTTATACTATGGGAGG   2040
gb|AWOK01166346.1      GTGGATAAATATAATTTTCAAAGGTTTGAATTCGAACGTTGAGCCTTATACTATGGGAGG   2040
                       ************************************************************

gb|AYMY01065187.1      GCAATCCGGTCCACGAAATAATATTCTGCATCCATGCATGGTCCAGGGAAGGGTTGTACC   2100
gb|AWOJ01110029.1      GCAATCCGGTCCACGAAATAATATTCTGCATCCATGCATGGTCCAGGGAAGGGTTGTACC   2100
gb|AWOK01166346.1      GCAATCCGGTCCACGAAATAATATTCTGCATCCATGCATGGTCCAGGGAAGGGTTGTACC   2100
                       ************************************************************

AYMY01065187.1         TAAAGGTAGGGGTGTTCATAAAAACCCAAAAAATCGAACCAAACCGAAAATCAAACCAAG   2160
gb|AWOJ01110029.1      TAAAGGTAGGGGTGTTCATAAAAACCCAAAAAATCGAACCAAACCGAAAATCAAACCAAG   2160
gb|AWOK01166346.1      TAAAGGTAGGGGTGTTCATAAAAACCCAAAAAATCGAACCAAACCGAAAATCAAACCAAG   2160
                       ************************************************************

gb|AYMY01065187.1      CCGATCAAAAAAACCAATACTTTTTGGTTTGGTTTGGTTTTGGTTTTGAATTTTAAAAAC   2220
gb|AWOJ01110029.1      CCGATCAAAAAAACCAATACTTTTTGGTTTGGTTTGGTTTTGGTTTTGAATTTTAAAAAC   2220
gb|AWOK01166346.1      CCGATCAAAAAAACCAATACTTTTTGGTTTGGTTTGGTTTTGGTTTTGAATTTTAAAAAC   2220
                       ************************************************************

gb|AYMY01065187.1      CGATCAAACTTGGTTTGGTTTTGGTTTTAATCAAAAAATAACCGGAAAAAACCGAACCAA   2280
gb|AWOJ01110029.1      CGATCAAACTTGGTTTGGTTTTGGTTTTAATCAAAAAATAACCGGAAAAAACCGAACCAA   2280
gb|AWOK01166346.1      CGATCAAACTTGGTTTGGTTTTGGTTTTAATCAAAAAATAACCGGAAAAAACCGAACCAA   2280
                       ************************************************************

gb|AYMY01065187.1      ACCAACTAAAGGAGTAGCTATTTCAAATTTATTATTACACCTATATATATGTATATTTTT   2340
gb|AWOJ01110029.1      ACCAACTAAAGGAGTAGCTATTTCAAATTTATTATTACACCTATATATATGTATATTTTT   2340
gb|AWOK01166346.1      ACCAACTAAAGGAGTAGCTATTTCAAATTTATTATTACACCTATATATATGTATATTTTT   2340
                       ************************************************************

gb|AYMY01065187.1      ATACAAAGTTTCAAAAATTTTATGACGAAAGTTAATCGTTTGCACTTTTAGTATAGTTCT   2400
gb|AWOJ01110029.1      ATACAAAGTTTCAAAAATTTTATGACGAAAGTTAATCGTTTGCACTTTTAGTATAGTTCT   2400
gb|AWOK01166346.1      ATACAAAGTTTCAAAAATTTTATGACGAAAGTTAATCGTTTGCACTTTTAGTATAGTTCT   2400
                       ************************************************************

gb|AYMY01065187.1      TTACCTTTACATTCTAGTTTGATTGGTAGTTTTCTTTTGTTAAGTGTAAGAATCCATTTC   2460
gb|AWOJ01110029.1      TTACCTTTACATTCTAGTTTGATTGGTAGTTTTCTTTTGTTAAGTGTAAGAATCCATTTC   2460
gb|AWOK01166346.1      TTACCTTTACATTCTAGTTTGATTGGTAGTTTTCTTTTGTTAAGTGTAAGAATCCATTTC   2460
                       ************************************************************

gb|AYMY01065187.1      ATGTTTAAAAAAAAATATATTTTTAATTGAGTCCTTAAATTATTCATCACCATTTGATTC   2520
gb|AWOJ01110029.1      ATGTTTAAAAAAAAATATATTTTTAATTGAGTCCTTAAATTATTCATCACCATTTGATTC   2520
gb|AWOK01166346.1      ATGTTTAAAAAAAAATATATTTTTAATTGAGTCCTTAAATTATTCATCACCATTTGATTC   2520
                       ************************************************************

gb|AYMY01065187.1      AATTATCATCAATATATCTTAGTAAATAATAGATTTCTCAAAGGACAATTGATTTGATAG   2580
gb|AWOJ01110029.1      AATTATCATCAATATATCTTAGTAAATAATAGATTTCTCAAAGGACAATTGATTTGATAG   2580
gb|AWOK01166346.1      AATTATCATCAATATATCTTAGTAAATAATAGATTTCTCAAAGGACAATTGATTTGATAG   2580
                       ************************************************************

gb|AYMY01065187.1      TGTTACGTTGAAAATATGGTCGCCAGAATATGTGTTTGGTAGTGTATGTCTTATATTTAA   2640
gb|AWOJ01110029.1      TGTTACGTTGAAAATATGGTCGCCAGAATATGTGTTTGGTAGTGTATGTCTTATATTTAA   2640
gb|AWOK01166346.1      TGTTACGTTGAAAATATGGTCGCCAGAATATGTGTTTGGTAGTGTATGTCTTATATTTAA   2640
```

```
                        ************************************************************

gb|AYMY01065187.1       GAAAAAACCGAAAAATAACCGAAAAAACCGACTAAACCCGACATTAACTGAATCGAGAAA   2700
gb|AWOJ01110029.1       GAAAAAACCGAAAAATAACCGAAAAAACCGACTAAACCCGACATTAACTGAATCGAGAAA   2700
gb|AWOK01166346.1       GAAAAAACCGAAAAATAACCGAAAAAACCGACTAAACCCGACATTAACTGAATCGAGAAA   2700
                        ************************************************************

gb|AYMY01065187.1       AAACCAACTTAATTGGTTTGGTTTGATTCGAATATTTGAAAAACCGACTTACTTGGTTTG   2760
gb|AWOJ01110029.1       AAACCAACTTAATTGGTTTGGTTTGATTCGAATATTTGAAAAACCGACTTACTTGGTTTG   2760
gb|AWOK01166346.1       AAACCAACTTAATTGGTTTGGTTTGATTCGAATATTTGAAAAACCGACTTACTTGGTTTG   2760
                        ************************************************************

gb|AYMY01065187.1       ATTTCTTTTTAGGGAAAAACCGAACCAAACCGAACCATGAACACCCCTACCTAAAGGGAA   2820
gb|AWOJ01110029.1       ATTTCTTTTTAGGGAAAAACCGAACCAAACCGAACCATGAACACCCCTACCTAAAGGGAA   2820
gb|AWOK01166346.1       ATTTCTTTTTAGGGAAAAACCGAACCAAACCGAACCATGAACACCCCTACCTAAAGGGAA   2820
                        ************************************************************

gb|AYMY01065187.1       ATGATGTATGCAATCTATCCTGAGTATCGGTGGCTGATTCCACGACTCGAATTCATGACC   2880
gb|AWOJ01110029.1       ATGATGTATGCAATCTATCCTGAGTATCGGTGGCTGATTCCACGACTCGAATTCATGACC   2880
gb|AWOK01166346.1       ATGATGTATGCAATCTATCCTGAGTATCGGTGGCTGATTCCACGACTCGAATTCATGACC   2880
                        ************************************************************

gb|AYMY01065187.1       TATAGATCATGCAGAGACAACTTTATTACTGCTCCGAGACTCTCCTTAAGCTTTATACAA   2940
gb|AWOJ01110029.1       TATAGATCATGCAGAGACAACTTTATTACTGCTCCGAGACTCTCCTTAAGCTTTATACAA   2940
gb|AWOK01166346.1       TATAGATCATGCAGAGACAACTTTATTACTGCTCCGAGACTCTCCTTAAGCTTTATACAA   2940
                        ************************************************************

gb|AYMY01065187.1       TGTGAATTCGAATTAATTGCCATTTCAAATTAGATATAAAATACCTTAAAAAATAAATAT   3000
gb|AWOJ01110029.1       TGTGAATTCGAATTAATTGCCATTTCAAATTAGATATAAAATACCTTAAAAAATAAATAT   3000
gb|AWOK01166346.1       TGTGAATTCGAATTAATTGCCATTTCAAATTAGATATAAAATACCTTAAAAAATAAATAT   3000
                        ************************************************************

gb|AYMY01065187.1       TTTTACTCTGAAAATGCACATTGGCTCAGACAACTACTAATATGTTATTAAAAAATCGAA   3060
gb|AWOJ01110029.1       TTTTACTCTGAAAATGCACATTGGCTCAGACAACTACTAATATGTTATTAAAAAATCGAA   3060
gb|AWOK01166346.1       TTTTACTCTGAAAATGCACATTGGCTCAGACAACTACTAATATGTTATTAAAAAATCGAA   3060
                        ************************************************************

gb|AYMY01065187.1       AAAAGGAAATAAAAAGTGGAGTGCATGACTTATTACGCAGAGGTGTCGACACAGTGGAGC   3120
gb|AWOJ01110029.1       AAAAGGAAATAAAAAGTGGAGTGCATGACTTATTACGCAGAGGTGTCGACACAGTGGAGC   3120
gb|AWOK01166346.1       AAAAGGAAATAAAAAGTGGAGTGCATGACTTATTACGCAGAGGTGTCGACACAGTGGAGC   3120
                        ************************************************************

gb|AYMY01065187.1       TCATTCTCTTGTAAAACAAGCGACTGTATCAAAGTCTCCCCATCTCCTCTCTCTTCTTTC   3180
gb|AWOJ01110029.1       TCATTCTCTTGTAAAACAAGCGACTGTATCAAAGTCTCCCCATCTCCTCTCTCTTCTTTC   3180
gb|AWOK01166346.1       TCATTCTCTTGTAAAACAAGCGACTGTATCAAAGTCTCCCCATCTCCTCTCTCTTCTTTC   3180
                        ************************************************************

gb|AYMY01065187.1       TTTACTAATAATGTCGACATCATTCCCTGTATCTTCTTCCTTTTCTCGTCCCCATCTATT   3240
gb|AWOJ01110029.1       TTTACTAATAATGTCGACATCATTCCCTGTATCTTCTTCCTTTTCTCGTCCCCATCTATT   3240
gb|AWOK01166346.1       TTTACTAATAATGTCGACATCATTCCCTGTATCTTCTTCCTTTTCTCGTCCCCATCTATT   3240
                        ************************************************************

AYMY01065187.1          TTCCCTTTCTTTCTTTCTTTCTTTATCCATTTCTAAATCTTCCCTACTCCCTCCTACAAC   3300
gb|AWOJ01110029.1       TTCCCTTTCTTTCTTTCTTTCTTTATCCATTTCTAAATCTTCCCTACTCCCTCCTACAAC   3300
gb|AWOK01166346.1       TTCCCTTTCTTTCTTTCTTTCTTTATCCATTTCTAAATCTTCCCTACTCCCTCCTACAAC   3300
                        ************************************************************

gb|AYMY01065187.1       AACAACAGGATTTATAAAACAGACAAAAGGTGTTTGTTCTCGTCAAGATTTATTACAAAA   3360
gb|AWOJ01110029.1       AACAACAGGATTTATAAAACAGACAAAAGGTGTTTGTTCTCGTCAAGATTTATTACAAAA   3360
gb|AWOK01166346.1       AACAACAGGATTTATAAAACAGACAAAAGGTGTTTGTTCTCGTCAAGATTTATTACAAAA   3360
                        ************************************************************

gb|AYMY01065187.1       TTCACCACTTTCGTATAATTTTGGTCAAGACCCTTCAGCTATATATAGAGATTTGTGTTA   3420
gb|AWOJ01110029.1       TTCACCACTTTCGTATAATTTTGGTCAAGACCCTTCAGCTATATATAGAGATTTGTGTTA   3420
gb|AWOK01166346.1       TTCACCACTTTCGTATAATTTTGGTCAAGACCCTTCAGCTATATATAGAGATTTGTGTTA   3420
                        ************************************************************

gb|AYMY01065187.1       GTGTTATGCTTTCTCTGTTTATTACGTAATTGGAAATAGAGCTTTTGAGGGACAAATTCC   3480
gb|AWOJ01110029.1       GTGTTATGCTTTCTCTGTTTATTACGTAATTGGAAATAGAGCTTTTGAGGGACAAATTCC   3480
gb|AWOK01166346.1       GTGTTATGCTTTCTCTGTTTATTACGTAATTGGAAATAGAGCTTTTGAGGGACAAATTCC   3480
                        ************************************************************

gb|AYMY01065187.1       CTCTCTTTTCCACATATCCC     3500
gb|AWOJ01110029.1       CTCTCTTTTCCACATATCCC     3500
gb|AWOK01166346.1       CTCTCTTTTCCACATATCCC     3500
                        ********************
```
