# Supplementary material for: Functional Analysis of NtZIP4B and Zn Status-Dependent Expression Pattern of Tobacco ZIP Genes
Source: Front Plant Sci. 2019 Jan 10;9:1984. doi: 10.3389/fpls.2018.01984 (PMC6335357; doi:10.3389/fpls.2018.01984)
Supplement: FILE S4 — Genomic sequences of NtZIP4A and NtZIP4B. [file Data_Sheet_4.PDF]

Supplementary File S4

Content:

(A) **Comparison of the genomic sequence of *NtZIP4A* and *NtZIP4B***
(B) **Comparison of the genomic seqence of <u>*ZIP4A*</u> originated from the following three tobacco cultivars: <u>NT90</u>, <u>K326</u> and <u>K326</u>**
(C) **Comparison of the genomic seqence of <u>*ZIP4B*</u> originated from the following three tobacco cultivars: <u>NT90</u>, <u>K326</u> and <u>K326</u>**

(A) Comparison of the genomic sequence of *NtZIP4A* (identified in the genomic reference sequence NW_015828584.1, 4717..8152) **and *NtZIP4B*** (identified in the contig AWOJ01110029.1, 14413..17643);
For comparison performed with the Clustal Omega https://www.ebi.ac.uk/Tools/msa/clustalo/ genomic fragments which comprised OFR with introns were used.
NtZIP4B nucleotide sequence cloned in this paper is identical with the sequence from this contig.

<u>Underlined sequences</u> indicate exons.
In red – START codon.
 * - depicts identical nucleotides at a given position.

```
NtZIP4A_NW_015828584.1          ATGTCGTTCATTGAGGTTCGTTCTTTCTTTCTTCAGTGCCCCCACACACAACCCCAAACC     60
NtZIP4B_AWOJ01110029.1          ATGTCGTTCACTGAGGTTCGTTCTTTCTTTCTTCAGTGCCCCCCCACACAACCCCAAACC     60
                                ********** ******************************** ****************

NtZIP4A_NW_015828584.1          CCTACTCCCTCTTCCTTTTTATTGTGTATTCCTTTCTCCTTTTTTTTTAATTGCCCCATT     120
NtZIP4B_AWOJ01110029.1          CCTGTTCCCTCTTCCTTTTTAT-----------------TCTCTTTTTCATTGCCCCATT     103
                                ***  *****************                 * * ***** ***********

NtZIP4A_NW_015828584.1          TGTCTTGAAAGTTGTGAGTTTTATTCTATTCTTTGTTAATTGTTAATTTCAGGATCTCGT     180
NtZIP4B_AWOJ01110029.1          TGTCTTGAAAGTTGTGAGTTTTATTCTATTCTTTGTTATTTGTTAATTTCAGGATCTCGT     163
                                ************************************** *********************

NtZIP4A_NW_015828584.1          GCCCTTCTTTTTTATGGACCCAAAATCTAGAGAAAAGACTGGGGCTTTCTCAGGTAAGTA     240
NtZIP4B_AWOJ01110029.1          GCCCTTCTTTTTTATGGACCCAAAAATTAGAGAAAAGACCGGGGCTTTCTCAGGTAAGTA     223
                                *************************  ************ ********************

NtZIP4A_NW_015828584.1          ATATTTTGTGTCACCATTTTCTTTTATTTATTTTTCTTTGTAATTGTTATGGGGAATGAG     300
NtZIP4B_AWOJ01110029.1          ATATTTTGTTACCCCATTTTCTTTTCTTTATTTTTCTTTGTAATTGTTATGGAAAATGAG     283
                                *********  * ************ **************************  ******

NtZIP4A_NW_015828584.1          GAAATTGAATCTTTAATTCTGGGTTTTGCTTGGTTTGGTTTGTAATTGATGGAGAATTCT     360
NtZIP4B_AWOJ01110029.1          GAAATTGAATCTTTACTTCTGGGTTTTGCTTGGTTTGGTTTGTAATTGATGGAGAATTCT     343
                                *************** ********************************************

NtZIP4A_NW_015828584.1          CCAAATTACTGTAGTTCTTTTTTCTTGTTTGTTTTATGTTCCTATTGGATACT-------     413
NtZIP4B_AWOJ01110029.1          CCAAATTACTGTAATTCTTTTTCTTG-TTTGTTTTATGTACCACTTCGATACTCTGTGTG     402
                                ************* ********  *  ************ **  ** ******

NtZIP4A_NW_015828584.1          -CTGTGTTGAGTCAGTATAATCACTTGCATCCTGTTTGGAAGTTCATTACTGTTTTGGAT     472
NtZIP4B_AWOJ01110029.1          ACTATGTTGAGTCAGTTTAATCACTTGCATCCAGTTTGGAAGTTCATTACTGTT------     456
                                ** ************ *************** *********************

NtZIP4A_NW_015828584.1          TTTAAATTTTGGATTATCTGTTTATTATAGTTTATAAGTAGTTCATTTTTGAGTCTTAT     532
NtZIP4B_AWOJ01110029.1          --------TTGGATTATCTGTTTATTATAGGTTATAAGTAGTTCACTTTTTGAGTCTTGT     508
                                ********************** ************** ************ *

NtZIP4A_NW_015828584.1          GATGAAAGTGGACATTCATTTTTGTATTAAGTGGTTACTTGTTTTATAATTCATTCATTA     592
NtZIP4B_AWOJ01110029.1          GATTGAAGTAGAAATTCTTTTTTGTATTAAGTGGTTTTTAGTTTTGTAATTCATTCATTC     568
                                ***  **** ** **** ******************  * ***** *************

NtZIP4A_NW_015828584.1          TCACTCACCTTTTCTATAAAAAGTTTTGAAATTCATTCATTCTCATGTTGTACTGATCTC     652
NtZIP4B_AWOJ01110029.1          TCACTCACCTTTTCTATAAACAGTTTGGAAATTCATTCATTCTCATGTTGTACTGATCTT     628
                                ******************** ***** ********************************

NtZIP4A_NW_015828584.1          GAT-TTTATGAAATGATGGGGTATTGAATTCCAAATTTGAATTGGTACTAAAGGTCCCTT     711
NtZIP4B_AWOJ01110029.1          GCTTTTTATGAAATGATGGGGTATTGAATTCCAAATTTGAATTGGTACTAAAGGTCTCTT     688
                                * * **************************************************** ***
```

```
NtZIP4A_NW_015828584.1          TTGTATTTTCTTAATATACATCTTGTCTTCATTTGCTCTGCACATTCGTTATGTCTGTTT     771
NtZIP4B_AWOJ01110029.1          TTGTATTTTCTTCGTATACGTCTTGTCTTCATTTGCTCTGCACATTCGTTATGTCTGTTT     748
                                ************  ***** ****************************************

NtZIP4A_NW_015828584.1          TGGCTGTTGAATCTCACTCATGTACTAAATCTGGGAAAGGGAAAGGAAGAA-AAAAAGTT     830
NtZIPB_AWOJ01110029.1           TGGCTGTTGAATCTCACTCATTTACTAAATCTTGGAAAGGGAAAGGAAGAAAAAAAAGTC     808
                                ********************* ********** ****************** *******

NtZIP4A_NW_015828584.1          AGCTTTAGTTTTACTTTGTCAAAATGTAGTTCTTGCCTAAGGTTGCGTACACCCTGAATT     890
NtZIP4B_AWOJ01110029.1          AGCTTTAGTTTTACTTTGTCAAAATGGAGTTCTTGCCTAAGGTTGCGTACACCCTGAATT     868
                                ************************** *********************************

NtZIP4A_NW_015828584.1          ACGTAGCCGACTCTTTTTTATCTATTTTCATGTATTAGGTGGAAAAGTACAGATTCCTTT     950
NtZIP4B_AWOJ01110029.1          ACGTAGCGAACTCTTTTTTATCTATTTTCATGTATTAGGTGGAAAAGTACAGATTCCTTT     928
                                *******  ***************************************************

NtZIP4A_NW_015828584.1          GACTTCCATTTTCTAATGAATTTCATGTTTCATATCAAAATATTTGAAATGTAGGGAAAC     1010
NtZIP4B_AWOJ01110029.1          GACTTCCATTTTCTAATGAA-TTCATGTTTCATATCAAGATATTTGAAATGTAGGGAAAC     987
                                ******************** ***************** *********************

NtZIP4A_NW_015828584.1          TTTGGTTAGTGAGGAGAATTCTTGTAGTGTTATTTTAAGGAATAAACAGAAAGAAATTTG     1070
NtZIP4B_AWOJ01110029.1          TTTTGTTAATGAGGAGAATTCTTGTAGTGTGGTTTTAAGGAATAAACAGAAAGAAATTTG     1047
                                *** **** *********************  ****************************

NtZIP4A_NW_015828584.1          ATAGTTCTTTATTGGTTCAATACCTAAATTGTGTAGAAATTGAAATGATTCTTACACTCT     1130
NtZIP4B_AWOJ01110029.1          ATAGTTCTTTATTGGTTCAATACCTAAATTGTGTAGACATTTAAATGATTCCGACACTCT     1107
                                ************************************* *** *********  *******

NtZIP4A_NW_015828584.1          CTTACTGTTCCTTTTGATCAAAACAGATACCATTATGCTGAAACTTTATCAATCTGTTTC     1190
NtZIP4B_AWOJ01110029.1          CTTACTGTTCCTT-TGGTCAAAACAGATACCGTTATGCTGAAACTTTATCAATCTGTTTC     1166
                                ************* ** ************** ****************************

NtZIP4A_NW_015828584.1          CAATACCACCTGTGGCAGTGCTGATGAAGAAATAGAAGGCTGCCGAGACAGCTCGGCTGC     1250
NtZIP4B_AWOJ01110029.1          CAATATTACCTGCGGCAGTGCTGATGAAGAGATAGAAGGCTGCCGAGACAGCTCGGCTGC     1226
                                *****  ***** ***************** *****************************

NtZIP4A_NW_015828584.1          TCTCACCCTTAAAATTGTGGCTATCTCTGCCATCCTCATAGCTAGCACTTGCGGAGTTGG     1310
NtZIP4B_AWOJ01110029.1          TCTTACCCTTAAAATCGTGGCTATCTCTGCCATCCTAATAGCTAGTACTTGCGGAGTTGG     1286
                                *** *********** ******************** ******** **************

NtZIP4A_NW_015828584.1          TATTCCATTAGTTGGCAAGAAGCATCGGTTCCTCCGAACTGACTCCAATCTCTTTCTTAC     1370
NtZIP4B_AWOJ01110029.1          TATCCCGTTAGTTGGCAAGAAGCATCGGTTCCTCCGAACTGACTCTAATCTCTTTCTTGC     1346
                                *** ** ************************************** ************ *

NtZIP4A_NW_015828584.1          TGTTAAAGCCTTTGCTGCTGGTGTCATCCTCTCTACAGGATTTGTCCACATATTACCAGG     1430
NtZIP4B_AWOJ01110029.1          TGTTAAAGCCTTTGCTGCTGGTGTCATCCTCTCTACAGGCTTTGTCCACATATTACCAGG     1406
                                *************************************** ********************

NtZIP4A_NW_015828584.1          CGCCACCTCATCATTAACTAATCCTTGCCTTCCGAAATTTCCTTGGTTGAAATTCCCTTT     1490
NtZIPB_AWOJ01110029.1           CGCCACCTCATCATTAACTAATCCTTGTCTTCCGAAATCTCCTTGGTTGAAATTCCCTTT     1466
                                *************************** ********** *********************

NtZIP4A_NW_015828584.1          TGCTGGTTTTATTGCTATGATGGCTGCATTGGCTACGTTGGTGGTTGACTTTGTTGGGAC     1550
NtZIP4B_AWOJ01110029.1          CGCTGGTTTTATCGCCATGATGGCTGCATTGACTACCTTGGTGGTTGACTTTGTTGGGAC     1526
                                *********** ** *************** **** ***********************

NtZIP4A_NW_015828584.1          TCAGTATTATGAGAGGAAGCAAGAGAAACAAAGCCAAAAAGATCAGATTGATTCAGTGGA     1610
NtZIP4B_AWOJ01110029.1          TCAGTATTATGAGAGGAAGCAAGAGAAACAAAGCCAAAAAGATCAGATTGATTCAGTGGA     1586
                                ************************************************************

NtZIP4A_NW_015828584.1          TTTGGTGTCAGAATCAGCTATTGTACCGGTTGAACCAAAGGCAAGGAATGAGAAATTGTT     1670
NtZIP4B_AWOJ01110029.1          TTTGGTGTCAGAATCAGCTATTGTACCAGTTGAACCAAAGGCAGGGAATGAGAAATTGTT     1646
                                *************************** *************** ****************

NtZIP4A_NW_015828584.1          TGGTGAAGAAGACGGTGGTGCAATACACATTGTTGGGATGCATGCACATGCAGCTCATCA     1730
NtZIPB_AWOJ01110029.1           TGGTGAAGAAGATGGTGGTGCAATACACATTGTTGGGATGCATGCACATGCAGCTCATCA     1706
                                ************ ***********************************************

NtZIP4A_NW_015828584.1          CAGACATAGCCATTCACAAGAACAAGGGGCATGTCAAGGGAACGTGAGGGAGCATTCCCA     1790
NtZIP4B_AWOJ01110029.1          CAGACATAGCCATTCACAAGAACAAGGGGCATGTCAAGGGAACGTGAGGGAGCATTCCCA     1766
                                ************************************************************

NtZIP4A_NW_015828584.1          TGGTCATTCGCACTCCCATAGCTTTGGTGGTGGAGATGAGGAAGGTGGAGGGAGGCATGT     1850
NtZIP4B_AWOJ01110029.1          TGGTCATTCGCACTCCCATAGCTTTGGTGGTGGAGATGAGGAAGGTGGAGGGAGGCATGT     1826
                                ************************************************************

NtZIP4A_NW_015828584.1          TGTTGTTTCTCAGGTAATTTTACTTGCATATTTTTACATGTCTTAGGCCTTTCTCTTTCT     1910
NtZIP4B_AWOJ01110029.1          TGTTGTTTCTCAGGTAATTTTACTTGCATATTTTTACATGTCTTAGGCCTTTCTCTGTAC     1886
                                ******************************************************** *

NtZIP4A_NW_015828584.1          CTGTACTTCTCCACATAAGTTCGTTTTGGTTGTATTGGGTAAATGTTGTGTCTAGAAGCA     1970
NtZIP4B_AWOJ01110029.1          T------TCTCCACTTAAGTTCGTTCT---------------------------------     1907
                                ******* ********** *

NtZIP4A_NW_015828584.1          ACAACTCAGTATAATCCCACTAGTGGGGTCTGGGGAGGCTAGTTTGTACGCAGACCTTAC     2030
NtZIP4B_AWOJ01110029.1          ------------------------------------------------------------     1907
```

```
NtZIP4A_NW_015828584.1          CCATACCCTAGGGTAGAGAGGCTGTTTCCGATAGACTCTCGGCTCCCTCCAAGAACTCTC      2090
NtZIP4B_AWOJ01110029.1          ------------------------------------------------------------      1907


NtZIP4A_NW_015828584.1          CACCTTGCTCTTGGGGTGACTCGAACTCACAACCTCTTGGTTGGAAGTGGATGGTGCTCA      2150
NtZIP4B_AWOJ01110029.1          ------------------------------------------------------------      1907


NtZIP4A_NW_015828584.1          CCACTAGAGCAACTCACTCTTGTCTAGAAGGTTGGGTGAAGGTTGGGTAAATGTTGTGTC      2210
NtZIP4B_AWOJ01110029.1          -----------------------------------GGTTGGATTGGGTAAATGTTGTGTC      1932
                                                                   *    * ******************

NtZIP4A_NW_015828584.1          TAGAAGGTTTTTGATTATCTCATTGGTTGTAGGTTTTAAAGATATGAATTACATTTCCTC      2270
NtZIP4B_AWOJ01110029.1          TAGAAGGTTTTTGATTATCTCATTGGTTGTAGGTTTTAAAGATATGAATTACATTTTCTC      1992
                                ******************************************************** ***

NtZIP4A_NW_015828584.1          GATGTCAGGATTTATGAGAAAGAAAACCTTTGGGCTTCATATGTATCATGCTGGTAAACT      2330
NtZIP4B_AWOJ01110029.1          GATGTCAGGATTTATGAGAAAGAAAATGTTTGGGCTTCATATATATCATGCTGGTAAACT      2052
                                **************************  ************** *****************

NtZIP4A_NW_015828584.1          TGATATACAATTTTCTGAACGACTTGTAGTATGTATGGTTTACCAAATTTACTACCACTT      2390
NtZIP4B_AWOJ01110029.1          TGATACAGAATTTTCTGAACGACTTGTAGTATGAATGGTTTACCAAATTTACTA---CTT      2109
                                ***** * ************************* ********************   ***

NtZIP4A_NW_015828584.1          ACAGGTTTTTGCGGAATTAATATAGAGAATTCATATAGCCAACTCTTAGGGATTGAGACA      2450
NtZIP4B_AWOJ01110029.1          ATAGGTTTTTGCGGAATTAATATAGAGAATTCATATAACCAACTCTTAGGGATTGAGACA      2169
                                * *********************************** **********************

NtZIP4A_NW_015828584.1          TAGTTGATTGATTGCAACTGCTTATACGCTACTGATTCCTTGAACTTTGATAAATACATT      2510
NtZIP4B_AWOJ01110029.1          TAGTTGATTGATTGCAACTGCTTATATGCTACTGATTCCTTGAGCTTTGATAAATGCATT      2229
                                ************************** **************** *********** ****

NtZIP4A_NW_015828584.1          TCTATAAGCTTCTGCGGAATTACACTATTTCATTTTTTTCTAGGTTTAGCTAGATATTCT      2570
NtZIP4B_AWOJ01110029.1          TCTATAAGCGTATGCGGAATTACACTATTTCATTTTCTTCTAGGTTTAGCTAGATATTCT      2289
                                ********* * ************************ ***********************

NtZIP4A_NW_015828584.1          GCTAAATTACTTTCTAATTGTTTGGTGTTTAAGATTTTTCACCCAACCTTTGAGATACTG      2630
NtZIP4B_AWOJ01110029.1          GCTAAATTATTTTCTAATTGTTTGGTGTTTAAGATTTTTCA-CCAACCTTTGAGATACTG      2348
                                ********* ******************************* ******************

NtZIP4A_NW_015828584.1          CATATATTCTCATATGAAATTTAACTTCATATAGAACATTACCTAAAGTTTGTTAGCCGT      2690
NtZIP4B_AWOJ01110029.1          CATATATTGTAATATGAAATCTAACTTCACGTAGAATATTACCTTAAGTTTGCTAGCCAT      2408
                                ******** * ********* ********  ***** ******* ******* ***** *

NtZIP4A_NW_015828584.1          TCATTGGTCTATCAGGTGCCCATCATTCGATATTTTGGTGTGGTGTGCGTCCAGG-TGAG      2749
NtZIP4B_AWOJ01110029.1          TCGTTGGCCTATAGGGTGCCCATCGTTAGATATTTTGGTGTGGTGTGTGTCAGTTCACAG      2468
                                ** **** ****  ********** ** ******************* ***       **

NtZIP4A_NW_015828584.1          AGTGGTTTATGCATTCACAGATTAGGGGTTTGTGTAAGGTTTCTCATAA---TAGATACT      2806
NtZIP4B_AWOJ01110029.1          AATGGTTTATGCATTCACAGACTAGGGATTTGCTTATGGTTTCTCATAATAGCAGATTCT      2528
                                * ******************* ***** ****  ** ************    **** **

NtZIP4A_NW_015828584.1          TGGGGGCGCTTGCTTCTTTAATGTATTACAAGACCGGAACCAACTTTTTACTTTTGTTTA      2866
NtZIP4B_AWOJ01110029.1          TGGGGGAGCTTGTTTCTTTAATGTATTACAAGACCGGAACCAACTTTTTACTTTTGTTTA      2588
                                ****** ***** ***********************************************

NtZIP4A_NW_015828584.1          ATTGGCAAAAGAGCAAAAGTT---------------TGATTTTCCCCGAGTTTGTGAAGA      2911
NtZIP4B_AWOJ01110029.1          ATTGGCAAATGAGCAAAAGTTTCATTACTCAAAGTTTGATTTTCCCCGTGTTTGTGAAGA      2648
                                ********* ***********               ************ ***********

NtZIP4A_NW_015828584.1          TACAGTGTTTGTTTATACCTGTCAGTTTGATTGTGAAAAACTTGGTCATTACAACGACTT      2971
NtZIP4B_AWOJ01110029.1          TACATTGTTTGTTTATACCTGTCAGTTTGATTGTG--AAACTTGGTCATTATAACGACTT      2706
                                **** ******************************  ************** ********

NtZIP4A_NW_015828584.1          CCTTGTATGCAGGTCTTGGAGCTGGGGATAGTATCACATTCTCTCATAATAGGCATAGCA      3031
NtZIP4B_AWOJ01110029.1          TCTTGTATGCAGGTCTTGGAGCTGGGAATAGTATCACATTCTCTCATAATAGGCATAGCA      2766
                                ************************* *********************************

NtZIP4A_NW_015828584.1          TTGGGTGTTTCAGAAAGTCCGTGCACAATTAGACCCTTGCTTGTGGCCTTGTCGTTCCAC      3091
NtZIP4B_AWOJ01110029.1          TTGGGTGTTTCAGAAAGTCCATGCACAATTAGACCCTTGCTCGTGGCCTTATCGTTCCAC      2826
                                ******************** ******************** ******** *********

NtZIP4A_NW_015828584.1          CAGTTCTTCGAAGGTTTTGCGTTAGGAGGCTGCATCTCACAGGCACAGTTCAATTCCCTC      3151
NtZIP4B_AWOJ01110029.1          CAGTTCTTCGAAGGTTTTGCGTTAGGAGGTTGCATCTCGCAGGCACAGTTCAATTCCCTC      2886
                                ***************************** ******** *********************

NtZIP4A_NW_015828584.1          CGTTCCACTATAATGGCAACGTTTTTCGCCGTAACAACACCCTTGGGAATTGCTATAGGG      3211
NtZIP4B_AWOJ01110029.1          CGTTCCACTATAATGGCAACGTTTTTCGCCGTAACAACACCCTTGGGAATTGCTATAGGA      2946
                                ***********************************************************

NtZIP4A_NW_015828584.1          ATTCTAGCTTCTTCGTCGTACAATCCACATAGCCCAAGAGCTTTGGTGGTGGAAGGGATC      3271
NtZIP4B_AWOJ01110029.1          ATTCTAGCTTCTTCATCTTACAATCCACATAGCCCAAGAGCTTTGGTAGTGGAAGGGAGC      3006
                                ************** ** ***************************** ********** *

NtZIP4A_NW_015828584.1          CTAAACTCTATATCTTCTGGAATTCTAATCTACATGGCTTTAGTAGACCTAATTGCTGCA      3331
NtZIP4B_AWOJ01110029.1          CTTAACTCTATATCTGCTGGAATTTTAATCTACATGGCTTTAGTAGACCTAATTGCTGCA      3066
                                ** ************ ******** ***********************************
```

```
NtZIP4A_NW_015828584.1          GATTTCTTGAGTAAGAGAATGAGCTGCAATACAAGGCTTCAAATAGTATCTTATTTTGCA     3391
NtZIP4B_AWOJ01110029.1          GATTTCTTGAGTAAAAGAATGAGCTGCAATACAAGGCTTCAAATAGTATCTTATTTTGCA     3126
                                ************** *********************************************

NtZIP4A_NW_015828584.1          CTATTCTTAGGGGCTGGACTCATGTCCCTTCTTGCAATATGGGCA         3436
NtZIP4B_AWOJ01110029.1          CTATTCTTAGGGGCTGGACTCATGTCCCTTCTTGCAATATGGGCA         3171
                                *********************************************
```

Percent identity   - 94.62

Percent identity was determined with the Clustal Omega https://www.ebi.ac.uk/Tools/msa/clustalo/

Comparison of the length of exons and introns of *NtZIP4A* and *NtZIP4B*

	Numbers of nucleotides							
	exon 1	Intron 1	Exon 2	Intron 2	Exon 3	Intron 3	Exon 4	ORF+introns
NtZIP4A	15	157	61	923	707	1120	453	3436
NtZIP4B	15	140	61	916	707	879	453	3171

(B) Comparison of the genomic seqence of *ZIP4A* originated from the following three tobacco cultivars: NT90 (NW_015828584.1, 4717..8152), **K326** Nitab4.5_0003621 (NCAA01001367.1, 134310..130875) and **K326** (AWOJ01064181.1, 26070.29619), performed with the Clustal Omega https://www.ebi.ac.uk/Tools/msa/clustalo/;
Basma Xanthi variety was not used for comparison due to the partial sequence only of *ZIP4A* present within one contig.

Positions of genomic fragments used for comparison (which included exons and introns) are given below above the beginning of each sequence.
Position of exons was determined based on the information on the reference gene LOC107821537 (https://www.ncbi.nlm.nih.gov/gene/?term=LOC107821537).

Underlined in red – indicates exons.
In red - START and STOP codons.
Number above the START codon indicates position of the first base of the coding sequence.
Number above the last codon before the STOP codon indicates position of the last base of the coding sequence
In blue - insertion in the intron between the 3rd and 4th exon in the sequence.
AWOJ01064181.1 as compared with the other sequences used for comparison.
* - depicts identical nucleotides at a given position.

```
                         4714
NW_015828584.1           ATGTCGTTCATTGAGGTTCGTTCTTTCTTTCTTCAGTGCCCCCACACACAACCCCAAACC      60
                         134310
NCAA01001367.1           ATGTCGTTCATTGAGGTTCGTTCTTTCTTTCTTCAGTGCCCCCACACACAACCCCAAACC      60
                         26070
AWOJ01064181.1           ATGTCGTTCATTGAGGTTCGTTCTTTCTTTCTTCAGTGCCCCCACACACAACCCCAAACC      60
                         ************************************************************

NW_015828584.1           CCTACTCCCTCTTCCTTTTTATTGTGTATTCCTTTCTCCTTTTTTTTTAATTGCCCCATT      120
NCAA01001367.1           CCTACTCCCTCTTCCTTTTTATTGTGTATTCCTTTCTCCTTTTTTTTTAATTGCCCCATT      120
AWOJ01064181.1           CCTACTCCCTCTTCCTTTTTATTGTGTATTCCTTTCTCCTTTTTTTTTAATTGCCCCATT      120
                         ************************************************************

NW_015828584.1           TGTCTTGAAAGTTGTGAGTTTTATTCTATTCTTTGTTAATTGTTAATTTCAGGATCTCGT      180
NCAA01001367.1           TGTCTTGAAAGTTGTGAGTTTTATTCTATTCTTTGTTAATTGTTAATTTCAGGATCTCGT      180
AWOJ01064181.1           TGTCTTGAAAGTTGTGAGTTTTATTCTATTCTTTGTTAATTGTTAATTTCAGGATCTCGT      180
                         ************************************************************

NW_015828584.1           GCCCTTCTTTTTTATGGACCCAAAATCTAGAGAAAAGACTGGGGCTTTCTCAGGTAAGTA      240
NCAA01001367.1           GCCCTTCTTTTTTATGGACCCAAAATCTAGAGAAAAGACTGGGGCTTTCTCAGGTAAGTA      240
AWOJ01064181.1           GCCCTTCTTTTTTATGGACCCAAAATCTAGAGAAAAGACTGGGGCTTTCTCAGGTAAGTA      240
                         ************************************************************

NW_015828584.1           ATATTTTGTGTCACCATTTTCTTTTATTTATTTTTCTTTGTAATTGTTATGGGGAATGAG      300
NCAA01001367.1           ATATTTTGTGTCACCATTTTCTTTTATTTATTTTTCTTTGTAATTGTTATGGGGAATGAG      300
AWOJ01064181.1           ATATTTTGTGTCACCATTTTCTTTTATTTATTTTTCTTTGTAATTGTTATGGGGAATGAG      300
                         ************************************************************

NW_015828584.1           GAAATTGAATCTTTAATTCTGGGTTTTGCTTGGTTTGGTTTGTAATTGATGGAGAATTCT      360
NCAA01001367.1           GAAATTGAATCTTTAATTCTGGGTTTTGCTTGGTTTGGTTTGTAATTGATGGAGAATTCT      360
AWOJ01064181.1           GAAATTGAATCTTTAATTCTGGGTTTTGCTTGGTTTGGTTTGTAATTGATGGAGAATTCT      360
                         ************************************************************

NW_015828584.1           CCAAATTACTGTAGTTCTTTTTTCTTGTTTGTTTTATGTTCCTATTGGATACTCTGTGTT      420
NCAA01001367.1           CCAAATTACTGTAGTTCTTTTTTCTTGTTTGTTTTATGTTCCTATTGGATACTCTGTGTT      420
AWOJ01064181.1           CCAAATTACTGTAGTTCTTTTTTCTTGTTTGTTTTATGTTCCTATTGGATACTCTGTGTT      420
                         ************************************************************

NW_015828584.1           GAGTCAGTATAATCACTTGCATCCTGTTTGGAAGTTCATTACTGTTTTGGATTTTAAATT      480
NCAA01001367.1           GAGTCAGTATAATCACTTGCATCCTGTTTGGAAGTTCATTACTGTTTTGGATTTTAAATT      480
AWOJ01064181.1           GAGTCAGTATAATCACTTGCATCCTGTTTGGAAGTTCATTACTGTTTTGGATTTTAAATT      480
                         ************************************************************

NW_015828584.1           TTGGATTATCTGTTTATTATAGTTTATAAGTAGTTCATTTTTTGAGTCTTATGATGAAAG      540
NCAA01001367.1           TTGGATTATCTGTTTATTATAGTTTATAAGTAGTTCATTTTTTGAGTCTTATGATGAAAG      540
```

```
AWOJ01064181.1      TTGGATTATCTGTTTATTATAGTTTATAAGTAGTTCATTTTTTGAGTCTTATGATGAAAG      540
                    ************************************************************

NW_015828584.1      TGGACATTCATTTTTGTATTAAGTGGTTACTTGTTTTATAATTCATTCATTATCACTCAC      600
NCAA01001367.1      TGGACATTCATTTTTGTATTAAGTGGTTACTTGTTTTATAATTCATTCATTATCACTCAC      600
AWOJ01064181.1      TGGACATTCATTTTTGTATTAAGTGGTTACTTGTTTTATAATTCATTCATTATCACTCAC      600
                    ************************************************************

NW_015828584.1      CTTTTCTATAAAAAGTTTTGAAATTCATTCATTCTCATGTTGTACTGATCTCGATTTTAT      660
NCAA01001367.1      CTTTTCTATAAAAAGTTTTGAAATTCATTCATTCTCATGTTGTACTGATCTCGATTTTAT      660
AWOJ01064181.1      CTTTTCTATAAAAAGTTTTGAAATTCATTCATTCTCATGTTGTACTGATCTCGATTTTAT      660
                    ************************************************************

NW_015828584.1      GAAATGATGGGGTATTGAATTCCAAATTTGAATTGGTACTAAAGGTCCCTTTTGTATTTT      720
NCAA01001367.1      GAAATGATGGGGTATTGAATTCCAAATTTGAATTGGTACTAAAGGTCCCTTTTGTATTTT      720
AWOJ01064181.1      GAAATGATGGGGTATTGAATTCCAAATTTGAATTGGTACTAAAGGTCCCTTTTGTATTTT      720
                    ************************************************************

NW_015828584.1      CTTAATATACATCTTGTCTTCATTTGCTCTGCACATTCGTTATGTCTGTTTTGGCTGTTG      780
NCAA01001367.1      CTTAATATACATCTTGTCTTCATTTGCTCTGCACATTCGTTATGTCTGTTTTGGCTGTTG      780
AWOJ01064181.1      CTTAATATACATCTTGTCTTCATTTGCTCTGCACATTCGTTATGTCTGTTTTGGCTGTTG      780
                    ************************************************************

NW_015828584.1      AATCTCACTCATGTACTAAATCTGGGAAAGGGAAAGGAAGAAAAAAAGTTAGCTTTAGTT      840
NCAA01001367.1      AATCTCACTCATGTACTAAATCTGGGAAAGGGAAAGGAAGAAAAAAAGTTAGCTTTAGTT      840
AWOJ01064181.1      AATCTCACTCATGTACTAAATCTGGGAAAGGGAAAGGAAGAAAAAAAGTTAGCTTTAGTT      840
                    ************************************************************

NW_015828584.1      TTACTTTGTCAAAATGTAGTTCTTGCCTAAGGTTGCGTACACCCTGAATTACGTAGCCGA      900
NCAA01001367.1      TTACTTTGTCAAAATGTAGTTCTTGCCTAAGGTTGCGTACACCCTGAATTACGTAGCCGA      900
AWOJ01064181.1      TTACTTTGTCAAAATGTAGTTCTTGCCTAAGGTTGCGTACACCCTGAATTACGTAGCCGA      900
                    ************************************************************

NW_015828584.1      CTCTTTTTTATCTATTTTCATGTATTAGGTGGAAAAGTACAGATTCCTTTGACTTCCATT      960
NCAA01001367.1      CTCTTTTTTATCTATTTTCATGTATTAGGTGGAAAAGTACAGATTCCTTTGACTTCCATT      960
AWOJ01064181.1      CTCTTTTTTATCTATTTTCATGTATTAGGTGGAAAAGTACAGATTCCTTTGACTTCCATT      960
                    ************************************************************

NW_015828584.1      TTCTAATGAATTTCATGTTTCATATCAAAATATTTGAAATGTAGGGAAACTTTGGTTAGT      1020
NCAA01001367.1      TTCTAATGAATTTCATGTTTCATATCAAAATATTTGAAATGTAGGGAAACTTTGGTTAGT      1020
AWOJ01064181.1      TTCTAATGAATTTCATGTTTCATATCAAAATATTTGAAATGTAGGGAAACTTTGGTTAGT      1020
                    ************************************************************

NW_015828584.1      GAGGAGAATTCTTGTAGTGTTATTTTAAGGAATAAACAGAAAGAAATTTGATAGTTCTTT      1080
NCAA01001367.1      GAGGAGAATTCTTGTAGTGTTATTTTAAGGAATAAACAGAAAGAAATTTGATAGTTCTTT      1080
AWOJ01064181.1      GAGGAGAATTCTTGTAGTGTTATTTTAAGGAATAAACAGAAAGAAATTTGATAGTTCTTT      1080
                    ************************************************************

NW_015828584.1      ATTGGTTCAATACCTAAATTGTGTAGAAATTGAAATGATTCTTACACTCTCTTACTGTTC      1140
NCAA01001367.1      ATTGGTTCAATACCTAAATTGTGTAGAAATTGAAATGATTCTTACACTCTCTTACTGTTC      1140
AWOJ01064181.1      ATTGGTTCAATACCTAAATTGTGTAGAAATTGAAATGATTCTTACACTCTCTTACTGTTC      1140
                    ************************************************************

NW_015828584.1      CTTTTGATCAAAACAGATACCATTATGCTGAAACTTTATCAATCTGTTTCCAATACCACC      1200
NCAA01001367.1      CTTTTGATCAAAACAGATACCATTATGCTGAAACTTTATCAATCTGTTTCCAATACCACC      1200
AWOJ01064181.1      CTTTTGATCAAAACAGATACCATTATGCTGAAACTTTATCAATCTGTTTCCAATACCACC      1200
                    ************************************************************

NW_015828584.1      TGTGGCAGTGCTGATGAAGAAATAGAAGGCTGCCGAGACAGCTCGGCTGCTCTCACCCTT      1260
NCAA01001367.1      TGTGGCAGTGCTGATGAAGAAATAGAAGGCTGCCGAGACAGCTCGGCTGCTCTCACCCTT      1260
AWOJ01064181.1      TGTGGCAGTGCTGATGAAGAAATAGAAGGCTGCCGAGACAGCTCGGCTGCTCTCACCCTT      1260
                    ************************************************************

NW_015828584.1      AAAATTGTGGCTATCTCTGCCATCCTCATAGCTAGCACTTGCGGAGTTGGTATTCCATTA      1320
NCAA01001367.1      AAAATTGTGGCTATCTCTGCCATCCTCATAGCTAGCACTTGCGGAGTTGGTATTCCATTA      1320
AWOJ01064181.1      AAAATTGTGGCTATCTCTGCCATCCTCATAGCTAGCACTTGCGGAGTTGGTATTCCATTA      1320
                    ************************************************************

NW_015828584.1      GTTGGCAAGAAGCATCGGTTCCTCCGAACTGACTCCAATCTCTTTCTTACTGTTAAAGCC      1380
NCAA01001367.1      GTTGGCAAGAAGCATCGGTTCCTCCGAACTGACTCCAATCTCTTTCTTACTGTTAAAGCC      1380
AWOJ01064181.1      GTTGGCAAGAAGCATCGGTTCCTCCGAACTGACTCCAATCTCTTTCTTACTGTTAAAGCC      1380
                    ************************************************************

NW_015828584.1      TTTGCTGCTGGTGTCATCCTCTCTACAGGATTTGTCCACATATTACCAGGCGCCACCTCA      1440
NCAA01001367.1      TTTGCTGCTGGTGTCATCCTCTCTACAGGATTTGTCCACATATTACCAGGCGCCACCTCA      1440
AWOJ01064181.1      TTTGCTGCTGGTGTCATCCTCTCTACAGGATTTGTCCACATATTACCAGGCGCCACCTCA      1440
                    ************************************************************
```

```
NW_015828584.1      TCATTAACTAATCCTTGCCTTCCGAAATTTCCTTGGTTGAAATTCCCTTTTGCTGGTTTT      1500
NCAA01001367.1      TCATTAACTAATCCTTGCCTTCCGAAATTTCCTTGGTTGAAATTCCCTTTTGCTGGTTTT      1500
AWOJ01064181.1      TCATTAACTAATCCTTGCCTTCCGAAATTTCCTTGGTTGAAATTCCCTTTTGCTGGTTTT      1500
                    ************************************************************

NW_015828584.1      ATTGCTATGATGGCTGCATTGGCTACGTTGGTGGTTGACTTTGTTGGGACTCAGTATTAT      1560
NCAA01001367.1      ATTGCTATGATGGCTGCATTGGCTACGTTGGTGGTTGACTTTGTTGGGACTCAGTATTAT      1560
AWOJ01064181.1      ATTGCTATGATGGCTGCATTGGCTACGTTGGTGGTTGACTTTGTTGGGACTCAGTATTAT      1560
                    ************************************************************

NW_015828584.1      GAGAGGAAGCAAGAGAAACAAAGCCAAAAAGATCAGATTGATTCAGTGGATTTGGTGTCA      1620
NCAA01001367.1      GAGAGGAAGCAAGAGAAACAAAGCCAAAAAGATCAGATTGATTCAGTGGATTTGGTGTCA      1620
AWOJ01064181.1      GAGAGGAAGCAAGAGAAACAAAGCCAAAAAGATCAGATTGATTCAGTGGATTTGGTGTCA      1620
                    ************************************************************

NW_015828584.1      GAATCAGCTATTGTACCGGTTGAACCAAAGGCAAGGAATGAGAAATTGTTTGGTGAAGAA      1680
NCAA01001367.1      GAATCAGCTATTGTACCGGTTGAACCAAAGGCAAGGAATGAGAAATTGTTTGGTGAAGAA      1680
AWOJ01064181.1      GAATCAGCTATTGTACCGGTTGAACCAAAGGCAAGGAATGAGAAATTGTTTGGTGAAGAA      1680
                    ************************************************************

NW_015828584.1      GACGGTGGTGCAATACACATTGTTGGGATGCATGCACATGCAGCTCATCACAGACATAGC      1740
NCAA01001367.1      GACGGTGGTGCAATACACATTGTTGGGATGCATGCACATGCAGCTCATCACAGACATAGC      1740
AWOJ01064181.1      GACGGTGGTGCAATACACATTGTTGGGATGCATGCACATGCAGCTCATCACAGACATAGC      1740
                    ************************************************************

NW_015828584.1      CATTCACAAGAACAAGGGGCATGTCAAGGGAACGTGAGGGAGCATTCCCATGGTCATTCG      1800
NCAA01001367.1      CATTCACAAGAACAAGGGGCATGTCAAGGGAACGTGAGGGAGCATTCCCATGGTCATTCG      1800
AWOJ01064181.1      CATTCACAAGAACAAGGGGCATGTCAAGGGAACGTGAGGGAGCATTCCCATGGTCATTCG      1800
                    ************************************************************

NW_015828584.1      CACTCCCATAGCTTTGGTGGTGGAGATGAGGAAGGTGGAGGGAGGCATGTTGTTGTTTCT      1860
NCAA01001367.1      CACTCCCATAGCTTTGGTGGTGGAGATGAGGAAGGTGGAGGGAGGCATGTTGTTGTTTCT      1860
AWOJ01064181.1      CACTCCCATAGCTTTGGTGGTGGAGATGAGGAAGGTGGAGGGAGGCATGTTGTTGTTTCT      1860
                    ************************************************************

NW_015828584.1      CAGGTAATTTTACTTGCATATTTTTACATGTCTTAGGCCTTTCTCTT-------------      1907
NCAA01001367.1      CAGGTAATTTTACTTGCATATTTTTACATGTCTTAGGCCTTTCTCTT-------------      1907
AWOJ01064181.1      CAGGTAATTTTACTTGCATATTTTTACATGTCTTAGGCCTTTCTCTGTACTTCTCCACTT      1920
                    **********************************************

NW_015828584.1      ------------------------------------------------------------      1907
NCAA01001367.1      ------------------------------------------------------------      1907
AWOJ01064181.1      AAGTTCGTTCTGGTTGGATTGGGTAAATGTTGTGTCTAGAAGGTTTTTGATTATCTCATT      1980

NW_015828584.1      -----------------------------------------TCTCTGTACTTCTCCACAT      1926
NCAA01001367.1      -----------------------------------------TCTCTGTACTTCTCCACAT      1926
AWOJ01064181.1      GGTTGTAGGTTTTAAAGATATGAATTACATTTTCTCGATNTTCTCTGTACTTCTCCACAT      2040
                                                             *******************

NW_015828584.1      AAGTTCGTTTTGGTTGTATTGGGTAAATGTTGTGTCTAGAAGCAACAACTCAGTATAATC      1986
NCAA01001367.1      AAGTTCGTTTTGGTTGTATTGGGTAAATGTTGTGTCTAGAAGCAACAACTCAGTATAATC      1986
AWOJ01064181.1      AAGTTCGTTTTGGTTGTATTGGGTAAATGTTGTGTCTAGAAGCAACAACTCAGTATAATC      2100
                    ************************************************************

NW_015828584.1      CCACTAGTGGGGTCTGGGGAGGCTAGTTTGTACGCAGACCTTACCCATACCCTAGGGTAG      2046
NCAA01001367.1      CCACTAGTGGGGTCTGGGGAGGCTAGTTTGTACGCAGACCTTACCCATACCCTAGGGTAG      2046
AWOJ01064181.1      CCACTAGTGGGGTCTGGGGAGGCTAGTTTGTACGCAGACCTTACCCATACCCTAGGGTAG      2160
                    ************************************************************

NW_015828584.1      AGAGGCTGTTTCCGATAGACTCTCGGCTCCCTCCAAGAACTCTCCACCTTGCTCTTGGGG      2106
NCAA01001367.1      AGAGGCTGTTTCCGATAGACTCTCGGCTCCCTCCAAGAACTCTCCACCTTGCTCTTGGGG      2106
AWOJ01064181.1      AGAGGCTGTTTCCGATAGACTCTCGGCTCCCTCCAAGAACTCTCCACCTTGCTCTTGGGG      2220
                    ************************************************************

NW_015828584.1      TGACTCGAACTCACAACCTCTTGGTTGGAAGTGGATGGTGCTCACCACTAGAGCAACTCA      2166
NCAA01001367.1      TGACTCGAACTCACAACCTCTTGGTTGGAAGTGGATGGTGCTCACCACTAGAGCAACTCA      2166
AWOJ01064181.1      TGACTCGAACTCACAACCTCTTGGTTGGAAGTGGATGGTGCTCACCACTAGAGCAACTCA      2280
                    ************************************************************

NW_015828584.1      CTCTTGTCTAGAAGGTTGGGTGAAGGTTGGGTAAATGTTGTGTCTAGAAGGTTTTTGATT      2226
NCAA01001367.1      CTCTTGTCTAGAAGGTTGGGTGAAGGTTGGGTAAATGTTGTGTCTAGAAGGTTTTTGATT      2226
AWOJ01064181.1      CTCTTGTCTAGAAGGTTGGGTGAAGGTTGGGTAAATGTTGTGTCTAGAAGGTTTTTGATT      2340
                    ************************************************************

NW_015828584.1      ATCTCATTGGTTGTAGGTTTTAAAGATATGAATTACATTTCCTCGATGTCAGGATTTATG      2286
```

```
NCAA01001367.1      ATCTCATTGGTTGTAGGTTTTAAAGATATGAATTACATTTCCTCGATGTCAGGATTTATG       2286
AWOJ01064181.1      ATCTCATTGGTTGTAGGTTTTAAAGATATGAATTACATTTCCTCGATGTCAGGATTTATG       2400
                    ************************************************************

NW_015828584.1      AGAAAGAAAACCTTTGGGCTTCATATGTATCATGCTGGTAAACTTGATATACAATTTTCT       2346
NCAA01001367.1      AGAAAGAAAACCTTTGGGCTTCATATGTATCATGCTGGTAAACTTGATATACAATTTTCT       2346
AWOJ01064181.1      AGAAAGAAAACCTTTGGGCTTCATATGTATCATGCTGGTAAACTTGATATACAATTTTCT       2460
                    ************************************************************

NW_015828584.1      GAACGACTTGTAGTATGTATGGTTTACCAAATTTACTACCACTTACAGGTTTTTGCGGAA       2406
NCAA01001367.1      GAACGACTTGTAGTATGTATGGTTTACCAAATTTACTACCACTTACAGGTTTTTGCGGAA       2406
AWOJ01064181.1      GAACGACTTGTAGTATGTATGGTTTACCAAATTTACTACCACTTACAGGTTTTTGCGGAA       2520
                    ************************************************************

NW_015828584.1      TTAATATAGAGAATTCATATAGCCAACTCTTAGGGATTGAGACATAGTTGATTGATTGCA       2466
NCAA01001367.1      TTAATATAGAGAATTCATATAGCCAACTCTTAGGGATTGAGACATAGTTGATTGATTGCA       2466
AWOJ01064181.1      TTAATATAGAGAATTCATATAGCCAACTCTTAGGGATTGAGACATAGTTGATTGATTGCA       2580
                    ************************************************************

NW_015828584.1      ACTGCTTATACGCTACTGATTCCTTGAACTTTGATAAATACATTTCTATAAGCTTCTGCG       2526
NCAA01001367.1      ACTGCTTATACGCTACTGATTCCTTGAACTTTGATAAATACATTTCTATAAGCTTCTGCG       2526
AWOJ01064181.1      ACTGCTTATACGCTACTGATTCCTTGAACTTTGATAAATACATTTCTATAAGCTTCTGCG       2640
                    ************************************************************

NW_015828584.1      GAATTACACTATTTCATTTTTTTCTAGGTTTAGCTAGATATTCTGCTAAATTACTTTCTA       2586
NCAA01001367.1      GAATTACACTATTTCATTTTTTTCTAGGTTTAGCTAGATATTCTGCTAAATTACTTTCTA       2586
AWOJ01064181.1      GAATTACACTATTTCATTTTTTTCTAGGTTTAGCTAGATATTCTGCTAAATTACTTTCTA       2700
                    ************************************************************

NW_015828584.1      ATTGTTTGGTGTTTAAGATTTTTCACCCAACCTTTGAGATACTGCATATATTCTCATATG       2646
NCAA01001367.1      ATTGTTTGGTGTTTAAGATTTTTCACCCAACCTTTGAGATACTGCATATATTCTCATATG       2646
AWOJ01064181.1      ATTGTTTGGTGTTTAAGATTTTTCACCCAACCTTTGAGATACTGCATATATTCTCATATG       2760
                    ************************************************************

NW_015828584.1      AAATTTAACTTCATATAGAACATTACCTAAAGTTTGTTAGCCGTTCATTGGTCTATCAGG       2706
NCAA01001367.1      AAATTTAACTTCATATAGAACATTACCTAAAGTTTGTTAGCCGTTCATTGGTCTATCAGG       2706
AWOJ01064181.1      AAATTTAACTTCATATAGAACATTACCTAAAGTTTGTTAGCCGTTCATTGGTCTATCAGG       2820
                    ************************************************************

NW_015828584.1      TGCCCATCATTCGATATTTTGGTGTGGTGTGCGTCCAGGTGAGAGTGGTTTATGCATTCA       2766
NCAA01001367.1      TGCCCATCATTCGATATTTTGGTGTGGTGTGCGTCCAGGTGAGAGTGGTTTATGCATTCA       2766
AWOJ01064181.1      TGCCCATCATTCGATATTTTGGTGTGGTGTGCGTCCAGGTGAGAGTGGTTTATGCATTCA       2880
                    ************************************************************

NW_015828584.1      CAGATTAGGGGTTTGTGTAAGGTTTCTCATAATAGATACTTGGGGGCGCTTGCTTCTTTA       2826
NCAA01001367.1      CAGATTAGGGGTTTGTGTAAGGTTTCTCATAATAGATACTTGGGGGCGCTTGCTTCTTTA       2826
AWOJ01064181.1      CAGATTAGGGGTTTGTGTAAGGTTTCTCATAATAGATACTTGGGGGCGCTTGCTTCTTTA       2940
                    ************************************************************

NW_015828584.1      ATGTATTACAAGACCGGAACCAACTTTTTACTTTTGTTTAATTGGCAAAAGAGCAAAAGT       2886
NCAA01001367.1      ATGTATTACAAGACCGGAACCAACTTTTTACTTTTGTTTAATTGGCAAAAGAGCAAAAGT       2886
AWOJ01064181.1      ATGTATTACAAGACCGGAACCAACTTTTTACTTTTGTTTAATTGGCAAAAGAGCAAAAGT       3000
                    ************************************************************

NW_015828584.1      TTGATTTTCCCCGAGTTTGTGAAGATACAGTGTTTGTTTATACCTGTCAGTTTGATTGTG       2946
NCAA01001367.1      TTGATTTTCCCCGAGTTTGTGAAGATACAGTGTTTGTTTATACCTGTCAGTTTGATTGTG       2946
AWOJ01064181.1      TTGATTTTCCCCGAGTTTGTGAAGATACAGTGTTTGTTTATACCTGTCAGTTTGATTGTG       3060
                    ************************************************************

NW_015828584.1      AAAAACTTGGTCATTACAACGACTTCCTTGTATGCAGGTCTTGGAGCTGGGGATAGTATC       3006
NCAA01001367.1      AAAAACTTGGTCATTACAACGACTTCCTTGTATGCAGGTCTTGGAGCTGGGGATAGTATC       3006
AWOJ01064181.1      AAAAACTTGGTCATTACAACGACTTCCTTGTATGCAGGTCTTGGAGCTGGGGATAGTATC       3120
                    ************************************************************

NW_015828584.1      ACATTCTCTCATAATAGGCATAGCATTGGGTGTTTCAGAAAGTCCGTGCACAATTAGACC       3066
NCAA01001367.1      ACATTCTCTCATAATAGGCATAGCATTGGGTGTTTCAGAAAGTCCGTGCACAATTAGACC       3066
AWOJ01064181.1      ACATTCTCTCATAATAGGCATAGCATTGGGTGTTTCAGAAAGTCCGTGCACAATTAGACC       3180
                    ************************************************************

NW_015828584.1      CTTGCTTGTGGCCTTGTCGTTCCACCAGTTCTTCGAAGGTTTTGCGTTAGGAGGCTGCAT       3126
NCAA01001367.1      CTTGCTTGTGGCCTTGTCGTTCCACCAGTTCTTCGAAGGTTTTGCGTTAGGAGGCTGCAT       3126
AWOJ01064181.1      CTTGCTTGTGGCCTTGTCGTTCCACCAGTTCTTCGAAGGTTTTGCGTTAGGAGGCTGCAT       3240
                    ************************************************************

NW_015828584.1      CTCACAGGCACAGTTCAATTCCCTCCGTTCCACTATAATGGCAACGTTTTTCGCCGTAAC       3186
NCAA01001367.1      CTCACAGGCACAGTTCAATTCCCTCCGTTCCACTATAATGGCAACGTTTTTCGCCGTAAC       3186
AWOJ01064181.1      CTCACAGGCACAGTTCAATTCCCTCCGTTCCACTATAATGGCAACGTTTTTCGCCGTAAC       3300
```

```
                     ************************************************************

NW_015828584.1       AACACCCTTGGGAATTGCTATAGGGATTCTAGCTTCTTCGTCGTACAATCCACATAGCCC      3246
NCAA01001367.1       AACACCCTTGGGAATTGCTATAGGGATTCTAGCTTCTTCGTCGTACAATCCACATAGCCC      3246
AWOJ01064181.1       AACACCCTTGGGAATTGCTATAGGGATTCTAGCTTCTTCGTCGTACAATCCACATAGCCC      3360
                     ************************************************************

NW_015828584.1       AAGAGCTTTGGTGGTGGAAGGGATCCTAAACTCTATATCTTCTGGAATTCTAATCTACAT      3306
NCAA01001367.1       AAGAGCTTTGGTGGTGGAAGGGATCCTAAACTCTATATCTTCTGGAATTCTAATCTACAT      3306
AWOJ01064181.1       AAGAGCTTTGGTGGTGGAAGGGATCCTAAACTCTATATCTTCTGGAATTCTAATCTACAT      3420
                     ************************************************************

NW_015828584.1       GGCTTTAGTAGACCTAATTGCTGCAGATTTCTTGAGTAAGAGAATGAGCTGCAATACAAG      3366
NCAA01001367.1       GGCTTTAGTAGACCTAATTGCTGCAGATTTCTTGAGTAAGAGAATGAGCTGCAATACAAG      3366
AWOJ01064181.1       GGCTTTAGTAGACCTAATTGCTGCAGATTTCTTGAGTAAGAGAATGAGCTGCAATACAAG      3480
                     ************************************************************

NW_015828584.1       GCTTCAAATAGTATCTTATTTTGCACTATTCTTAGGGGCTGGACTCATGTCCCTTCTTGC      3426
NCAA01001367.1       GCTTCAAATAGTATCTTATTTTGCACTATTCTTAGGGGCTGGACTCATGTCCCTTCTTGC      3426
AWOJ01064181.1       GCTTCAAATAGTATCTTATTTTGCACTATTCTTAGGGGCTGGACTCATGTCCCTTCTTGC      3540
                     ************************************************************

                           8152
NW_015828584.1       AATATGGGCATGA     3439
                         130875
NCAA01001367.1       AATATGGGCATGA     3439
                          29619
AWOJ01064181.1       AATATGGGCATGA     3553
                     *************
```

(C) Comparison of the genomic seqence of *ZIP4B* originated from the following three tobacco cultivars: NT90 (NW_015828584.1, 4717..8152), **K326** Nitab4.5_0003621 (NCAA01001367.1, 134310..130875) **and K326** (AWOJ01064181.1, 26070.29619), performed with the Clustal Omega https://www.ebi.ac.uk/Tools/msa/clustalo/;
Basma Xanthi variety was not used for comparison due to the partial sequence only of *ZIP4A* present within one contig.

Positions of genomic fragments used for comparison (which included exons and introns) are given below above the beginning of each sequence.
Position of exons was determined based on the information on the reference gene LOC107821537 (https://www.ncbi.nlm.nih.gov/gene/?term=LOC107821537).

Underlined in red – indicates exons.
In red - START and STOP codons.
Number above the START codon indicates position of the first base of the coding sequence.
Number above the last codon before the STOP codon indicates position of the last base of the coding sequence.
In blue - base which is different in three tobacco varieties.
* - depicts identical nucleotides at a given position.

```
                    83290
NW_015887304.1      ATGTCGTTCACTGAGGTTCGTTCTTTCTTTCTTCAGTGCCCCCCCACACAACCCCAAACC      60
                    99664
NCAA01003621.1      ATGTCGTTCACTGAGGTTCGTTCTTTCTTTCTTCAGTGCCCCCCCACACAACCCCAAACC      60
                    14413
AWOJ01110029.1      ATGTCGTTCACTGAGGTTCGTTCTTTCTTTCTTCAGTGCCCCCCCACACAACCCCAAACC      60
                    ************************************************************

NW_015887304.1      CCTGTTCCCTCTTCCTTTTTATTCTCTTTTTCATTGCCCCATTTGTCTTGAAAGTTGTGA      120
NCAA01003621.1      CCTGTTCCCTCTTCCTTTTTATTCTCTTTTTCATTGCCCCATTTGTCTTGAAAGTTGTGA      120
AWOJ01110029.1      CCTGTTCCCTCTTCCTTTTTATTCTCTTTTTCATTGCCCCATTTGTCTTGAAAGTTGTGA      120
                    ************************************************************

NW_015887304.1      GTTTTATTCTATTCTTTGTTATTTGTTAATTTCAGGATCTCGTGCCCTTCTTTTTTATGG      180
NCAA01003621.1      GTTTTATTCTATTCTTTGTTATTTGTTAATTTCAGGATCTCGTGCCCTTCTTTTTTATGG      180
AWOJ01110029.1      GTTTTATTCTATTCTTTGTTATTTGTTAATTTCAGGATCTCGTGCCCTTCTTTTTTATGG      180
                    ************************************************************

NW_015887304.1      ACCCAAAAATTAGAGAAAAGACCGGGGCTTTCTCAGGTAAGTAATATTTTGTTACCCCAT      240
NCAA01003621.1      ACCCAAAAATTAGAGAAAAGACCGGGGCTTTCTCAGGTAAGTAATATTTTGTTACCCCAT      240
AWOJ01110029.1      ACCCAAAAATTAGAGAAAAGACCGGGGCTTTCTCAGGTAAGTAATATTTTGTTACCCCAT      240
                    ************************************************************

NW_015887304.1      TTTCTTTTCTTTATTTTTCTTTGTAATTGTTATGGAAAATGAGGAAATTGAATCTTTACT      300
NCAA01003621.1      TTTCTTTTCTTTATTTTTCTTTGTAATTGTTATGGAAAATGAGGAAATTGAATCTTTACT      300
AWOJ01110029.1      TTTCTTTTCTTTATTTTTCTTTGTAATTGTTATGGAAAATGAGGAAATTGAATCTTTACT      300
                    ************************************************************

NW_015887304.1      TCTGGGTTTTGCTTGGTTTGGTTTGTAATTGATGGAGAATTCTCCAAATTACTGTAATTC      360
NCAA01003621.1      TCTGGGTTTTGCTTGGTTTGGTTTGTAATTGATGGAGAATTCTCCAAATTACTGTAATTC      360
AWOJ01110029.1      TCTGGGTTTTGCTTGGTTTGGTTTGTAATTGATGGAGAATTCTCCAAATTACTGTAATTC      360
                    ************************************************************

NW_015887304.1      TTTTTCTTGTTTGTTTTATGTACCACTTCGATACTCTGTGTGACTATGTTGAGTCAGTTT      420
NCAA01003621.1      TTTTTCTTGTTTGTTTTATGTACCACTTCGATACTCTGTGTGACTATGTTGAGTCAGTTT      420
AWOJ01110029.1      TTTTTCTTGTTTGTTTTATGTACCACTTCGATACTCTGTGTGACTATGTTGAGTCAGTTT      420
                    ************************************************************

NW_015887304.1      AATCACTTGCATCCAGTTTGGAAGTTCATTACTGTTTTGGATTATCTGTTTATTATAGGT      480
NCAA01003621.1      AATCACTTGCATCCAGTTTGGAAGTTCATTACTGTTTTGGATTATCTGTTTATTATAGGT      480
AWOJ01110029.1      AATCACTTGCATCCAGTTTGGAAGTTCATTACTGTTTTGGATTATCTGTTTATTATAGGT      480
                    ************************************************************

NW_015887304.1      TATAAGTAGTTCACTTTTTGAGTCTTGTGATTGAAGTAGAAATTCTTTTTTGTATTAAGT      540
NCAA01003621.1      TATAAGTAGTTCACTTTTTGAGTCTTGTGATTGAAGTAGAAATTCTTTTTTGTATTAAGT      540
AWOJ01110029.1      TATAAGTAGTTCACTTTTTGAGTCTTGTGATTGAAGTAGAAATTCTTTTTTGTATTAAGT      540
                    ************************************************************
```

```
NW_015887304.1      GGTTTTTAGTTTTGTAATTCATTCATTCTCACTCACCTTTTCTATAAACAGTTTGGAAAT      600
NCAA01003621.1      GGTTTTTAGTTTTGTAATTCATTCATTCTCACTCACCTTTTCTATAAACAGTTTGGAAAT      600
AWOJ01110029.1      GGTTTTTAGTTTTGTAATTCATTCATTCTCACTCACCTTTTCTATAAACAGTTTGGAAAT      600
                    ************************************************************

NW_015887304.1      TCATTCATTCTCATGTTGTACTGATCTTGCTTTTTATGAAATGATGGGGTATTGAATTCC      660
NCAA01003621.1      TCATTCATTCTCATGTTGTACTGATCTTGCTTTTTATGAAATGATGGGGTATTGAATTCC      660
AWOJ01110029.1      TCATTCATTCTCATGTTGTACTGATCTTGCTTTTTATGAAATGATGGGGTATTGAATTCC      660
                    ************************************************************

NW_015887304.1      AAATTTGAATTGGTACTAAAGGTCTCTTTTGTATTTTCTTCGTATACGTCTTGTCTTCAT      720
NCAA01003621.1      AAATTTGAATTGGTACTAAAGGTCTCTTTTGTATTTTCTTCGTATACGTCTTGTCTTCAT      720
AWOJ01110029.1      AAATTTGAATTGGTACTAAAGGTCTCTTTTGTATTTTCTTCGTATACGTCTTGTCTTCAT      720
                    ************************************************************

NW_015887304.1      TTGCTCTGCACATTCGTTATGTCTGTTTTGGCTGTTGAATCTCACTCATTTACTAAATCT      780
NCAA01003621.1      TTGCTCTGCACATTCGTTATGTCTGTTTTGGCTGTTGAATCTCACTCATTTACTAAATCT      780
AWOJ01110029.1      TTGCTCTGCACATTCGTTATGTCTGTTTTGGCTGTTGAATCTCACTCATTTACTAAATCT      780
                    ************************************************************

NW_015887304.1      TGGAAAGGGAAAGGAAGAAAAAAAAGTCAGCTTTAGTTTTACTTTGTCAAAATGGAGTTC      840
NCAA01003621.1      TGGAAAGGGAAAGGAAGAAAAAAAAGTCAGCTTTAGTTTTACTTTGTCAAAATGGAGTTC      840
AWOJ01110029.1      TGGAAAGGGAAAGGAAGAAAAAAAAGTCAGCTTTAGTTTTACTTTGTCAAAATGGAGTTC      840
                    ************************************************************

NW_015887304.1      TTGCCTAAGGTTGCGTACACCCTGAATTACGTAGCGAACTCTTTTTTATCTATTTTCATG      900
NCAA01003621.1      TTGCCTAAGGTTGCGTACACCCTGAATTACGTAGCGAACTCTTTTTTATCTATTTTCATG      900
AWOJ01110029.1      TTGCCTAAGGTTGCGTACACCCTGAATTACGTAGCGAACTCTTTTTTATCTATTTTCATG      900
                    ************************************************************

NW_015887304.1      TATTAGGTGGAAAAGTACAGATTCCTTTGACTTCCATTTTCTAATGAATTCATGTTTCAT      960
NCAA01003621.1      TATTAGGTGGAAAAGTACAGATTCCTTTGACTTCCATTTTCTAATGAATTCATGTTTCAT      960
AWOJ01110029.1      TATTAGGTGGAAAAGTACAGATTCCTTTGACTTCCATTTTCTAATGAATTCATGTTTCAT      960
                    ************************************************************

NW_015887304.1      ATCAAGATATTTGAAATGTAGGGAAACTTTTGTTAATGAGGAGAATTCTTGTAGTGTGGT      1020
NCAA01003621.1      ATCAAGATATTTGAAATGTAGGGAAACTTTTGTTAATGAGGAGAATTCTTGTAGTGTGGT      1020
AWOJ01110029.1      ATCAAGATATTTGAAATGTAGGGAAACTTTTGTTAATGAGGAGAATTCTTGTAGTGTGGT      1020
                    ************************************************************

NW_015887304.1      TTTAAGGAATAAACAGAAAGAAATTTGATAGTTCTTTATTGGTTCAATACCTAAATTGTG      1080
NCAA01003621.1      TTTAAGGAATAAACAGAAAGAAATTTGATAGTTCTTTATTGGTTCAATACCTAAATTGTG      1080
AWOJ01110029.1      TTTAAGGAATAAACAGAAAGAAATTTGATAGTTCTTTATTGGTTCAATACCTAAATTGTG      1080
                    ************************************************************

NW_015887304.1      TAGACATTTAAATGATTCCGACACTCTCTTACTGTTCCTTTGGTCAAAACAGATACCGTT      1140
NCAA01003621.1      TAGACATTTAAATGATTCCGACACTCTCTTACTGTTCCTTTGGTCAAAACAGATACCGTT      1140
AWOJ01110029.1      TAGACATTTAAATGATTCCGACACTCTCTTACTGTTCCTTTGGTCAAAACAGATACCGTT      1140
                    ************************************************************

NW_015887304.1      ATGCTGAAACTTTATCAATCTGTTTCCAATATTACCTGCGGCAGTGCTGATGAAGAGATA      1200
NCAA01003621.1      ATGCTGAAACTTTATCAATCTGTTTCCAATATTACCTGCGGCAGTGCTGATGAAGAGATA      1200
AWOJ01110029.1      ATGCTGAAACTTTATCAATCTGTTTCCAATATTACCTGCGGCAGTGCTGATGAAGAGATA      1200
                    ************************************************************

NW_015887304.1      GAAGGCTGCCGAGACAGCTCGGCTGCTCTTACCCTTAAAATCGTGGCTATCTCTGCCATC      1260
NCAA01003621.1      GAAGGCTGCCGAGACAGCTCGGCTGCTCTTACCCTTAAAATCGTGGCTATCTCTGCCATC      1260
AWOJ01110029.1      GAAGGCTGCCGAGACAGCTCGGCTGCTCTTACCCTTAAAATCGTGGCTATCTCTGCCATC      1260
                    ************************************************************

NW_015887304.1      CTAATAGCTAGTACTTGCGGAGTTGGTATCCCGTTAGTTGGCAAGAAGCATCGGTTCCTC      1320
NCAA01003621.1      CTAATAGCTAGTACTTGCGGAGTTGGTATCCCGTTAGTTGGCAAGAAGCATCGGTTCCTC      1320
AWOJ01110029.1      CTAATAGCTAGTACTTGCGGAGTTGGTATCCCGTTAGTTGGCAAGAAGCATCGGTTCCTC      1320
                    ************************************************************

NW_015887304.1      CGAACTGACTCTAATCTCTTTCTTGCTGTTAAAGCCTTTGCTGCTGGTGTCATCCTCTCT      1380
NCAA01003621.1      CGAACTGACTCTAATCTCTTTCTTGCTGTTAAAGCCTTTGCTGCTGGTGTCATCCTCTCT      1380
AWOJ01110029.1      CGAACTGACTCTAATCTCTTTCTTGCTGTTAAAGCCTTTGCTGCTGGTGTCATCCTCTCT      1380
                    ************************************************************

NW_015887304.1      ACAGGCTTTGTCCACATATTACCAGGCGCCACCTCATCATTAACTAATCCTTGTCTTCCG      1440
NCAA01003621.1      ACAGGCTTTGTCCACATATTACCAGGCGCCACCTCATCATTAACTAATCCTTGTCTTCCG      1440
AWOJ01110029.1      ACAGGCTTTGTCCACATATTACCAGGCGCCACCTCATCATTAACTAATCCTTGTCTTCCG      1440
                    ************************************************************

NW_015887304.1      AAATCTCCTTGGTTGAAATTCCCTTTCGCTGGTTTTATCGCCATGATGGCTGCATTGACT      1500
```

```
NCAA01003621.1      AAATCTCCTTGGTTGAAATTCCCTTTCGCTGGTTTTATCGCCATGATGGCTGCATTGACT      1500
AWOJ01110029.1      AAATCTCCTTGGTTGAAATTCCCTTTCGCTGGTTTTATCGCCATGATGGCTGCATTGACT      1500
                    ************************************************************

NW_015887304.1      ACCTTGGTGGTTGACTTTGTTGGGACTCAGTATTATGAGAGGAAGCAAGAGAAACAAAGC      1560
NCAA01003621.1      ACCTTGGTGGTTGACTTTGTTGGGACTCAGTATTATGAGAGGAAGCAAGAGAAACAAAGC      1560
AWOJ01110029.1      ACCTTGGTGGTTGACTTTGTTGGGACTCAGTATTATGAGAGGAAGCAAGAGAAACAAAGC      1560
                    ************************************************************

NW_015887304.1      CAAAAAGATCAGATTGATTCAGTGGATTTGGTGTCAGAATCAGCTATTGTACCAGTTGAA      1620
NCAA01003621.1      CAAAAAGATCAGATTGATTCAGTGGATTTGGTGTCAGAATCAGCTATTGTACCAGTTGAA      1620
AWOJ01110029.1      CAAAAAGATCAGATTGATTCAGTGGATTTGGTGTCAGAATCAGCTATTGTACCAGTTGAA      1620
                    ************************************************************

NW_015887304.1      CCAAAGGCAGGGAATGAGAAATTGTTTGGTGAAGAAGATGGTGGTGCAATACACATTGTT      1680
NCAA01003621.1      CCAAAGGCAGGGAATGAGAAATTGTTTGGTGAAGAAGATGGTGGTGCAATACACATTGTT      1680
AWOJ01110029.1      CCAAAGGCAGGGAATGAGAAATTGTTTGGTGAAGAAGATGGTGGTGCAATACACATTGTT      1680
                    ************************************************************

NW_015887304.1      GGGATGCATGCACATGCAGCTCATCACAGACATAGCCATTCACAAGAACAAGGGGCATGT      1740
NCAA01003621.1      GGGATGCATGCACATGCAGCTCATCACAGACATAGCCATTCACAAGAACAAGGGGCATGT      1740
AWOJ01110029.1      GGGATGCATGCACATGCAGCTCATCACAGACATAGCCATTCACAAGAACAAGGGGCATGT      1740
                    ************************************************************

NW_015887304.1      CAAGGGAACGTGAGGGAGCATTCCCATGGTCATTCGCACTCCCATAGCTTTGGTGGTGGA      1800
NCAA01003621.1      CAAGGGAACGTGAGGGAGCATTCCCATGGTCATTCGCACTCCCATAGCTTTGGTGGTGGA      1800
AWOJ01110029.1      CAAGGGAACGTGAGGGAGCATTCCCATGGTCATTCGCACTCCCATAGCTTTGGTGGTGGA      1800
                    ************************************************************

NW_015887304.1      GATGAGGAAGGTGGAGGGAGGCATGTTGTTGTTTCTCAGGTAATTTTACTTGCATATTTT      1860
NCAA01003621.1      GATGAGGAAGGTGGAGGGAGGCATGTTGTTGTTTCTCAGGTAATTTTACTTGCATATTTT      1860
AWOJ01110029.1      GATGAGGAAGGTGGAGGGAGGCATGTTGTTGTTTCTCAGGTAATTTTACTTGCATATTTT      1860
                    ************************************************************

NW_015887304.1      TACATGTCTTAGGCCTTTCTCTGTACTTCTCCACTTAAGTTCGTTCTGGTTGGATTGGGT      1920
NCAA01003621.1      TACATGTCTTAGGCCTTTCTCTGTACTTCTCCACTTAAGTTCGTTCTGGTTGGATTGGGT      1920
AWOJ01110029.1      TACATGTCTTAGGCCTTTCTCTGTACTTCTCCACTTAAGTTCGTTCTGGTTGGATTGGGT      1920
                    ************************************************************

NW_015887304.1      AAATGTTGTGTCTAGAAGGTTTTTGATTATCTCATTGGTTGTAGGTTTTAAAGATATGAA      1980
NCAA01003621.1      AAATGTTGTGTCTAGAAGGTTTTTGATTATCTCATTGGTTGTAGGTTTTAAAGATATGAA      1980
AWOJ01110029.1      AAATGTTGTGTCTAGAAGGTTTTTGATTATCTCATTGGTTGTAGGTTTTAAAGATATGAA      1980
                    ************************************************************

NW_015887304.1      TTACATTTTCTCGATGTCAGGATTTATGAGAAAGAAAATGTTTGGGCTTCATATATATCA      2040
NCAA01003621.1      TTACATTTTCTCGATGTCAGGATTTATGAGAAAGAAAATGTTTGGGCTTCATATATATCA      2040
AWOJ01110029.1      TTACATTTTCTCGATGTCAGGATTTATGAGAAAGAAAATGTTTGGGCTTCATATATATCA      2040
                    ************************************************************

NW_015887304.1      TGCTGGTAAACTTGATACAGAATTTTCTGAACGACTTGTAGTATGAATGGTTTACCAAAT      2100
NCAA01003621.1      TGCTGGTAAACTTGATACAGAATTTTCTGAACGACTTGTAGTATGAATGGTTTACCAAAT      2100
AWOJ01110029.1      TGCTGGTAAACTTGATACAGAATTTTCTGAACGACTTGTAGTATGAATGGTTTACCAAAT      2100
                    ************************************************************

NW_015887304.1      TTACTACTTATAGGTTTTTGCGGAATTAATATAGAGAATTCATATAACCAACTCTTAGGG      2160
NCAA01003621.1      TTACTACTTATAGGTTTTTGCGGAATTAATATAGAGAATTCATATAACCAACTCTTAGGG      2160
AWOJ01110029.1      TTACTACTTATAGGTTTTTGCGGAATTAATATAGAGAATTCATATAACCAACTCTTAGGG      2160
                    ************************************************************

NW_015887304.1      ATTGAGACATAGTTGATTGATTGCAACTGCTTATATGCTACTGATTCCTTGAGCTTTGAT      2220
NCAA01003621.1      ATTGAGACATAGTTGATTGATTGCAACTGCTTATATGCTACTGATTCCTTGAGCTTTGAT      2220
AWOJ01110029.1      ATTGAGACATAGTTGATTGATTGCAACTGCTTATATGCTACTGATTCCTTGAGCTTTGAT      2220
                    ************************************************************

NW_015887304.1      AAATGCATTTCTATAAGCGTATGCGGAATTACACTATTTCATTTTCTTCTAGGTTTAGCT      2280
NCAA01003621.1      AAATGCATTTCTATAAGCGTATGCGGAATTACACTATTTCATTTTCTTCTAGGTTTAGCT      2280
AWOJ01110029.1      AAATGCATTTCTATAAGCGTATGCGGAATTACACTATTTCATTTTCTTCTAGGTTTAGCT      2280
                    ************************************************************

NW_015887304.1      AGATATTCTGCTAAATTATTTTCTAATTGTTTGGTGTTTAAGATTTTTCACCAACCTTTG      2340
NCAA01003621.1      AGATATTCTGCTAAATTATTTTCTAATTGTTTGGTGTTTAAGATTTTTCACCAACCTTTG      2340
AWOJ01110029.1      AGATATTCTGCTAAATTATTTTCTAATTGTTTGGTGTTTAAGATTTTTCACCAACCTTTG      2340
                    ************************************************************

NW_015887304.1      AGATACTGCATATATTGTAATATGAAATCTAACTTCACGTAGAATATTACCTTAAGTTTG      2400
NCAA01003621.1      AGATACTGCATATATTGTAATATGAAATCTAACTTCACGTAGAATATTACCTTAAGTTTG      2400
AWOJ01110029.1      AGATACTGCATATATTGTAATATGAAATCTAACTTCACGTAGAATATTACCTTAAGTTTG      2400
```

```
                  ************************************************************

NW_015887304.1    CTAGCCATTCGTTGGCCTATAGGGTGCCCATCGTTAGATATTTTGGTGTGGTGTGTGTCA    2460
NCAA01003621.1    CTAGCCATTCGTTGGCCTATAGGGTGCCCATCGTTAGATATTTTGGTGTGGTGTGTGTCA    2460
AWOJ01110029.1    CTAGCCATTCGTTGGCCTATAGGGTGCCCATCGTTAGATATTTTGGTGTGGTGTGTGTCA    2460
                  ************************************************************

NW_015887304.1    GTTCACAGAATGGTTTATGCATTCACAGACTAGGGATTTGCTTATGGTTTCTCATAATAG    2520
NCAA01003621.1    GTTCACAGAATGGTTTATGCATTCACAGACTAGGGATTTGCTTATGGTTTCTCATAATAG    2520
AWOJ01110029.1    GTTCACAGAATGGTTTATGCATTCACAGACTAGGGATTTGCTTATGGTTTCTCATAATAG    2520
                  ************************************************************

NW_015887304.1    CAGATTCTTGGGGGAGCTTGTTTCTTTAATGTATTACAAGACCGGAACCAACTTTTTACT    2580
NCAA01003621.1    CAGATTCTTGGGGGAGCTTGTTTCTTTAATGTATTACAAGACCGGAACCAACTTTTTACT    2580
AWOJ01110029.1    CAGATTCTTGGGGGAGCTTGTTTCTTTAATGTATTACAAGACCGGAACCAACTTTTTACT    2580
                  ************************************************************

NW_015887304.1    TTTGTTTAATTGGCAAATGAGCAAAAGTTTCATTACTCAAAGTTTGATTTTCCCCGTGTT    2640
NCAA01003621.1    TTTGTTTAATTGGCAAATGAGCAAAAGTTTCATTACTCAAAGTTTGATTTTCCCCGTGTT    2640
AWOJ01110029.1    TTTGTTTAATTGGCAAATGAGCAAAAGTTTCATTACTCAAAGTTTGATTTTCCCCGTGTT    2640
                  ************************************************************

NW_015887304.1    TGTGAAGATACATTGTTTGTTTATACCTGTCAGTTTGATTGTGAAACTTGGTCATTATAA    2700
NCAA01003621.1    TGTGAAGATACATTGTTTGTTTATACCTGTCAGTTTGATTGTGAAACTTGGTCATTATAA    2700
AWOJ01110029.1    TGTGAAGATACATTGTTTGTTTATACCTGTCAGTTTGATTGTGAAACTTGGTCATTATAA    2700
                  ************************************************************

NW_015887304.1    CGACTTTCTTGTATGCAGGTCTTGGAGCTGGGAATAGTATCACATTCTCTCATAATAGGC    2760
NCAA01003621.1    CGACTTTCTTGTATGCAGGTCTTGGAGCTGGGAATAGTATCACATTCTCTCATAATAGGC    2760
AWOJ01110029.1    CGACTTTCTTGTATGCAGGTCTTGGAGCTGGGAATAGTATCACATTCTCTCATAATAGGC    2760
                  ************************************************************

NW_015887304.1    ATAGCATTGGGTGTTTCAGAAAGTCCATGCACAATTAGACCCTTGCTCGTGGCCTTATCG    2820
NCAA01003621.1    ATAGCATTGGGTGTTTCAGAAAGTCCATGCACAATTAGACCCTTGCTCGTGGCCTTATCG    2820
AWOJ01110029.1    ATAGCATTGGGTGTTTCAGAAAGTCCATGCACAATTAGACCCTTGCTCGTGGCCTTATCG    2820
                  ************************************************************

NW_015887304.1    TTCCACCAGTTCTTCGAAGGTTTTGCGTTAGGAGGTTGCATCTCGCAGGCACAGTTCAAT    2880
NCAA01003621.1    TTCCACCAGTTCTTCGAAGGTTTTGCGTTAGGAGGTTGCATCTCGCAGGCACAGTTCAAT    2880
AWOJ01110029.1    TTCCACCAGTTCTTCGAAGGTTTTGCGTTAGGAGGTTGCATCTCGCAGGCACAGTTCAAT    2880
                  ************************************************************

NW_015887304.1    TCCCTCCGTTCCACTATAATGGCAACGTTTTTCGCCGTAACAACACCCTTGGGAATTGCT    2940
NCAA01003621.1    TCCCTCCGTTCCACTATAATGGCAACGTTTTTCGCCGTAACAACACCCTTGGGAATTGCT    2940
AWOJ01110029.1    TCCCTCCGTTCCACTATAATGGCAACGTTTTTCGCCGTAACAACACCCTTGGGAATTGCT    2940
                  ************************************************************

NW_015887304.1    ATAGGAATTCTAGCTTCTTCATCTTACAATCCACATAGCCCAAGAGCTTTGGTAGTGGAA    3000
NCAA01003621.1    ATAGGAATTCTAGCTTCTTCATCTTACAATCCACATAGCCCAAGAGCTTTGGTAGTGGAA    3000
AWOJ01110029.1    ATAGGAATTCTAGCTTCTTCATCTTACAATCCACATAGCCCAAGAGCTTTGGTAGTGGAA    3000
                  ************************************************************

NW_015887304.1    GGGAGCCTTAACTCTATATCTGCTGGAATTTTAATCTACATGGCTTTAGTAGACCTAATT    3060
NCAA01003621.1    GGGAGCCTTAACTCTATATCTGCTGGAATTTTAATCTACATGGCTTTAGTAGACCTAATT    3060
AWOJ01110029.1    GGGAGCCTTAACTCTATATCTGCTGGAATTTTAATCTACATGGCTTTAGTAGACCTAATT    3060
                  ************************************************************

NW_015887304.1    GCTGCAGATTTCTTGAGTAAAAGAATGAGCTGCAATACAAGGCTTCAAATAGTATCTTAT    3120
NCAA01003621.1    GCTGCAGATTTCTTGAGTAAAAGAATGAGCTGCAATACAAGGCTTCAAATAGTATCTTAT    3120
AWOJ01110029.1    GCTGCAGATTTCTTGAGTAAAAGAATGAGCTGCAATACAAGGCTTCAAATAGTATCTTAT    3120
                  ************************************************************

                                                                86460
NW_015887304.1    TTTGCACTATTCTTAGGGGCTAGACTCATGTCCCTTCTTGCAATATGGGCATGA    3174
                                                               102832
NCAA01003621.1    TTTGCACTATTCTTAGGGGCTGGACTCATGTCCCTTCTTGCAATATGGGCATGA    3174
                                                                17643
AWOJ01110029.1    TTTGCACTATTCTTAGGGGCTGGACTCATGTCCCTTCTTGCAATATGGGCATGA    3174
                  ********************* ********************************
```
