# Supplementary material for: Functional Analysis of NtZIP4B and Zn Status-Dependent Expression Pattern of Tobacco ZIP Genes
Source: Front Plant Sci. 2019 Jan 10;9:1984. doi: 10.3389/fpls.2018.01984 (PMC6335357; doi:10.3389/fpls.2018.01984)
Supplement: FILE S5 — Summary information on mRNA and genomic sequences of NtZIP4A and NtZIP4B; sequence identity between NtZIP4A, NtZIP4B, and ZIP predicted proteins from selected species. [file Data_Sheet_5.PDF]

Supplementary File S5

Content:

A. Summary information on mRNA and genomic sequences of *NtZIP4A* and *NtZIP4B*.
B. Sequence identity between the NtZIP4A, NtZIP4B and ZIP4 predicted proteins from selected species

A. Summary information on mRNA and genomic sequences of *NtZIP4A* and *NtZIP4B* (including cultivars NT90, Basma Xanthi, K326, K326 Nitab4.5_0003621).
in bold - mRNA Accession Number , NCBI genomic reference sequence Accesion Number, contig number
ORF – Open Reading Frame

	NtZIP4A	NtZIP4B
mRNA reference sequence Acc No	**XM_016647965.1**	**XM_016586154.1**
Gene symbol	LOC107821537	LOC107767236
Lenght of mRNA reference sequence	1997 bp	1816 bp
ORF	1236 bp	1236 bp
Protein lenght	412 aa	412 aa
Acc No of NCBI genomic reference sequence (cultivar NT90)<br>It includes the genomic copy with given ORF START site, ORF-end (the last base of ORF), and the length of ORF with introns	**NW_015828584.1**<br><br>ORF START - 4717<br>ORF end – 8152<br>Length of ORF + introns – 3436 bp	**NW_015887304.1**<br><br>START codon - 83290<br>ORF end – 86460<br>Length of ORF + introns – 3171 bp
Contig no for cultivar NT90,<br><br>It cointains genomic sequence with ORF START site, ORF-end (the last base of ORF), and the length of ORF with introns	**AYMY01036928.1**<br><br>ORF START - 4717<br>ORF end – 8152<br><br>Length of ORF + introns – 3436 bp	**AYMY01065187.1**<br><br>START codon - 15666<br>ORF end – 18836<br><br>Length of ORF + introns – 3171 bp
Contig no for cultivar Basma Xanthi,<br><br>It cointains genomic sequence with ORF START site, ORF-end (the last base of ORF), and the length of ORF with introns	For Basma Xanthi sequences homologous for ORF of *NtZIP4A* are present on different contigs:<br>**AWOK01066417.1**<br>START codon - 4749<br>**AWOK01066418.1** – part of the ORF<br>**AWOK01539318.1 -** part of the last exon;<br>ORF end - 8029	For Basma Xanthi sequences homologous for ORF of *NtZIP4B* are present on different contigs:<br>**AWOK01166346.1**<br>START codon - 12567<br>**AWOK01539318.1 -** part of the last exon<br>ORF end - 8262
Contig no for cultivar K326 ,<br><br>It cointains genomic sequence with ORF START site, ORF-end (the last base of ORF), and the length of ORF with introns	**AWOJ01064181.1**<br><br>START codon - 26070<br>ORF end – 29619<br>Lenght of ORF + introns – 3550 bp	**AWOJ J01110029.1**<br><br>START codon - 14413<br>ORF end – 17643<br>Lenght of ORF + introns – 3231 bp
Contig no for cultivar K326 Nitab4.5_0003621 ,<br><br>It cointains genomic sequence with ORF START site, ORF-end (the last base of ORF), and the length of ORF with introns	**NCAA01001367.1** orientacja plus/minus<br><br>START codon - 134310<br>ORF end – 130875<br>Lenght of ORF + introns	**NCAA01003621.1**<br><br>START codon - 99664<br>ORF end – 102834<br>Lenght of ORF + introns – 3171 bp

Similarity between ORF mRNA – 96,52% (acc. Clustal Omega)
Similarity between genomic sequences (ORF + introns – without STOP codon) – 94.62%*
Podobieństwo pomiędzy sekwencjami białkowymi - 97.57% (wg. Clustal Omega)

* For comparison the following sequences were used:
the *NtZIP4A* sequence originated from the genomic sequence no NW_015828584.1 (4717..8152);
the NtZIP4B sequence originated from the genomic sequence from contig AWOJ J01110029.1 (14413..17643). The sequence of the cloned NtZIP4B is identical to the sequence from the contig AWOJ J01110029.1 (14413..17643);

B. Sequence identity between the NtZIP4A,NtZIP4B and ZIP4 predicted proteins from selected species (sequences were chosen based on phylogenetic tree given in Figure 1), using Clustal Omega https://www.ebi.ac.uk/Tools/msa/clustalo/

	NtZIP4B	AtZIP4	NtomZIP4	NsZIP4	NaZIP4	SlZIP4	AtZIP9	VvZIP4	GmZIP4
**NtZIP4A**	97.57	67.02	99.51	97.57	97.30	88.21	56.59	65.36	57.54
**NtZIP4B**	-	67.02	97.33	100.00	98.77	87.22	56.07	65.11	57.82
**AtZIP4**	-	-	67.29	67.02	66.30	69.38	63.29	69.25	66.47
**NtomZIP4**	-	-	-	97.33	97.05	88.21	56.33	65.36	57.54
**NsZIP4**	-	-	-	-	98.77	87.22	56.07	65.11	57.82
**NaZIP4**	-	-	-	-	-	87.31	56.54	64.68	58.19
**SlZIP4**	-	-	-	-	-	-	58.12	66.92	59.50
**AtZIP9**	-	-	-	-	-	-	-	58.51	57.10
**VvZIP4**	-	-	-	-	-	-	-	-	62.95
