# Supplementary material for: Functional Analysis of NtZIP4B and Zn Status-Dependent Expression Pattern of Tobacco ZIP Genes
Source: Front Plant Sci. 2019 Jan 10;9:1984. doi: 10.3389/fpls.2018.01984 (PMC6335357; doi:10.3389/fpls.2018.01984)
Supplement: FILE S6 — Bioinformatics analysis of NtZIP4B subcellular localization. [file Data_Sheet_6.PDF]

Supplementary File S6

Indication of the subcellular localization of the NtZIP4B with the use of ProtComp v. 9.0 online
http://www.softberry.com/berry.phtml?topic=protcomppl&group=programs&subgroup=proloc

The sequence of NtZIP4B

```
MSFTEDLVPFFFMDPKIREKTGAFSDTVMLKLYQSVSNITCGSADEEIEGCRDSSAALTLKIVAISAILI
ASTCGVGIPLVGKKHRFLRTDSNLFLAVKAFAAGVILSTGFVHILPGATSSLTNPCLPKSPWLKFPFAGF
IAMMAALTTLVVDFVGTQYYERKQEKQSQKDQIDSVDLVSESAIVPVEPKAGNEKLFGEEDGGAIHIVGM
HAHAAHHRHSHSQEQGACQGNVREHSHGHSHSHSFGGGDEEGGGRHVVVSQVLELGIVSHSLIIGIALGV
SESPCTIRPLLVALSFHQFFEGFALGGCISQAQFNSLRSTIMATFFAVTTPLGIAIGILASSSYNPHSPR
ALVVEGSLNSISAGILIYMALVDLIAADFLSKRMSCNTRLQIVSYFALFLGAGLMSLLAIWA
```

As a control the following transport proteins were included into analysis:

>NP_195444.1 heavy metal atpase 1 [*Arabidopsis thaliana*]
AtHMA1

```
MEPATLTRSSSLTRFPYRRGLSTLRLARVNSFSILPPKTLLRQKPLRISASLNLPPRSIRLRAVEDHHHD
HHHDDEQDHHNHHHHHQHGCCSVELKAESKPQKMLFGFAKAIGWVRLANYLREHLHLCCSAAAMFLAAA
VCPYLAPEPYIKSLQNAFMIVGFPLVGVSASLDALMDIAGGKVNIHVLMALAAFASVFMGNALEGGLLLA
MFNLAHIAEEFFTSRSMVDVKELKESNPDSALLIEVHNGNVPNISDLSYKSVPVHSVEVGSYVLVGTGEI
VPVDCEVYQGSATITIEHLTGEVKPLEAKAGDRVPGGARNLDGRMIVKATKAWNDSTLNKIVQLTEEAHS
NKPKLQRWLDEFGENYSKVVVVLSLAIAFLGPFLFKWPFLSTAACRGSVYRALGLMVAASPCALAVAPLA
YATAISSCARKGILLKGAQVLDALASCHTIAFDKTGTLTTGGLTCKAIEPIYGHQGGTNSSVITCCIPNC
EKEALAVAAAMEKGTTHPIGRAVVDHSVGKDLPSIFVESFEYFPGRGLTATVNGVKTVAEESRLRKASLG
SIEFITSLFKSEDESKQIKDAVNASSYGKDFVHAALSVDQKVTLIHLEDQPRPGVSGVIAELKSWARLRV
MMLTGDHDSSAWRVANAVGITEVYCNLKPEDKLNHVKNIAREAGGGLIMVGEGINDAPALAAATVGIVLA
QRASATAIAVADILLLRDNITGVPFCVAKSRQTTSLVKQNVALALTSIFLAALPSVLGFVPLWLTVLLHE
GGTLLVCLNSVRGLNDPSWSWKQDIVHLINKLRSQEPTSSSSNSLSSAH
```

>NP_178286.1 vacuolar iron transporter 1 [*Arabidopsis thaliana*]
AtVIT1

```
MSSEEDKITRISIEPEKQTLLDHHTEKHFTAGEIVRDIIIGVSDGLTVPFALAAGLSGANASSSIVLTAG
IAEVAAGAISMGLGGYLAAKSEEDHYAREMKREQEEIVAVPETEAAEVAEILAQYGIEPHEYSPVVNALR
KNPQAWLDFMMRFELGLEKPDPKRALQSAFTIAIAYVLGGFIPLLPYMLIPHAMDAVVASVVITLFALFI
FGYAKGHFTGSKPLRSAFETAFIGAIASAAAFCLAKVVQH
```

>NP_001324595.1 zinc transporter [*Arabidopsis thaliana*]
>AtMTP1

```
MESSSPHHSHIVEVNVGKSDEERIIVASKVCGEAPCGFSDSKNASGDAHERSASMRKLCIAVVLCLVFMS
VEVVGGIKANSLAILTDAAHLLSDVAAFAISLFSLWAAGWEATPRQTYGFFRIEILGALVSIQLIWLLTG
ILVYEAIIRIVTETSEVNGFLMFLVAAFGLVVNIIMAVLLGHDHGHSHGHGHGHGHDHHNHSHGVTVTTH
HHHHDHEHGHSHGHGEDKHHAHGDVTEQLLDKSKTQVAAKEKRKRNINLQGAYLHVLGDSIQSVGVMIGG
AIIWYNPEWKIVDLICTLAFSVIVLGTTINMIRNILEVLMESTPREIDATKLEKGLLEMEEVVAVHELHI
WAITVGKVLLACHVNIRPEADADMVLNKVIDYIRREYNISHVTIQIER
```

Results of the bioinformatics analysis:

NtZIP4B

```
5724 multiple located sequences are accepted
ProtComp Version 9.0. Identifying sub-cellular location (Plant)
Seq name:  test sequence, Length=412
```

Significant similarity in Location DB - Plasma membrane
Database sequence: AC=Q6ZJ91 Location:Plasma membrane DE Zinc transporter 4;||37
Score=88, Sequence length=397, Alignment length=351
Predicted by Neural Nets - Extracellular (Secreted) with score 0.9
******** Transmembrane segments are found: .-55:83+..+93:121+..+132:158-..-265:283+..+299:313+..+320:346o..o356:381+.
******** Potential GPI-anchor in position 386 is found
Integral Prediction of protein location: Plasma membrane with score 8.7

Location weights:	LocDB /	PotLocDB /	Neural Nets /	Pentamers /	Integral
Nuclear	0.0 /	0.0 /	0.00 /	0.00 /	0.00
Plasma membrane	10.0 /	1.4 /	0.89 /	0.11 /	8.68
Extracellular	0.0 /	0.0 /	0.89 /	0.00 /	0.18
Cytoplasmic	0.0 /	0.0 /	0.00 /	0.17 /	0.05
Mitochondrial	0.0 /	0.0 /	0.00 /	2.20 /	0.00
Endoplasm. retic.	0.0 /	0.0 /	0.00 /	1.01 /	0.00
Peroxisomal	0.0 /	0.0 /	0.89 /	0.00 /	0.05
Golgi	0.0 /	0.0 /	0.34 /	0.12 /	0.00
Chloroplast	0.0 /	1.6 /	0.00 /	1.90 /	1.03
Vacuolar	0.0 /	0.0 /	0.00 /	0.00 /	0.00

AtHMA1

5724 multiple located sequences are accepted
ProtComp Version 9.0. Identifying sub-cellular location (Plant)
Seq name: test sequence, Length=819
Significant similarity in Potential Location DB - Chloroplast
Database sequence: AC=Q9M3H5 Location:Chloroplast DE Probable cadmium/zinc-trans
Score=100, Sequence length=820, Alignment length=819
Predicted by Neural Nets - Extracellular (Secreted) with score 0.9
******** Chloroplast Transit peptide 1-34 is found
Integral Prediction of protein location: Membrane bound Chloroplast with score 8.4

Location weights:	LocDB /	PotLocDB /	Neural Nets /	Pentamers /	Integral
Nuclear	0.0 /	0.0 /	0.00 /	0.00 /	0.00
Plasma membrane	0.0 /	0.0 /	0.87 /	0.00 /	0.00
Extracellular	0.0 /	0.0 /	0.87 /	0.00 /	1.37
Cytoplasmic	0.0 /	0.0 /	0.00 /	0.00 /	0.00
Mitochondrial	0.0 /	0.0 /	0.00 /	0.44 /	0.10
Endoplasm. retic.	0.0 /	0.0 /	0.00 /	0.00 /	0.00
Peroxisomal	0.0 /	0.0 /	0.87 /	0.00 /	0.13
Golgi	0.0 /	0.0 /	0.39 /	0.15 /	0.03
Chloroplast	0.0 /	5.0 /	0.00 /	5.18 /	8.37
Vacuolar	0.0 /	0.0 /	0.00 /	0.00 /	0.00

AtVIT1

5724 multiple located sequences are accepted
ProtComp Version 9.0. Identifying sub-cellular location (Plant)
Seq name: test sequence, Length=250
Significant similarity in Location DB - Vacuole
Database sequence: AC=Q9ZUA5 Location:Vacuole DE Vacuolar iron transporter 1;||1
Score=100, Sequence length=251, Alignment length=250
Predicted by Neural Nets - Extracellular (Secreted) with score 0.9
Integral Prediction of protein location: Membrane bound Vacuolar with score 9.2

Location weights:	LocDB /	PotLocDB /	Neural Nets /	Pentamers /	Integral

Nuclear	0.0 /	0.0 /	0.00 /	0.00 /	0.00
Plasma membrane	0.0 /	0.0 /	0.91 /	0.00 /	0.56
Extracellular	0.0 /	0.0 /	0.91 /	0.00 /	0.00
Cytoplasmic	0.0 /	0.0 /	0.00 /	0.00 /	0.00
Mitochondrial	0.0 /	0.0 /	0.00 /	0.55 /	0.05
Endoplasm. retic.	0.0 /	0.0 /	0.00 /	0.36 /	0.00
Peroxisomal	0.0 /	0.0 /	0.91 /	0.00 /	0.06
Golgi	0.0 /	0.0 /	0.27 /	0.00 /	0.07
Chloroplast	0.0 /	0.0 /	0.00 /	0.00 /	0.00
**Vacuolar**	**10.0 /**	**3.0 /**	**0.00 /**	**5.80 /**	**9.24**

AtMTP1

5724 multiple located sequences are accepted
ProtComp Version 9.0. Identifying sub-cellular location (Plant)
Seq name: test sequence, Length=398
Significant similarity in Location DB - Vacuole
Database sequence: AC=Q2HJ10 Location:Vacuole DE Zinc transporter 2;
Score=79, Sequence length=372, Alignment length=294
Predicted by Neural Nets - Extracellular (Secreted) with score   0.9
Integral Prediction of protein location: Membrane bound Vacuolar with score 9.1

Location weights:	LocDB /	PotLocDB /	Neural Nets /	Pentamers /	Integral
Nuclear	0.0 /	0.0 /	0.00 /	0.00 /	0.00
Plasma membrane	0.0 /	0.0 /	0.88 /	0.14 /	0.13
Extracellular	0.0 /	0.0 /	0.88 /	2.05 /	0.25
Cytoplasmic	0.0 /	0.0 /	0.00 /	1.10 /	0.00
Mitochondrial	0.0 /	0.0 /	0.00 /	1.91 /	0.45
Endoplasm. retic.	0.0 /	0.0 /	0.00 /	0.46 /	0.07
Peroxisomal	0.0 /	0.0 /	0.88 /	0.00 /	0.03
Golgi	0.0 /	0.0 /	0.37 /	0.00 /	0.00
Chloroplast	0.0 /	0.0 /	0.00 /	0.17 /	0.02
**Vacuolar**	**10.0 /**	**0.0 /**	**0.00 /**	**0.03 /**	**9.06**
